# Multimodal subspace independent vector analysis effectively captures latent relationships between brain structure and function

**DOI:** 10.1162/IMAG.a.1266

**Published:** 2026-06-18

**Authors:** Xinhui Li, Peter Kochunov, Tulay Adali, Rogers F. Silva, Vince D. Calhoun

**Affiliations:** Tri-institutional Center for Translational Research in Neuroimaging and Data Science, Georgia State University, Georgia Institute of Technology, Emory University, Atlanta, GA, United States; School of Electrical and Computer Engineering, Georgia Institute of Technology, Atlanta, GA, United States; Department of Psychiatry and Behavioral Sciences, McGovern Medical School, University of Texas Health Science Center at Houston, Houston, TX, United States; Department of Computer Science and Electrical Engineering, University of Maryland Baltimore County, Baltimore, MD, United States

**Keywords:** multimodal fusion, sMRI, fMRI, biomarker, age, sex, schizophrenia

## Abstract

A key challenge in neuroscience is inferring relationships between brain structure and function from high-dimensional, multimodal neuroimaging data. While conventional multivariate approaches often simplify statistical assumptions and estimate one-dimensional independent sources shared across modalities, the true relationships between latent sources are likely more complex—statistical dependence may exist both within and between modalities and span more than one dimension per modality. Here, we introduce Multimodal Subspace Independent Vector Analysis (MSIVA), a method for capturing both joint and unique vector sources from multiple data modalities by defining cross-modal and unimodal subspaces with variable dimensions. MSIVA enables flexible estimation of varying-size independent subspaces within modalities and their one-to-one linkage to corresponding subspaces across modalities. Crucially, it captures subject-level variability at the voxel level within independent subspaces, in contrast to traditional methods that share identical independent components across subjects. We evaluated three initialization workflows with five candidate subspace structures in multiple synthetic datasets and two large multimodal neuroimaging datasets, including structural MRI (sMRI) and functional MRI (fMRI). After confirming that MSIVA successfully recovered ground-truth subspace structures in synthetic data, we applied MSIVA to identify latent subspace structures in neuroimaging data. Subsequent subspace-specific canonical correlation analysis, brain-phenotype prediction, and voxelwise brain-age delta analysis revealed that MSIVA sources were strongly associated with multiple phenotype variables, including age, sex, schizophrenia, lifestyle factors, and cognitive functions. Further, we identified modality- and group-specific brain regions related to age (for example, cerebellum, precentral gyrus, and cingulate gyrus in sMRI; occipital lobe and superior frontal gyrus in fMRI), sex (for example, cerebellum in sMRI, frontal lobe in fMRI, and precuneus in both sMRI and fMRI), and schizophrenia (for example, cerebellar, frontal, and insular cortices in sMRI; occipital pole, lingual gyrus, and precuneus in fMRI), shedding light on linked phenotypic and neuropsychiatric biomarkers of brain structure and function.

## 1 Introduction

Neuroimaging techniques such as magnetic resonance imaging (MRI) have been developed to understand structural and functional properties of the brain, as well as their relationships to behavior. However, it is challenging to directly associate behavioral measures with raw MRI data, which typically include tens of thousands of voxels across hundreds or thousands of subjects. Although neural data appear complex in their original space, their intrinsic dimensionality is often significantly lower. Recent studies have found that neural representations in low-dimensional subspaces form the computational basis for motor functions such as reaching (Churchland et al., 2012; Pandarinath et al., 2018) and timing (Remington et al., 2018; Wang et al., 2018), as well as cognitive functions such as perception (Bao et al., 2020; Chang & Tsao, 2017; Semedo et al., 2019; She et al., 2024), generalization (Bernardi et al., 2020; Boyle et al., 2024; Courellis et al., 2024), and decision-making (Hajnal et al., 2024; Johnston et al., 2024). These findings highlight the importance of developing latent variable models to learn low-dimensional representations and structures from high-dimensional data. In addition, each neuroimaging modality has its own strengths and weaknesses and only captures limited information about the brain. For example, structural MRI (sMRI) provides high-resolution anatomical details of the brain but does not capture temporal dynamics, while functional MRI (fMRI) measures blood-oxygenation-level-dependent (BOLD) signals over time at the cost of lower spatial resolution. Joint analysis of sMRI and fMRI offers richer spatiotemporal information about the brain than either modality alone. With the increasing availability of multimodal neuroimaging datasets, it is necessary to develop multimodal fusion approaches to effectively capture interpretable and complementary information about the brain and related disorders from multiple imaging modalities (Calhoun & Sui, 2016; Lahat et al., 2015; Sui et al., 2012; Zhang et al., 2020).

A variety of multivariate fusion methods have been developed to jointly analyze multiple neuroimaging datasets or data modalities, including joint independent component analysis (jICA) (Calhoun & Adali, 2009; Calhoun, Adali, Giuliani, et al., 2006; Calhoun, Adali, Pearlson, et al., 2006; Franco et al., 2008), linked ICA (Groves et al., 2011), multimodal canonical correlation analysis (mCCA) (Correa et al., 2008, 2010; Mohammadi-Nejad et al., 2017), jICA+mCCA (Sui et al., 2011, 2013), and independent vector analysis (IVA) (Adali et al., 2015a, 2015b). Notably, a unified framework, Multidataset Independent Subspace Analysis (MISA) (Silva et al., 2021), encompasses multiple latent variable models, such as ICA (Comon, 1994), IVA (Adali et al., 2014; Kim et al., 2006), and independent subspace analysis (ISA) (Cardoso, 1998; Hyvärinen & Hoyer, 2000; Szabó et al., 2012). MISA can be applied to identify latent sources from multiple neuroimaging modalities, including sMRI and fMRI (Silva et al., 2021). Recently, a multimodal IVA (MMIVA) fusion method built upon MISA has been proposed to identify linked biomarkers related to age, sex, cognition, and psychosis in two large multimodal neuroimaging datasets (Silva et al., 2024). Many existing approaches, including MMIVA, assume that sources are one-dimensional and independent within each modality (i.e., the subspace structure is an identity matrix). However, brain networks are hierarchically organized across multiple spatial and temporal scales (Betzel & Bassett, 2017; Felleman & Van Essen, 1991; Friston, 2008; Kiebel et al., 2008; Zeki & Shipp, 1988). This hierarchical organization implies that the underlying relationships between latent sources are likely more complex—statistical dependence may exist both within and across modalities and span more than one dimension per modality. Sources from the same modality may be linked, potentially grouped by their anatomical or functional properties. For instance, spatial dependence between corresponding sources has been observed in task-based and resting-state fMRI studies (Ma et al., 2010, 2011; McKeown et al., 1998), which MMIVA would not optimally capture.

Aiming to better detect latent relationships from multimodal data, we present Multimodal Subspace Independent Vector Analysis (MSIVA),^1^ a novel multivariate method for capturing linkage of vector sources by defining cross-modal and unimodal subspaces with variable dimensions. MSIVA is built upon MMIVA by defining a block-diagonal matrix as the subspace structure, instead of the identity matrix used in MMIVA. By default, MSIVA is initialized using weight matrices learned from multimodal group principal component analysis (MGPCA) combined with unimodal ICA. MSIVA can simultaneously recover two types of latent sources—those linked across all modalities (along with their underlying relationships) and those unique to a specific modality. Moreover, by leveraging higher-dimensional subspaces, MSIVA sources show greater representation power, which supports downstream analyses at both the subject and voxel levels.

To comprehensively assess MSIVA, we compared three initialization strategies across five candidate subspace structures. We first simulated five synthetic datasets to evaluate whether MSIVA could recover ground-truth subspace structures. We then applied MSIVA to two large multimodal neuroimaging datasets, the UK Biobank (UKB) dataset (Miller et al., 2016) and a schizophrenia (SZ) patient dataset combined from four studies (Aine et al., 2017; Keator et al., 2016; Tamminga et al., 2014). After identifying the optimal subspace structure, we applied canonical correlation analysis (CCA) (Hotelling, 1992) within each cross-modal subspace separately, yielding linked sources with maximum cross-modal correlation. Using these linked sources, we performed age regression, sex classification, and diagnosis classification to investigate their associations with phenotype measures. Finally, we introduced a voxelwise brain-age delta analysis using reconstructed data from MSIVA to examine how the brain-age gap relates to phenotype variables at the voxel level.

In all five synthetic datasets, MSIVA with default initialization successfully recovered ground-truth subspace structures with high-dimensional subspaces. In two independent neuroimaging datasets, MSIVA identified an optimal latent structure consisting of five two-dimensional cross-modal subspaces, providing a more flexible alternative to the one-dimensional subspaces used in MMIVA. Brain-phenotype modeling revealed that post-CCA sources were significantly associated with age, sex, and SZ-related effects. Furthermore, the brain-age gap showed significant correlations with several phenotype measures, including lifestyle factors and cognitive test scores. Together, our findings suggest that MSIVA effectively learns linked, multidimensional latent sources associated with phenotype variables from multimodal neuroimaging data, thereby uncovering linked phenotypic and neuropsychiatric biomarkers of brain structure and function.

## 2 Methods

### 2.1 Multimodal subspace independent vector analysis

We assume that each observed data modality is a linear mixture of latent sources:

$${\text{X}}^{\left[m\right]}={\text{A}}^{\left[m\right]}{\text{S}}^{\left[m\right]},$$ (1)

where ${\text{X}}^{\left[m\right]}\in {\mathbb{R}}^{V\times N}$ is the observed data, ${\text{A}}^{\left[m\right]}\in {\mathbb{R}}^{V\times C}$ is the linear mixing matrix, ${\text{S}}^{\left[m\right]}\in {\mathbb{R}}^{C\times N}$ is the latent source matrix, m is the modality index, V is the number of features, C is the number of latent sources (C<V ), and N is the number of samples. The latent sources across the M modalities are modeled as statistically dependent or independent based on the subspace structure S defined by available *a priori* information. We aim to recover the latent sources ${\hat{\text{S}}}^{\left[m\right]}\in {\mathbb{R}}^{C\times N}$ by estimating a linear unmixing matrix ${\text{W}}^{\left[m\right]}\in {\mathbb{R}}^{C\times V}$ :

$${\hat{\text{S}}}^{\left[m\right]}={\text{W}}^{\left[m\right]}{\text{X}}^{\left[m\right]}.$$ (2)

**Fig. 1.**
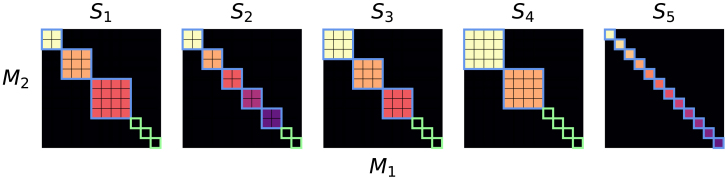
Five plausible candidate subspace structures ($S_{1}-S_{5}$) for two modalities ($M_{1},M_{2}$). Each panel depicts the idealized relationship between sources from two modalities ($M_{1},M_{2}$) for each of five plausible scenarios ($S_{1}-S_{5}$). The side length of each square block is proportional to the number of sources in that subspace. Subspaces highlighted in blue exhibit cross-modal statistical dependence, whereas subspaces highlighted in green (1×1 blocks in $S_{1}-S_{4}$; absent in $S_{5}$) are modality-specific (i.e., no cross-modal correlation). Within each modality, sources belonging to the same subspace are statistically dependent, while sources belonging to different subspaces are statistically independent.

**Fig. 2.**
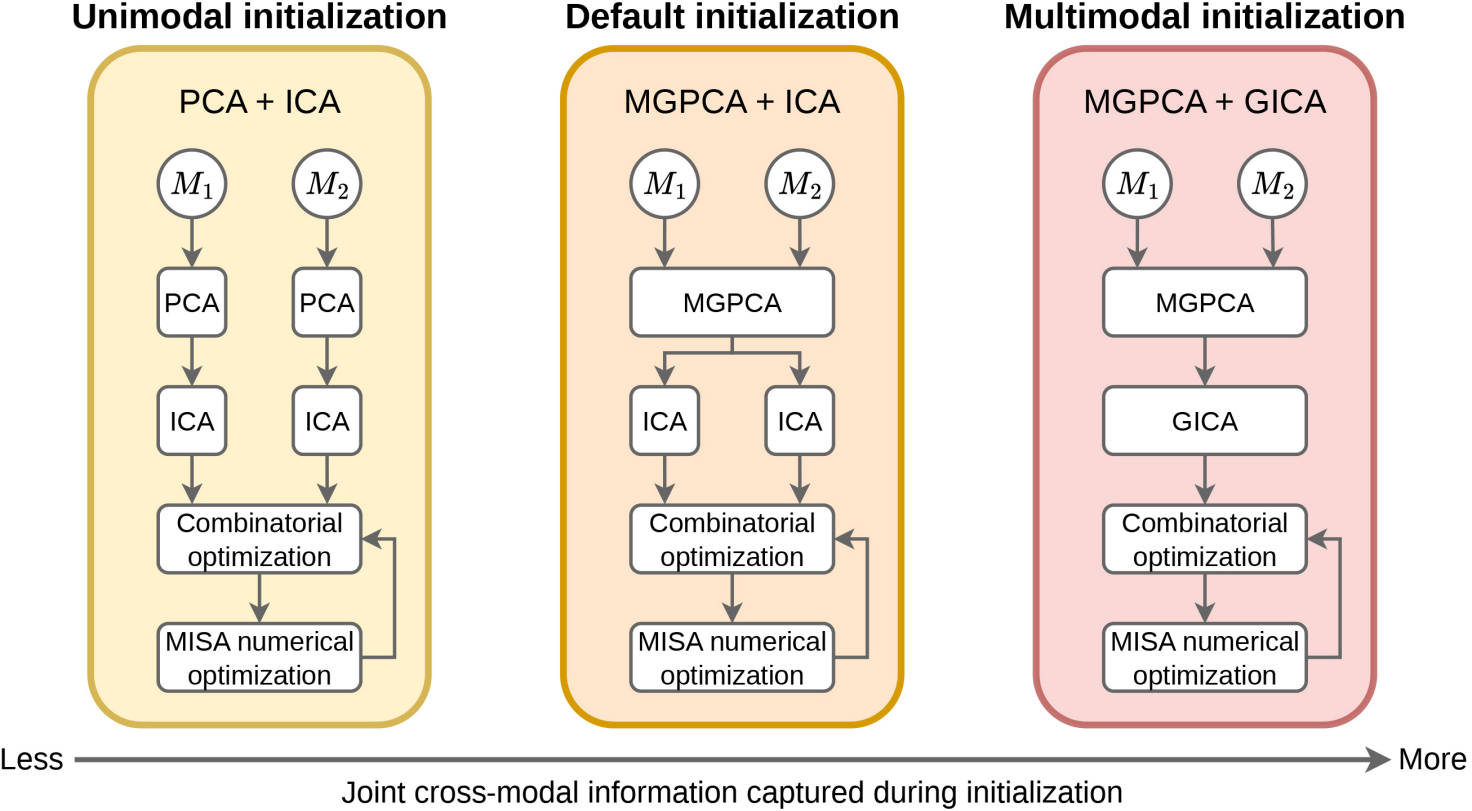
Overview of three proposed initialization workflows. Three initialization approaches are shown from left to right: (1) unimodal initialization: separate PCA and ICA per modality (PCA+ICA); (2) default initialization: multimodal group PCA with separate ICA per modality (MGPCA+ICA); and (3) multimodal initialization: multimodal group PCA with group ICA (MGPCA+GICA). Following initialization, greedy combinatorial optimization and numerical optimization with the MISA loss are performed alternately until convergence.

We refer to our proposed approach as Multimodal Subspace Independent Vector Analysis (MSIVA) because it extends MMIVA, linking equal-size, *high-dimensional* subspaces across modalities. We consider five candidate subspace structures that define different types of multimodal relationships (Fig. 1) and three initialization workflows that capture different levels of joint cross-modal information (Fig. 2). Given a candidate subspace structure, MSIVA consists of iterative combinatorial optimization of the source estimates (cross-modal subspace alignment) and numerical optimization of the MISA loss (Equation 6). This process is repeated for each of the five candidate subspace structures, followed by a best-fit determination based on the final quantitative metrics of all candidates.

#### 2.1.1 Designing candidate subspace structures

Higher-dimensional subspaces can capture richer latent relationships in neuroimaging data, including statistical dependence within and between modalities. In designing subspace structures, we consider two main objectives. First, unlike MMIVA, which assumes that all sources are statistically independent, our approach aims to identify *subspaces*—groups of linked (*not* independent) sources—within each modality, while assuming independence between subspaces. Second, we aim to detect statistical dependence between subspaces across different modalities, which involves solving a computationally intensive combinatorial optimization problem. To simplify this process, we restrict the search space by assuming that cross-modal dependence occurs only between equal-size, high-dimensional subspaces (containing two or more dimensions), ensuring direct source correspondence across modalities. Additionally, we treat all modality-specific subspaces as one-dimensional (1D ), each representing a single unimodal source.

Building on the MISA framework (Silva et al., 2021), we require user-defined candidate subspace structures that specify hypothesized linkage patterns. The goal of MSIVA is to determine which one of the candidate subspace structures best fits the observed data. We consider two questions when designing candidate subspace structures. First, *how many sources should be included?* We recommend estimating the model order of the real data using information-theoretic criteria (Y.-O. Li et al., 2007) and selecting the number of sources that capture sufficient information on all modalities. Second, *how should the sources be grouped?* In the functional imaging literature, two- to four-dimensional clusters have been used to hierarchically group sources (Ke et al., 2019; Ma et al., 2010, 2011). Accordingly, we define subspace structures with two- to four-dimensional cross-modal subspaces. We consider both homogeneous subspace structures, where all groups have equal dimension, and one heterogeneous structure, where groups have varying dimensions. We therefore propose five plausible subspace structures ($S_{1}-S_{5}$) for two modalities ($M_{1},M_{2}$), all with 12 sources in each modality (Fig. 1):

- $S_{1}$: One two-dimensional (2D ) cross-modal subspace, one three-dimensional (3D ) cross-modal subspace, one four-dimensional (4D ) cross-modal subspace, and three 1D unimodal subspaces.
- $S_{2}$: Five 2D cross-modal subspaces and two 1D unimodal subspaces.
- $S_{3}$: Three 3D cross-modal subspaces and three 1D unimodal subspaces.
- $S_{4}$: Two 4D cross-modal subspaces and four 1D unimodal subspaces.
- $S_{5}$: Twelve 1D cross-modal subspaces (no unimodal subspaces, identical to MMIVA).

#### 2.1.2 MSIVA default initialization workflow

Our previous studies demonstrate that proper initialization of unmixing weights can pre-align latent sources, leading to faster convergence to optimal solutions (X. Li, Khosravinezhad, et al., 2023; Silva et al., 2024). Based on these findings, we evaluated three initialization workflows incorporating different levels of joint cross-modal information.

The default initialization workflow utilizes multimodal group principal component analysis (MGPCA) to identify common principal components across all modalities and then applies ICA on the MGPCA-reduced data of each modality. Unlike standard principal component analysis (PCA), which identifies orthogonal directions of maximal variation for each modality separately, MGPCA identifies directions of maximal *common* variation across all modalities while ensuring equal variance contribution from each modality. The eigenvectors are computed based on the weighted average of the covariance matrices:

$$\Sigma_{avg}=\frac{1}{M}\sum_{m=1}^{M}N\frac{\Sigma^{\left[m\right]}}{trace(\Sigma^{\left[m\right]})}=\frac{1}{M}\sum_{m=1}^{M}N\frac{X^{{\left[m\right]}^{T}}X^{\left[m\right]}}{||X^{\left[m\right]}|{|}_{Fr}^{2}},$$ (3)

where $\Sigma^{\left[m\right]}=\frac{X^{[m]T}X^{\left[m\right]}}{V-1}∼E[X^{[m]T}X^{\left[m\right]}],\;\;\;\;\;E[\cdot ]$, E[⋅] is the expectation operator, and $||\cdot |{|}_{\text{Fr}}$ indicates the Frobenius norm. The scaling factor $\frac{\text{trace}\left(\Sigma^{\left[m\right]}\right)}{N}$ is the ratio of the variance in the data modality to the number of samples. We define the whitening matrix ${\text{W}}_{\text{MGPCA}}^{\left[m\right]}$ as follows:

$$W_{MGPCA}^{\left[m\right]}=\sqrt{N-1}\Lambda^{-\frac{1}{2}}U^{{\left[m\right]}^{T}}\kappa^{\left[m\right]},$$ (4)

where Λ and Q are the top C eigenvalues and eigenvectors of $\Sigma_{\text{avg}}$ , respectively, ${\text{U}}^{\left[m\right]}=\left(\kappa^{\left[m\right]}{\text{X}}^{\left[m\right]}\right)\text{Q}\Lambda^{-\frac{1}{2}}$, and $\kappa^{\left[m\right]}=\sqrt{\frac{N}{M\left(V-1\right)\text{trace}\left(\Sigma^{\left[m\right]}\right)}}=\sqrt{\frac{N}{M{\left\|{\text{X}}^{\left[m\right]}\right\|}_{\text{Fr}}^{2}}}$.

Next, the MGPCA-reduced data from each modality ${\text{X}}_{\text{r}}^{\left[m\right]}={\text{W}}_{\text{MGPCA}}^{\left[m\right]}{\text{X}}^{\left[m\right]}$ undergo a separate ICA estimation using the Infomax algorithm (Bell & Sejnowski, 1995) initialized with an identity matrix to obtain C independent sources per modality ${\hat{\text{S}}}_{\text{Infomax}}^{\left[m\right]}={\text{W}}_{\text{Infomax}}^{\left[m\right]}{\text{X}}_{\text{r}}^{\left[m\right]}$. These estimates are further optimized by running MISA as a unimodal ICA model initialized with ${\text{W}}_{\text{Infomax}}^{\left[m\right]}$, leading to the final unimodal ICA source estimates ${\hat{\text{S}}}_{\text{ICA}}^{\left[m\right]}={\text{W}}_{\text{ICA}}^{\left[m\right]}{\text{X}}_{\text{r}}^{\left[m\right]}$. Finally, MSIVA is initialized by the combined MGPCA and ICA estimates ${\text{W}}_{\text{0}}^{\left[m\right]}={\text{W}}_{\text{ICA}}^{\left[m\right]}{\text{W}}_{\text{MGPCA}}^{\left[m\right]}$. To evaluate the effect of cross-modal information during initialization, we additionally consider a fully unimodal initialization workflow and a fully multimodal initialization workflow.

#### 2.1.3 Unimodal initialization workflow

The unimodal initialization workflow applies PCA and ICA on each modality separately. The data matrix from each modality ${\text{X}}^{\left[m\right]}$ is projected into a reduced data matrix ${\text{X}}_{\text{r}}^{\left[m\right]}$ with C principal components, yielding the corresponding whitening matrix ${\text{W}}_{\text{PCA}}^{\left[m\right]}$. ICA is then applied to each reduced data matrix ${\text{X}}_{\text{r}}^{\left[m\right]}$ to obtain C independent sources and the corresponding unmixing matrix ${\text{W}}_{\text{ICA}}^{\left[m\right]}$. In this workflow, the MISA initialization matrix is defined as ${\text{W}}_{\text{0}}^{\left[m\right]}={\text{W}}_{\text{ICA}}^{\left[m\right]}{\text{W}}_{\text{PCA}}^{\left[m\right]}$.

#### 2.1.4 Multimodal initialization workflow

The multimodal initialization workflow sequentially applies MGPCA (as defined in Equation 4) and group ICA (GICA) across all data modalities, resulting in the weight matrices ${\text{W}}_{\text{MGPCA}}^{\left[m\right]}$ and ${\text{W}}_{\text{GICA}}^{\left[m\right]}$. GICA performs ICA on the combined MGPCA-reduced data from all M modalities, that is, ${\text{X}}_{\text{r}}=\sum_{m=1}^{M}{\text{X}}_{\text{r}}^{\left[m\right]}$. In this workflow, MISA is initialized by ${\text{W}}_{\text{0}}^{\left[m\right]}={\text{W}}_{\text{GICA}}^{\left[m\right]}{\text{W}}_{\text{MGPCA}}^{\left[m\right]}$.

#### 2.1.5 Alternating combinatorial and numerical optimization

All three workflows utilize MISA’s greedy combinatorial optimization and objective function to estimate latent sources. MISA employs the L-BFGS algorithm (Liu & Nocedal, 1989) within a barrier-type numerical optimizer (fmincon from MATLAB Optimization Toolbox), using a relative gradient update rule.^2^ The MISA loss function ${ℒ}_{\text{MISA}}\left(\cdot \right)$ (Silva et al., 2021) is defined as the Kullback-Leibler (KL) divergence between the joint distribution of all recovered sources $p\left(\hat{\text{S}}\right)$ and the product of all K subspace distributions $q\left(\hat{\text{S}}\right)=\prod_{k=1}^{K}p\left({\hat{\text{S}}}_{k}\right)$, which is equivalent to mutual information among K subspaces. Thus, subspaces within each modality are assumed to be statistically independent of one another, while sources within each subspace are considered statistically dependent. The source distribution for subspace k is modeled as a Kotz distribution (Kotz, 1975; Nadarajah, 2003):

$$p\left({\hat{\text{S}}}_{k}\right)=\frac{\beta_{k}\lambda_{k}^{\nu_{k}}\Gamma \left(\frac{d_{k}}{2}\right){\left({\hat{\text{S}}}_{k}^{⊤}{\text{D}}_{k}^{-1}{\hat{\text{S}}}_{k}\right)}^{\eta_{k}-1}}{\pi^{\frac{d_{k}}{2}}{\left(\text{det}\left({\text{D}}_{k}\right)\right)}^{\frac{1}{2}}\Gamma \left(\nu_{k}\right)}e^{-\lambda_{k}{\left({\hat{\text{S}}}_{k}^{⊤}{\text{D}}_{k}^{-1}{\hat{\text{S}}}_{k}\right)}^{\beta_{k}}},$$ (5)

where ${\hat{\text{S}}}_{k}=\left[{\hat{\text{S}}}_{k}^{\left[1\right]};\ldots ;{\hat{\text{S}}}_{k}^{\left[M\right]}\right]\in {\mathbb{R}}^{d_{k}\times N}$ denotes the estimated sources in cross-modal subspace k across M modalities,^3^ while ${\hat{\text{ S}}}_{k}={\hat{\text{S}}}_{k}^{\left[m\right]}\in {\mathbb{R}}^{d_{k}\times N}$ denotes the estimated source in unimodal subspace k. Here, $d_{k}$ is the dimensionality of subspace k: for cross-modal subspaces, $d_{k}=M\cdot C_{k}$, where $C_{k}$ is the number of sources per modality in subspace k, which is assumed equal across modalities; for unimodal subspaces, $d_{k}=1$ . $\beta_{k}>0$ , $\lambda_{k}>0$ , and $\eta_{k}>\frac{2-d_{k}}{2}$ are the Kotz parameters that control the shape of the distribution, the scale, and the density near the origin, respectively. For brevity, we define $\nu_{k}≜\frac{2\eta_{k}+d_{k}-2}{2\beta_{k}}>0$ and $\alpha_{k}≜\frac{\Gamma \left(\nu_{k}+\beta_{k}^{-1}\right)}{\lambda_{k}^{\beta_{k}^{-1}}d_{k}\Gamma \left(\nu_{k}\right)}$. Γ(⋅) denotes the gamma function. det(⋅) denotes the determinant. The positive definite dispersion matrix ${\text{D}}_{k}$ is given by ${\text{D}}_{k}=\alpha_{k}^{-1}\Sigma_{k}$, where $\Sigma_{k}$ is the source *covariance* matrix for subspace k. In MSIVA, ${\text{D}}_{k}$ is iteratively reset as the current estimate of the source *correlation* matrix for subspace k. The Kotz parameters are set to $\beta_{k}=0.5$ and $\eta_{k}=1$ , producing a Laplace distribution, and $\lambda_{k}$ is automatically adjusted in each subspace to fix the source variance $\alpha_{k}=\frac{\pi^{2}}{3}$, corresponding to the variance of the standard logistic distribution used in Infomax.

We want to minimize the loss function ${ℒ}_{\text{MISA}}\left(\cdot \right)$ by solving the following optimization problem:

$$\begin{array}{cc} \begin{array}{cc} \text{min}{ℒ}_{\text{MISA}}\left(\hat{\text{S}}\right) & =\text{min}D_{\text{KL}}\left(p\left(\hat{\text{S}}\right)∥q\left(\hat{\text{S}}\right)\right)\\ & =\text{min}D_{\text{KL}}\left(p\left(\hat{\text{S}}\right)∥\prod_{k=1}^{K}p\left({\hat{\text{S}}}_{k}\right)\right)\\ & =\text{min}E\left[\text{ln}p\left(\hat{\text{S}}\right)\right]-\sum_{k=1}^{K}E\left[\text{ln}p\left({\hat{\text{S}}}_{k}\right)\right]\\ & =\underset{\begin{array}{c} \hat{\text{W}},{\text{P}}_{k},\\ k=1,\ldots ,K \end{array}}{\text{ min}\;\;E}\left[\text{ln}p\left(\hat{\text{W}}\text{X}\right)\right]-\sum_{k=1}^{K}E\left[\text{ln}p\left({\text{P}}_{k}\hat{\text{W}}\text{X}\right)\right], \end{array} & \end{array}$$ (6)

where $\hat{\text{S}}=\left[{\hat{\text{S}}}^{\left[1\right]};\ldots ;{\hat{\text{S}}}^{\left[M\right]}\right]\in {\mathbb{R}}^{MC\times N}$ represents the estimated sources across all M modalities. $\text{X}=\left[{\text{X}}^{\left[1\right]};\ldots ;{\text{X}}^{\left[M\right]}\right]\in {\mathbb{R}}^{MV\times N}$ represents the concatenated data from all M modalities. $\hat{\text{W}}\in {\mathbb{R}}^{MC\times MV}$ is the estimated block-diagonal unmixing matrix, such that ${\hat{\text{S}}}^{\left[m\right]}={\hat{\text{W}}}^{\left[m\right]}{\text{X}}^{\left[m\right]}$. ${\text{P}}_{k}\;\in {\mathbb{R}}^{d_{k}\times MC}$ is the k-th subspace assignment matrix defined by the subspace structure S in Section 2.1.1.

We alternated between greedy combinatorial optimization and numerical optimization until the loss value converged. We performed 10 rounds of alternating combinatorial and numerical optimization for synthetic data and 20 for neuroimaging data. During each combinatorial optimization, the rows of the modality-specific unmixing matrix ${\hat{\text{W}}}^{\left[m\right]}$ were permuted to escape potential local minima found by the numerical optimizer. The numerical optimizer then resumed for 150 iterations, after which combinatorial optimization resumed. Finally, we selected the unmixing matrix ${\hat{\text{W}}}^{\left[m\right]}$ corresponding to the lowest loss value across all iterations.

### 2.2 Datasets

#### 2.2.1 Synthetic data

For each subspace structure S, we generated a synthetic dataset with two modalities $\text{X}=\left[{\text{X}}^{\left[1\right]};{\text{X}}^{\left[2\right]}\right]\in {\mathbb{R}}^{2V\times N}$ , where V is the number of features (V=20000 ) and N is the number of samples (N=3000 ). V is a sufficiently large number for covariance estimation in PCA and MGPCA. N was chosen to approximate the number of samples in the UK Biobank (UKB) neuroimaging dataset in this study (see Section 2.2.2). Each data modality was modeled as a linear mixture of C=12 sources spanning the subspaces defined in S, ${\text{X}}^{\left[m\right]}={\text{A}}^{\left[m\right]}{\text{S}}^{\left[m\right]}$, ${\text{A}}^{\left[m\right]}\in {\mathbb{R}}^{V\times C}$ , ${\text{S}}^{\left[m\right]}\in {\mathbb{R}}^{C\times N}$ , m∈{1,2}. Each cross-modal subspace ${\text{S}}_{k}$ was sampled independently from a multivariate Laplace distribution, and therefore, marginal distributions correspond to individual sources. Within each cross-modal subspace, sources were statistically dependent, with correlation coefficients uniformly sampled from [0.65,0.85]. Unimodal sources (1D subspaces in $S_{1}-S_{4}$) also followed Laplace distributions but were uncorrelated with all other sources and subspaces.

#### 2.2.2 Neuroimaging data

We utilized two large multimodal neuroimaging datasets, each including T1-weighted structural MRI (sMRI) and resting-state functional MRI (fMRI). The first dataset was a subset of the UK Biobank study (Miller et al., 2016). After excluding subjects with more than 4% missing phenotype measures (Smith et al., 2015), 2907 subjects from two sites were used for formal analysis (age mean ± standard deviation: 62.09±7.32 years; age median: 63 years; age range: 46−79 years; 1452 males, 1455 females). The second dataset combined 999 participants (age mean ± standard deviation: 38.61±13.13 years; age median: 39 years; age range: 15−65 years; 625 males, 374 females) from four studies: the Bipolar and Schizophrenia Network for Intermediate Phenotypes (BSNIP) (Tamminga et al., 2014), the Center for Biomedical Research Excellence (COBRE) (Aine et al., 2017), the Function Biomedical Informatics Research Network (FBIRN) (Keator et al., 2016), and the Maryland Psychiatric Research Center (MPRC). Participants included 538 controls, 337 patients diagnosed with schizophrenia, 63 patients with bipolar disorder, 11 patients with schizoaffective disorder, 28 schizoaffective bipolar-type probands, and 22 schizoaffective depression-type probands.

For each dataset, we preprocessed sMRI and fMRI to obtain gray matter (GM) and mean-scaled amplitude of low-frequency fluctuations (mALFF) feature maps, respectively. We resampled each GM or mALFF feature map to $3\times 3\times 3\;{\text{mm}}^{3}$ resolution and applied a group-level GM mask to each feature map, resulting in 44318 voxels (for acquisition and preprocessing details, see Supplementary Material, Section 1). Next, for each data modality in each dataset, we performed variance normalization for each subject (zero-mean, unit-variance across voxels), then removed the mean across all subjects for each voxel. Lastly, we regressed out site effects for each dataset as follows:

$${\text{X}}^{\left[m\right]}\leftarrow {\text{X}}^{\left[m\right]}-{\text{X}}^{\left[m\right]}\text{L}{\left({\text{L}}^{⊤}\text{L}\right)}^{-1}{\text{L}}^{⊤},$$ (7)

where **L** = [**1**,  **ℓ**], with $\text{1}\in {\mathbb{R}}^{N}$ being a column vector of ones and **ℓ** being one-hot encoded site labels.

### 2.3 Quantitative evaluation metrics

#### 2.3.1 Normalized multidataset Moreau-Amari intersymbol interference

The normalized multidataset Moreau-Amari intersymbol interference (MISI) (Amari et al., 1995; Charkani & Deville, 1999; Silva et al., 2021) was used to evaluate the residual interference between the estimated unmixing matrix $\hat{\text{W}}$ and the ground-truth mixing matrix A:

$$\begin{array}{l} \text{MISI}\left(\text{H}\right)=\frac{1}{2K\left(K-1\right)}[\sum_{i=1}^{K}\left(-1+\sum_{j=1}^{K}\frac{\left|h_{ij}\right|}{\underset{k}{\text{max}}\left|h_{ik}\right|}\right)\\ \;\;\;\;\;\;\;\;\;\;\;\;\;\;\;+\sum_{j=1}^{K}\left(-1+\sum_{i=1}^{K}\frac{\left|h_{ij}\right|}{\underset{k}{\text{max}}\left|h_{kj}\right|}\right)], \end{array}$$ (8)

where $h_{ij}={\text{1}}^{⊤}\left|{\text{P}}_{i}\hat{\text{W}}\text{A}{\text{P}}_{j}\right|\text{1}$ is the sum of absolute values from all elements corresponding to subspaces i and j in the interference matrix $\text{H}=\hat{\text{W}}\text{A}$ .

#### 2.3.2 MISA loss

The MISA loss defined in Equation (6) was reported for all experiments. When evaluating performance on synthetic data, the MISI metric and the interference matrix H were prioritized because they leverage ground-truth information. In addition, we examined whether the loss values were consistent with the MISI values on synthetic data.

#### 2.3.3 Mean correlation coefficient and minimum distance

Two metrics were used to summarize cross-modal source correlations: the mean correlation coefficient (MCC) and the minimum distance (MD). Both metrics were computed in a two-stage manner. First, we constructed an aggregated correlation matrix across cross-modal subspaces. Then, the MCC and MD were derived from the aggregated matrix. This approach ensures balanced contributions from subspaces with varying dimensions. *Multimodal* MCC (MMCC) and *multimodal* MD (MMD) measure correlations between recovered and ground-truth sources within each modality, followed by min–max subspace aggregation across modalities. *Cross-modal* MCC (CMCC) and *cross-modal* MD (CMD) measure correlations between recovered sources across two modalities, without reference to ground truth.

The computation of these metrics involved three distinct correlation matrices: (1) absolute correlations between recovered sources and ground-truth sources from the first modality ${\text{R}}^{\left[1\right]}\in {\mathbb{R}}^{C\times C}$ ; (2) absolute correlations between recovered sources and ground-truth sources from the second modality ${\text{R}}^{\left[2\right]}\in {\mathbb{R}}^{C\times C}$ ; (3) absolute cross-modal correlations between recovered sources across the two modalities ${\text{R}}^{\text{cm}}\in {\mathbb{R}}^{C\times C}$ . The correlation matrix of the first modality ${\text{R}}^{\left[1\right]}$ was sorted into a block-diagonal structure consistent with the predefined subspace structure, and the same permutation was then applied to the correlation matrix of the second modality ${\text{R}}^{\left[2\right]}$. Since the cross-modal correlation matrix ${\text{R}}^{\text{cm}}$ was computed without reference to ground-truth sources, it was left unsorted to preserve the original linkage between recovered sources across modalities. In the following equations, δ(condition) denotes an indicator function equal to 1 when the specified condition is satisfied and 0 otherwise.

**Multimodal mean correlation coefficient.** Given a modality-specific correlation matrix ${\text{R}}^{\left[m\right]}$, the subspace-aggregated correlation matrix ${\bar{\text{R}}}^{\left[m\right]}$ summarizes the correlation between each pair of cross-modal subspaces i and j:

$${\bar{\text{R}}}_{\left[i,j\right]}^{\left[m\right]}=\frac{1}{C_{i}+C_{j}}\left({\text{1}}^{⊤}\cdot \text{rowmax}\left({\text{R}}_{ij}^{\left[m\right]}\right)+\text{columnmax}\left({\text{R}}_{ij}^{\left[m\right]}\right)\cdot \text{1}\right),$$ (9)

where ${\bar{\text{R}}}_{\left[i,j\right]}^{\left[m\right]}$ is the element at row i and column j in ${\bar{\text{R}}}^{\left[m\right]}\in {\mathbb{R}}^{K_{\text{c}}\times K_{\text{c}}}$, with $K_{\text{c}}$ being the number of cross-modal subspaces and m∈{1,2} indexing the modality. ${\text{R}}_{ij}^{\left[m\right]}\in {\mathbb{R}}^{C_{i}\times C_{j}}$ denotes the correlation block between cross-modal subspaces i and j in ${\text{R}}^{\left[m\right]}$, and $C_{i}$ and $C_{j}$ are the numbers of sources per modality in cross-modal subspaces i and j, respectively. 1 denotes a column vector of ones. $\text{rowmax}\left({\text{R}}_{ij}^{\left[m\right]}\right)$ denotes a column vector containing the maximum value of each row in ${\text{R}}_{ij}^{\left[m\right]}$, and $\text{columnmax}\left({\text{R}}_{ij}^{\left[m\right]}\right)$ denotes a row vector containing the maximum value of each column in ${\text{R}}_{ij}^{\left[m\right]}$.

The multimodal correlation matrix ${\bar{\text{R}}}^{\text{mm}}$ was then calculated by taking the element-wise minimum for the diagonal elements and the element-wise maximum for the off-diagonal elements between the two aggregated modality-specific correlation matrices (${\bar{\text{R}}}^{\left[1\right]}$ and ${\bar{\text{R}}}^{\left[2\right]}$):

$${\bar{\text{R}}}_{\left[i,j\right]}^{\text{mm}}=\text{max}\left({\bar{\text{R}}}_{\left[i,j\right]}^{\left[1\right]},{\bar{\text{R}}}_{\left[i,j\right]}^{\left[2\right]}\right)\cdot \delta \left(i\neq j\right)+\text{min}\left({\bar{\text{R}}}_{\left[i,j\right]}^{\left[1\right]},{\bar{\text{R}}}_{\left[i,j\right]}^{\left[2\right]}\right)\cdot \delta \left(i=j\right).$$ (10)

The multimodal mean correlation coefficient (MMCC) was computed by averaging the diagonal elements of ${\bar{\text{R}}}^{\text{mm}}$ :

$$\text{MMCC}\left({\bar{\text{R}}}^{\text{mm}}\right)=\frac{1}{K}\sum_{k=1}^{K}{\bar{\text{R}}}_{\left[k,k\right]}^{\text{mm}}.$$ (11)

**Cross-modal mean correlation coefficient.** The aggregated cross-modal correlation matrix ${\bar{\text{R}}}^{\text{cm}}$ was calculated in a similar manner:

$${\bar{\text{R}}}_{\left[i,j\right]}^{\text{cm}}=\frac{1}{C_{i}+C_{j}}\left({\text{1}}^{⊤}\cdot \text{rowmax }\left({\text{R}}_{ij}^{\text{cm}}\right)+\text{columnmax }\left({\text{R}}_{ij}^{\text{cm}}\right)\cdot \text{1}\right),$$ (12)

where ${\bar{\text{R}}}_{\left[i,j\right]}^{\text{cm}}$ is the element at row i and column j in ${\bar{\text{R}}}^{\text{cm}}\in {\mathbb{R}}^{K_{\text{c}}\times K_{\text{c}}}$, and ${\text{R}}_{ij}^{\text{cm}}\in {\mathbb{R}}^{C_{i}\times C_{j}}$ denotes the correlation block between cross-modal subspaces i and j in ${\text{R}}^{\text{cm}}$ .

The cross-modal mean correlation coefficient (CMCC) was then computed by averaging the diagonal elements of ${\bar{\text{R}}}^{\text{cm}}$ :

$$\text{CMCC }\left({\bar{\text{R}}}^{\text{cm}}\right)=\frac{1}{K}\sum_{k=1}^{K}{\bar{\text{R}}}_{\left[k,k\right]}^{\text{cm}}.$$ (13)

**Multimodal and cross-modal minimum distance.** The multimodal minimum distance (MMD) and the cross-modal minimum distance (CMD) were computed by aggregating *all* (diagonal and off-diagonal) elements of ${\bar{\text{R}}}^{\text{mm}}$ and ${\bar{\text{R}}}^{\text{cm}}$ , respectively. The diagonal elements capture the correlation between sources within the same subspace, while the off-diagonal elements capture the correlation between sources from different subspaces. The MMD and CMD thus jointly measure both within-subspace alignment and between-subspace separation.

$$\begin{array}{l} \text{MMD}\left({\bar{\text{R}}}^{\text{mm}}\right)\\ \;=\frac{1}{K^{2}}\sum_{i=1}^{K}\sum_{j=1}^{K}\left({\bar{\text{R}}}_{\left[i,j\right]}^{\text{mm}}\cdot \delta \left(i\neq j\right)+\left(1-{\bar{\text{R}}}_{\left[i,j\right]}^{\text{mm}}\right)\cdot \delta \left(i=j\right)\right), \end{array}$$ (14)

$$\text{CMD}\left({\bar{\text{R}}}^{\text{cm}}\right)=\frac{1}{K^{2}}\sum_{i=1}^{K}\sum_{j=1}^{K}\left({\bar{\text{R}}}_{\left[i,j\right]}^{\text{cm}}\cdot \delta \left(i\neq j\right)+\left(1-{\bar{\text{R}}}_{\left[i,j\right]}^{\text{cm}}\right)\cdot \delta \left(i=j\right)\right).$$ (15)

### 2.4 Experiments

#### 2.4.1 Synthetic data experiment

To verify whether MSIVA could identify and distinguish the correct subspace structure (the one used for data generation) from the incorrect ones, we generated a synthetic dataset for each of the five subspace structures ($S_{1}-S_{5}$; Section 2.1.1), where the data distribution was defined by the corresponding subspace structure (Section 2.2.1). Next, we conducted experiments on all combinations of the five subspace structures (Fig. 1) and three initialization workflows (Fig. 2). Finally, the absolute values of the interference matrices ${\text{H}}^{\left[m\right]}={\hat{\text{W}}}^{\left[m\right]}{\text{A}}^{\left[m\right]}$^4^ were examined to verify whether the ground-truth subspace structures were recovered, and the MISI and final MISA loss values were reported.

#### 2.4.2 Neuroimaging data experiment

We performed experiments on each of the two multimodal neuroimaging datasets separately, using all combinations of the five candidate subspace structures and three initialization workflows. The optimal subspace structure and initialization workflow were identified as those yielding the lowest final MISA loss value. Unlike the synthetic data experiments, the MISI metric is not available because the ground-truth subspace structure is unknown for real data. Instead, we measured cross-modal subspace alignment using the cross-modal metrics (CMCC and CMD). Cross-modal source correlations were computed using both the *linear* Pearson correlation coefficient and the *nonlinear* randomized dependence coefficient (RDC) (Lopez-Paz et al., 2013).

To further assess the cross-modal linkage strength *within* each estimated subspace of the optimal subspace structure, post-hoc CCA was applied separately to each cross-modal subspace. For cross-modal subspace k, CCA finds projection vectors ${\hat{\text{p}}}_{k}$ and ${\hat{\text{q}}}_{k}$ that maximize the cross-modal correlation between two sets of sources:

$$\left({\hat{\text{p}}}_{k},{\hat{\text{q}}}_{k}\right)=\underset{{\text{p}}_{k},{\text{q}}_{k}}{\text{argmax}}\text{corr }\left({\text{p}}_{k}^{⊤}{\hat{\text{S}}}_{k}^{\left[1\right]},{\text{q}}_{k}^{⊤}{\hat{\text{S}}}_{k}^{\left[2\right]}\right),$$ (16)

where ${\text{p}}_{k}\in {\mathbb{R}}^{C_{k}}$ and ${\text{q}}_{k}\;\in {\mathbb{R}}^{C_{k}}$ are the CCA projection vectors, and ${\hat{\text{S}}}_{k}^{\left[1\right]}\in {\mathbb{R}}^{C_{k}\times N}$ and ${\hat{\text{S}}}_{k}^{\left[2\right]}\in {\mathbb{R}}^{C_{k}\times N}$ are the recovered sources for cross-modal subspace k. The post-CCA sources are then given by ${\hat{\text{p}}}_{k}^{⊤}{\hat{\text{S}}}_{k}^{\left[1\right]}$ and ${\hat{\text{q}}}_{k}^{⊤}{\hat{\text{S}}}_{k}^{\left[2\right]}$. This assessment is valid because linear transformations of individual sources within the same subspace are considered equivalently optimal^5^ (Cardoso, 1998; Szabó et al., 2012).

### 2.5 Brain-phenotype prediction

To evaluate associations between phenotype measures and cross-modal post-CCA sources, we conducted age prediction and sex classification on the UKB dataset, and age prediction and binary diagnosis classification (controls versus patients with SZ) on the patient dataset. Specifically, we used ridge regression for age prediction and linear-kernel support vector machine (SVM) for sex and diagnosis classification. For the UKB dataset, 2907 subjects were stratified into a training set of 2000 subjects and a holdout test set of 907 subjects. For the patient dataset, 999 subjects were stratified into a training set of 699 subjects and a holdout test set of 300 subjects for age prediction; 538 controls and 337 patients with SZ were grouped into a training set of 612 subjects and a test set of 263 subjects for diagnosis classification. In all cases, we performed 10 -fold cross-validation on the training set to select the optimal regularization parameter (search grid: {0.1,0.2,…,1}). The model was then retrained on all training subjects and evaluated on the holdout set. Age regression performance was measured by mean absolute error (MAE) between predicted and chronological age. Sex and diagnosis classification performance was assessed by *balanced* accuracy, defined as 0.5× (true positive rate + true negative rate).

### 2.6 Brain-age delta analysis on UK Biobank data

A key benefit of MSIVA is that the estimated multimodal sources are more expressive, as they leverage higher-dimensional (≥2D ) cross-modal subspaces. To demonstrate the utility of these subspaces, we conducted a two-stage *voxelwise* brain-age delta analysis using the estimated sources under the optimal subspace structure in the UKB data (Smith et al., 2019, 2020). For each voxel in the reconstructed data (${\hat{\text{X}}}_{k}^{\left[m\right]}={\hat{\text{A}}}_{k}^{\left[m\right]}{\hat{\text{S}}}_{k}^{\left[m\right]}$^6^), we estimated an initial brain-age delta in the first stage:

$$\delta_{1}={\hat{\text{X}}}_{v}\beta_{1}-\text{y},$$ (17)

where ${\hat{\text{X}}}_{v}$ denotes the predictor matrix for voxel v, including *shared* reconstructed features from each cross-modal subspace,^7^ reconstructed sMRI features from each cross-modal subspace, and reconstructed data from each unimodal subspace (for more details, see Supplementary Material, Section 2). $\text{y}\in {\mathbb{R}}^{N}$ is demeaned chronological age. The regression coefficient was estimated via ordinary least squares: $\beta_{1}={\left({\hat{\text{X}}}_{v}^{⊤}{\hat{\text{X}}}_{v}\right)}^{-1}{\hat{\text{X}}}_{v}^{⊤}\text{y}$ .

In the second stage, we decomposed $\delta_{1}$ into predictor-specific contributions while removing age dependence and other confounding factors. For each predictor i, we computed:

$$\delta_{2i}={\hat{\text{X}}}_{v\left[:,i\right]}\beta_{1i}-\text{Y}\beta_{2i},$$ (18)

where ${\hat{\text{X}}}_{v\left[:,i\right]}\beta_{1i}$ is the contribution of predictor i to $\delta_{1}$, $\text{Y}\in {\mathbb{R}}^{N\times 10}$ is a matrix of confounding variables (the demeaned linear, quadratic, and cubic age terms; sex; the interaction between sex and each of the three age terms; framewise displacement; and two spatial normalization variables), and $\beta_{2i}={\left({\text{Y}}^{⊤}\text{Y}\right)}^{-1}{\text{Y}}^{⊤}{\hat{\text{X}}}_{v\left[:,i\right]}\beta_{1i}$ . We then partialled each predictor-specific brain-age delta vector $\delta_{2i}$ against all other vectors $\delta_{2j}$ (j≠i) and concatenated all partialled brain-age delta vectors into the partialled brain-age delta matrix $\delta_{2p}$ .

To investigate multimodal brain-phenotype relationships, we correlated the voxelwise brain-age delta $\delta_{2p}$ with 25 non-imaging phenotype variables, including lifestyle factors and cognitive test scores (Supplementary Material, Section 3, Table S1). This voxelwise brain-age delta analysis allows us to visualize a voxel-level spatial map characterizing the relationship between each phenotype variable and the difference between chronological age and estimated brain age.

## 3 Results

### 3.1 MSIVA identifies ground-truth subspace structures in synthetic data

**Fig. 3.**
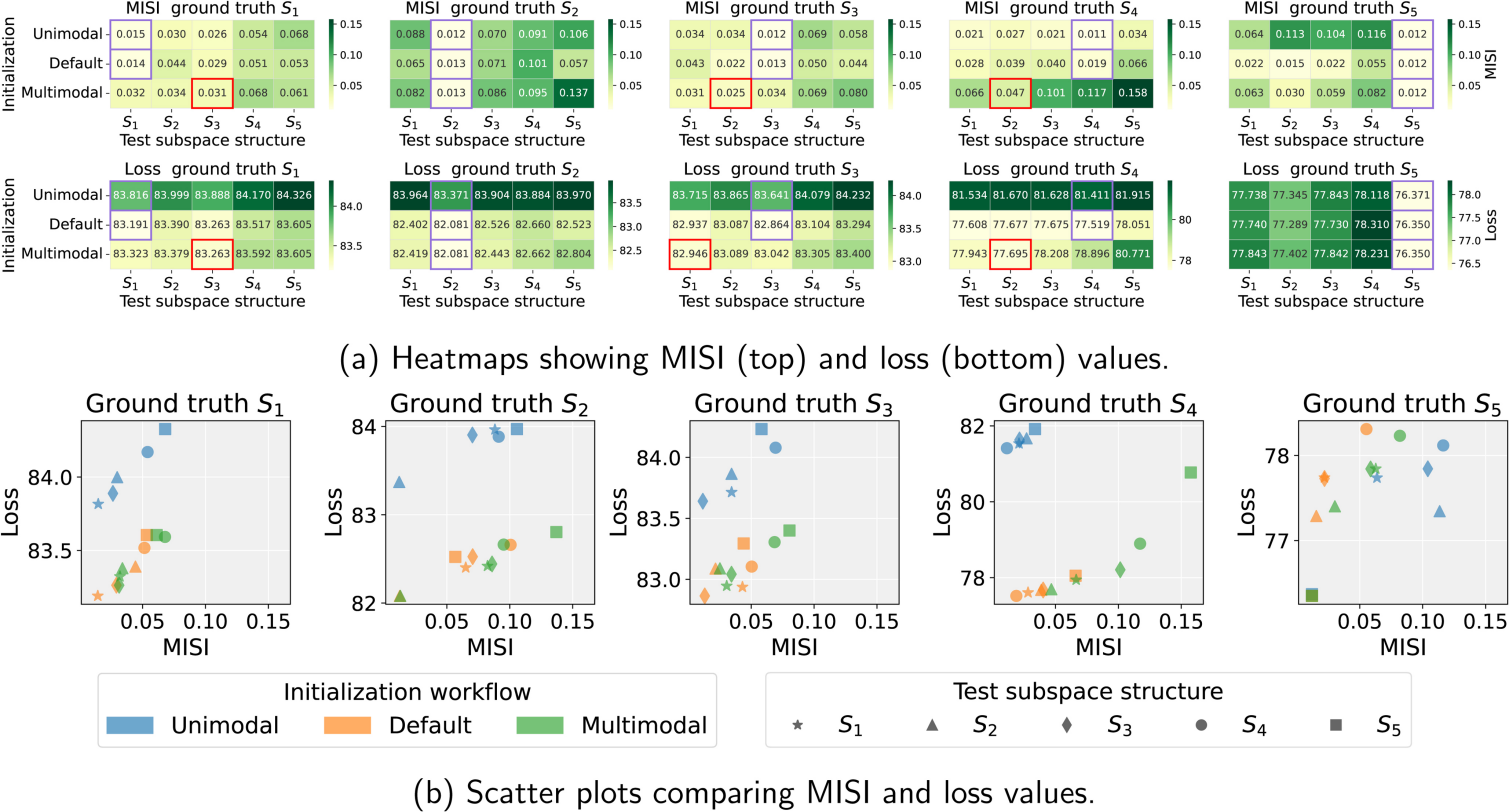
MISI and loss values on synthetic data. (a) Heatmaps showing MISI (top, lower is better) and loss (bottom, lower is better) values. Each panel represents a ground-truth subspace structure ($S_{1}-S_{5}$) used to generate the synthetic data. In each panel, each row represents an initialization workflow, and each column represents a test subspace structure used to fit the model. The best-performing test subspace structure per row is highlighted in a purple box if it matches the ground truth (matched) or a red box if it does not (mismatched). MISI ≤0.1 is considered a heuristic threshold for good source separation (Silva et al., 2014). Both the unimodal (PCA+ICA) and default (MGPCA+ICA) initialization workflows correctly identified all ground-truth subspace structures, whereas the multimodal initialization workflow (MGPCA+GICA) failed to detect subspace structures $S_{1}$, $S_{3}$, and $S_{4}$. Therefore, the unimodal and default initialization workflows more reliably recovered the ground-truth subspace structures than the multimodal initialization workflow. (b) Scatter plots comparing MISI and loss values. Each panel represents a ground-truth subspace structure ($S_{1}-S_{5}$). In each panel, loss values are closely aligned with MISI values within each initialization workflow, suggesting that loss serves as a valid proxy for MISI under the same initialization.

We verified whether MSIVA, including three different initialization workflows, could identify the correct subspace structures used to generate synthetic datasets. Following the initialization stage, the MSIVA default initialization method (MGPCA+ICA) achieved a well-balanced combination of unimodal separation and cross-modal alignment in most cases (Supplementary Material, Section 4, Fig. S2). Subsequently, loss values successfully converged within each numerical optimization run (Supplementary Material, Section 5, Fig. S3). After iterative combinatorial and numerical optimization, both the unimodal (PCA+ICA) and default (MGPCA+ICA) initialization workflows yielded the lowest MISI and loss values when the test subspace structure matched the ground truth (Fig. 3a), demonstrating that both approaches correctly recovered the ground-truth subspace structure. The multimodal initialization workflow (MGPCA+GICA), on the other hand, showed suboptimal performance when the ground-truth subspace structures contained 3D or 4D subspaces ($S_{1}$, $S_{3}$, and $S_{4}$). The final loss values were closely aligned with the MISI values when using the same initialization workflow (Fig. 3b), suggesting that loss serves as a valid proxy for MISI when ground-truth information is unavailable. A broader comparison against other metrics (MMCC, CMCC, MMD, CMD) confirmed that loss was the only metric consistently associated with MISI in the absence of ground-truth information (Supplementary Material, Section 5, Fig. S4).

**Fig. 4.**
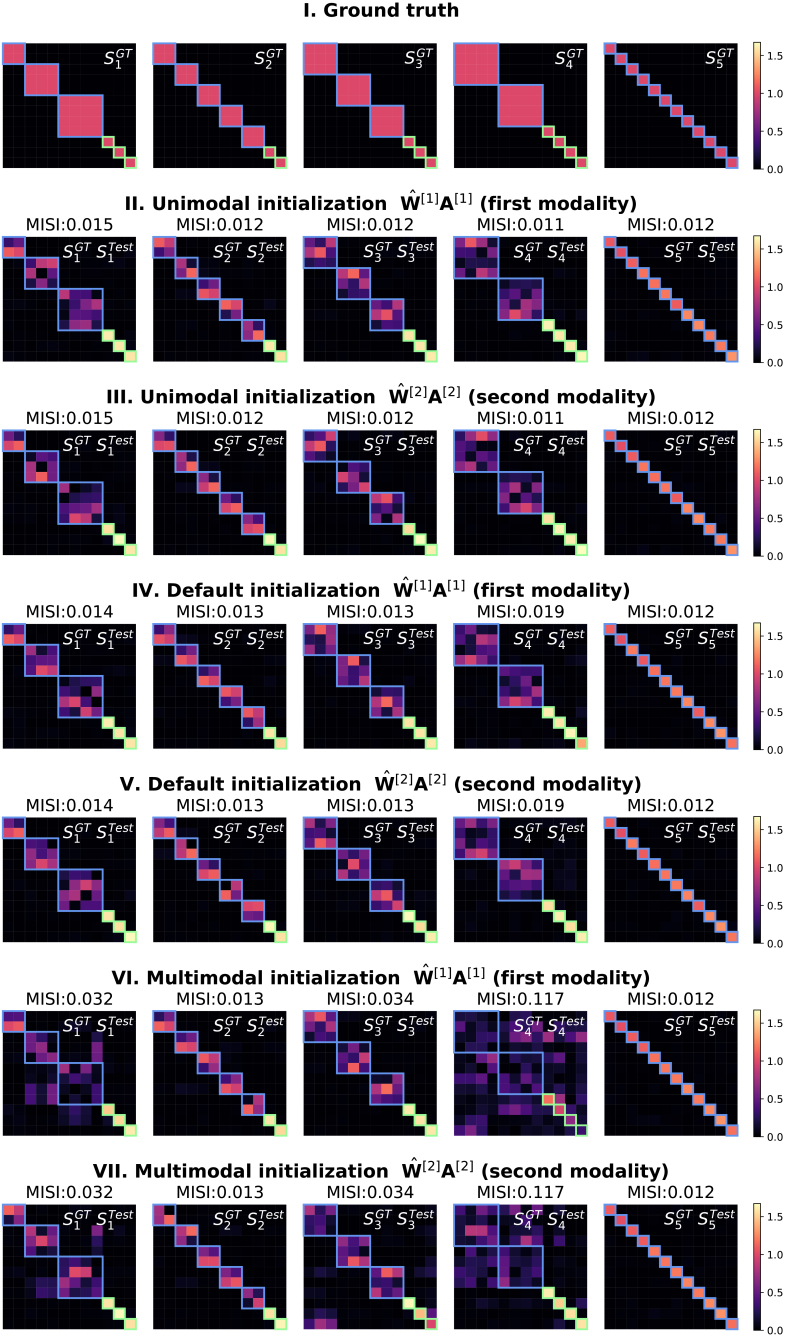
Interference matrices ${\text{H}}^{\left[m\right]}\;={\hat{\text{W}}}^{\left[m\right]}{\text{A}}^{\left[m\right]}$ when the test subspace structure matched the ground-truth subspace structure on synthetic data. Cross-modal subspaces are highlighted in blue and modality-specific subspaces in green. Each workflow occupies two rows (one per modality), and the same subspace permutation (reordering of subspaces for ease of interpretation) was applied to both modalities. The unimodal and default initialization workflows (rows II–V) correctly identified and aligned the subspace structures across both modalities, consistent with the ground-truth simulation design (row I). However, the multimodal initialization workflow (rows VI–VII) failed to fully recover $S_{1}$, $S_{3}$, and $S_{4}$.

As presented in Figure 4, the recovered subspace structures from the unimodal and default initialization workflows (rows II–V) under the correct subspace structure aligned well with the ground truth (row I), demonstrating the effectiveness of MSIVA. However, the multimodal initialization workflow (rows VI–VII) failed to fully recover the ground-truth subspace structures $S_{1}$, $S_{3}$, and $S_{4}$ even when given the correct subspace structure as input, suggesting that multimodal initialization is less effective in these settings, likely because cross-modal alignment optimization becomes increasingly challenging as subspace dimensionality grows.

### 3.2 MSIVA detects latent subspace structures in neuroimaging data

The model order was estimated for each neuroimaging dataset using three information-theoretic criteria (Y.-O. Li et al., 2007). Twelve sources captured the majority of the information in the patient dataset and a substantial amount of the information in the UK Biobank (UKB) dataset (Supplementary Material, Section 6, Fig. S5). Hence, 12 sources were included in each subspace structure.

**Fig. 5.**
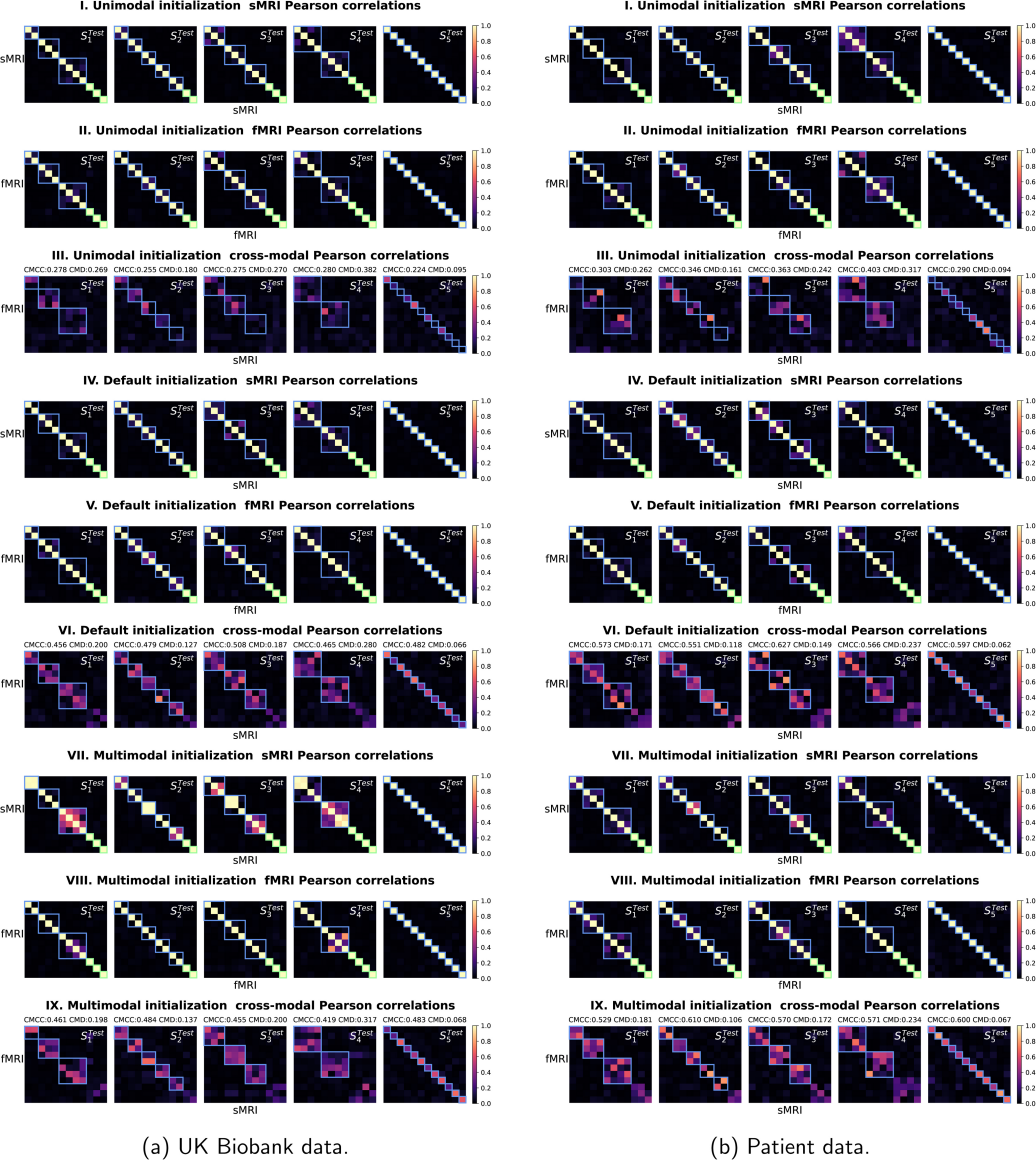
Within-modal and cross-modal Pearson correlations of the recovered neuroimaging sources before applying post-hoc CCA. (a) UK Biobank data. (b) Patient data. Cross-modal subspaces are highlighted in blue and modality-specific subspaces in green. Each workflow occupies three rows: within-modal correlations for sMRI (rows I, IV, VII), within-modal correlations for fMRI (rows II, V, VIII), and cross-modal correlations (rows III, VI, IX), corresponding to the unimodal, default, and multimodal initialization workflows, respectively. Within-modal correlation patterns indicated negligible residual dependence between subspaces (rows I, II, IV, V, VII, and VIII). The default and multimodal initialization workflows (rows VI and IX) showed stronger cross-modal correlations (higher CMCCs and lower CMDs) than the unimodal initialization workflow (row III).

**Fig. 6.**
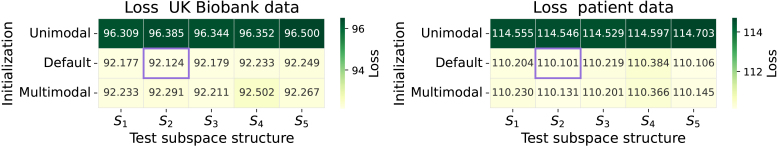
Final MISA loss values (lower is better) on neuroimaging data. The default initialization workflow with the subspace structure $S_{2}$ consistently yielded the lowest loss values in both multimodal neuroimaging datasets, making it the optimal approach for capturing the latent subspace structure in these datasets.

We applied three initialization workflows and five subspace structures to two large multimodal neuroimaging datasets separately—the UKB dataset and the combined patient dataset—to detect their latent subspace structures. In the UKB dataset, within-modal self-correlation patterns (Fig. 5a, rows I, II, IV, V, VII, and VIII) indicated negligible residual dependence between subspaces, confirming that the method successfully minimized inter-subspace dependence as intended. Note that dependence within a subspace is acceptable, but dependence between subspaces is not. Both the default and multimodal initialization workflows (Fig. 5a, rows VI and IX) recovered stronger cross-modal correlations (higher CMCCs and lower CMDs) than the unimodal initialization workflow (Fig. 5a, row III) for all predefined subspace structures. However, the multimodal initialization workflow produced notably high within-modal RDC values for sMRI within certain subspaces (e.g., the 2×2 block in $S_{1}$ and the third 2×2 block in $S_{2}$), suggesting that the corresponding source pairs are nearly identical and the underlying subspace covariance is likely ill-conditioned. Nonlinear dependence measures further confirmed that sources within cross-modal subspaces were linked across modalities, while sources in different subspaces remained independent (Supplementary Material, Section 7, Fig. S6a). Among all combinations of three initialization workflows and five candidate subspace structures, the default initialization workflow (MGPCA+ICA) with the subspace structure $S_{2}$ yielded the lowest final MISA loss value of 92.124 (Fig. 6), suggesting that $S_{2}$ best fits the latent structure of this dataset.

Similarly, in the patient dataset, both the default and multimodal initialization workflows (Fig. 5b and Supplementary Fig. S6b, rows VI and IX) showed stronger cross-modal correlations than the unimodal initialization workflow (Fig. 5b and Supplementary Fig. S6b, row III) for all five subspace structures. As with the UKB dataset, the default initialization workflow with the subspace structure $S_{2}$ yielded the lowest final loss value of 110.101 in the patient dataset (Fig. 6). Together, these results suggest that the default initialization workflow combined with the subspace structure $S_{2}$ (five linked 2D subspaces) provides a better fit to the underlying statistical relationships in both datasets.

### 3.3 MSIVA reveals linked phenotypic and neuropsychiatric biomarkers

**Fig. 7.**
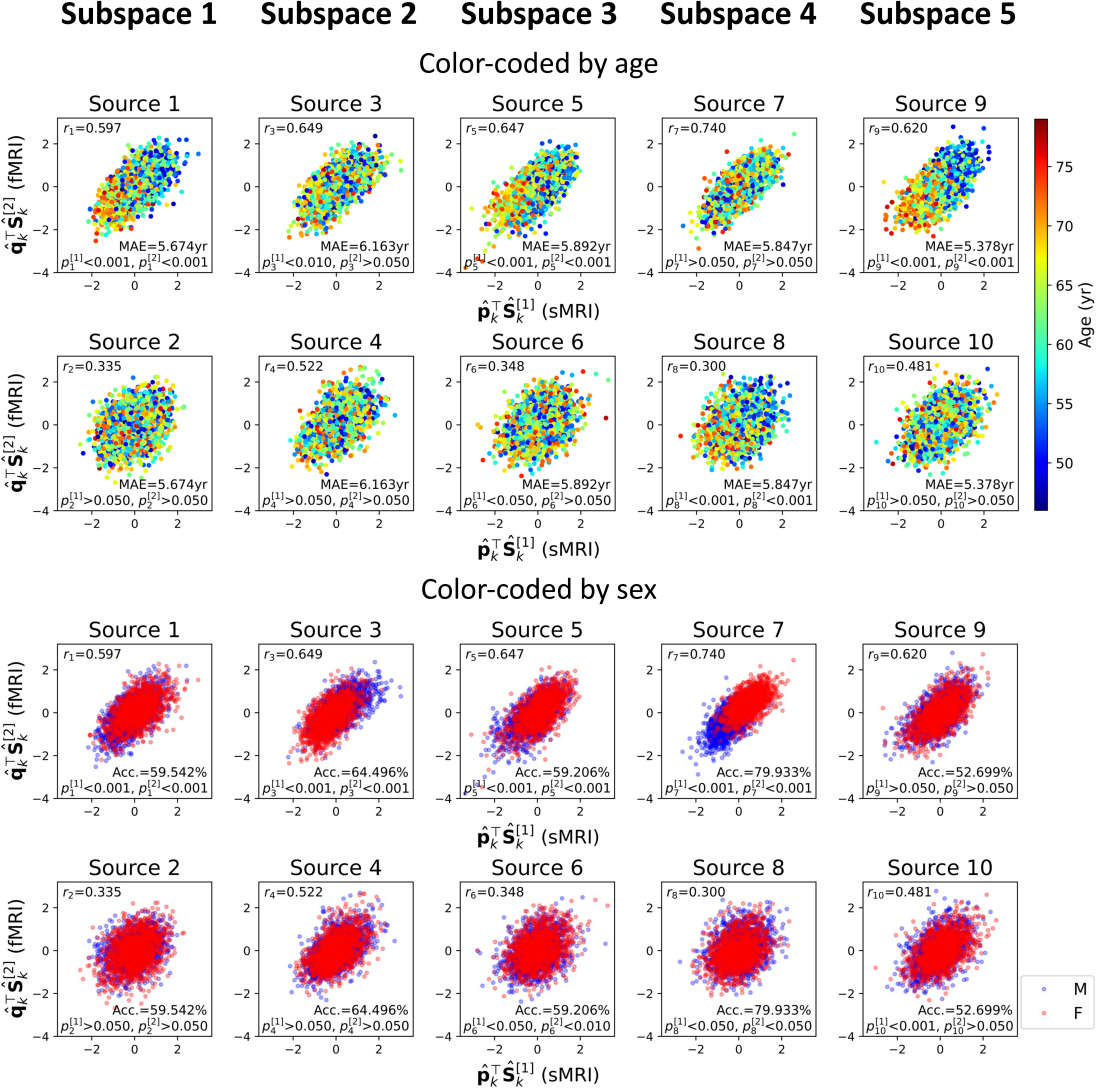
Post-CCA sources from MSIVA $S_{2}$ cross-modal subspaces, color-coded by age or sex (UK Biobank neuroimaging data). Rows I and II show age effects; rows III and IV show sex effects. The Pearson correlation coefficient ($r_{c}$, where c is the source index) indicates the cross-modal correlation of post-CCA sources (high $r_{c}$ means similar expression profiles, not similar spatial organization). We performed age regression and sex classification, measured by mean absolute error (MAE; lower is better) and balanced accuracy (Acc.; higher is better), to assess associations between MSIVA linked sources from each cross-modal subspace and phenotype measures. Sources from subspace 5 yielded the best age regression performance (MAE =5.378 years), while sources from subspace 4 achieved the best sex classification performance (Acc. =79.933% ). The p-value indicates group differences (age: younger vs. older; sex: male vs. female) for sMRI ($p^{\left[1\right]}$) and fMRI ($p^{\left[2\right]}$) sources separately (two-sample t-test; all reported p-values were Bonferroni-corrected for 20 comparisons). Across both modalities, cross-modal sources 1, 5, 8, and 9 were significantly associated with age ($p^{\left[1\right]}<0.001,p^{\left[2\right]}<0.001$ ), and sources 1, 3, 5, 6, 7, and 8 were significantly associated with sex differences ($p^{\left[1\right]}<0.05,p^{\left[2\right]}<0.05$ ).

**Fig. 8.**
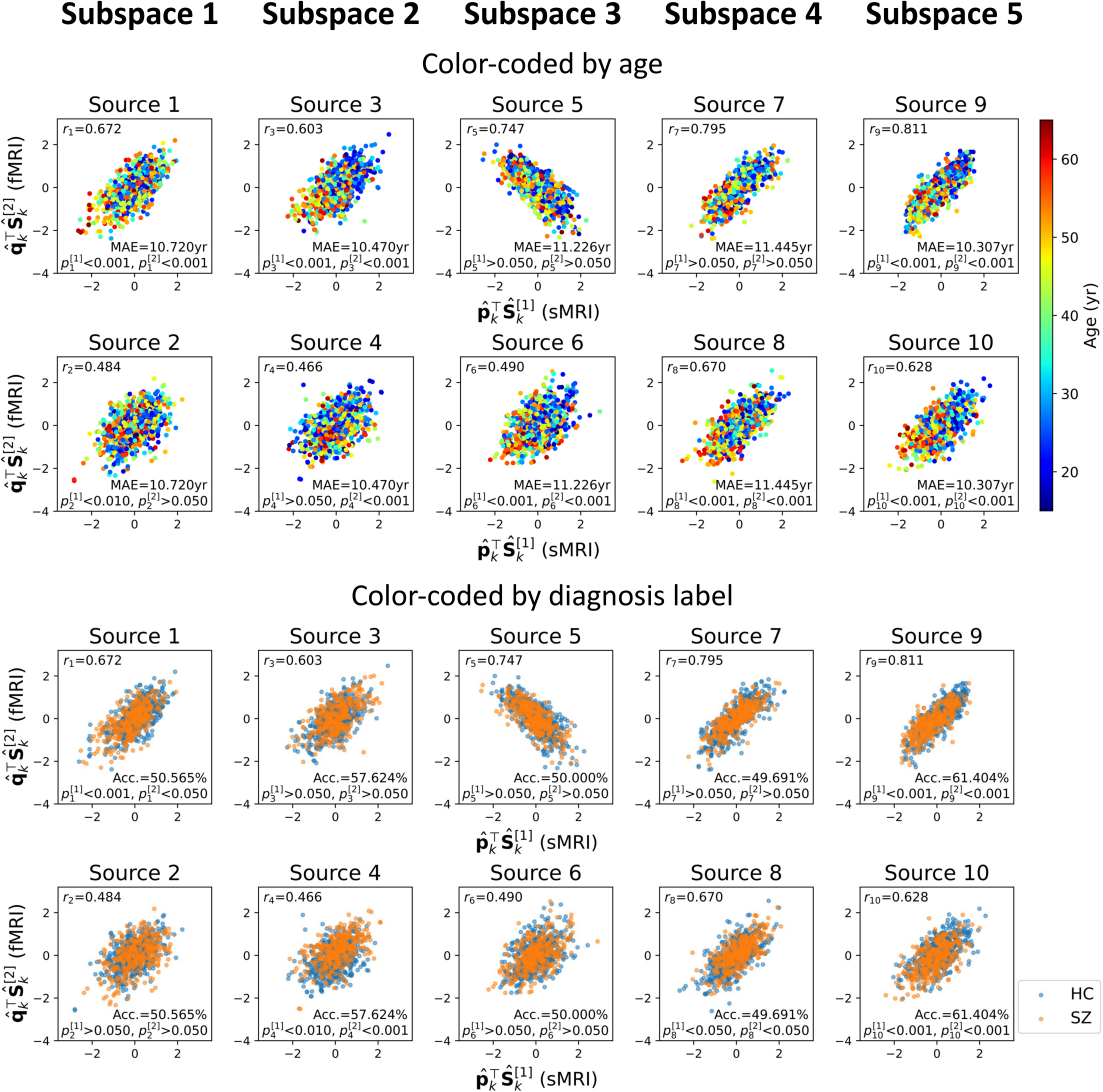
Post-CCA sources from MSIVA $S_{2}$ cross-modal subspaces, color-coded by age or diagnosis labels (patient neuroimaging data). Rows I and II show age effects; rows III and IV show SZ-related effects. The Pearson correlation coefficient ($r_{c}$, where c is the source index) shows the cross-modal correlation of post-CCA sources (high $r_{c}$ means similar expression profiles, not similar spatial organization). We performed age regression and diagnosis classification, measured by mean absolute error (MAE; lower is better) and balanced accuracy (Acc.; higher is better), to assess associations between MSIVA linked sources from each cross-modal subspace and phenotype measures. Sources from subspace 5 yielded the best age regression and diagnosis classification performance (MAE =10.307 years; Acc. =61.404% ), whereas subspace 2 performed similarly (MAE =10.470 years; Acc. =57.624% ). The p-value indicates group differences (age: younger vs. older; diagnosis: HC vs. SZ) for sMRI ($p^{\left[1\right]}$) and fMRI ($p^{\left[2\right]}$) sources separately (two-sample t-test; all reported p-values were Bonferroni-corrected for 20 comparisons). Cross-modal sources 1, 3, 6, 8, 9, and 10 were significantly associated with age ($p^{\left[1\right]}<0.001,p^{\left[2\right]}<0.001$ ). Sources 1, 4, 8, 9, and 10 exhibited significant associations with SZ-related effects ($p^{\left[1\right]}<0.05,p^{\left[2\right]}<0.05$ ).

After identifying neuroimaging sources, we examined whether the linked sources were biologically meaningful by evaluating brain-phenotype relationships between phenotype variables and the neuroimaging sources estimated by MSIVA using the default initialization and optimal subspace structure $S_{2}$ (selected based on Fig. 6). In the UKB dataset, CCA projections within each linked subspace showed that cross-modal sources 1, 5, 8, and 9 were significantly associated with age ($p^{\left[1\right]}<0.001,p^{\left[2\right]}<0.001$ ; two-sample t-test, Bonferroni correction for 20 comparisons; Fig. 7, rows I and II). The strongest association was observed for source 9 in subspace 5. Cross-modal sources 1, 3, 5, 6, 7, and 8 showed significant sex differences ($p^{\left[1\right]}<0.05,p^{\left[2\right]}<0.05$ ; Fig. 7, rows III and IV), most prominently for source 7 in subspace 4. Age and sex prediction using post-CCA sources further confirmed these associations: subspace 5 achieved the lowest age prediction MAE (5.378 years), and subspace 4 achieved the highest sex classification balanced accuracy (79.933% ). In the patient dataset, cross-modal CCA projections within each linked subspace revealed significant age effects in cross-modal sources 1, 3, 6, 8, 9, and 10 ($p^{\left[1\right]}<0.001,p^{\left[2\right]}<0.001$ ; Fig. 8, rows I and II), as well as SZ-related effects in sources 1, 4, 8, 9, and 10 ($p^{\left[1\right]}<0.05,p^{\left[2\right]}<0.05$ ; Fig. 8, rows III and IV). Age regression and diagnosis classification showed that subspace 5 achieved the lowest age regression MAE (10.307 years) and the highest diagnosis classification balanced accuracy (61.404% ). Sex effects were not significant in the patient dataset (Supplementary Material, Section 8, Fig. S7).

**Fig. 9.**
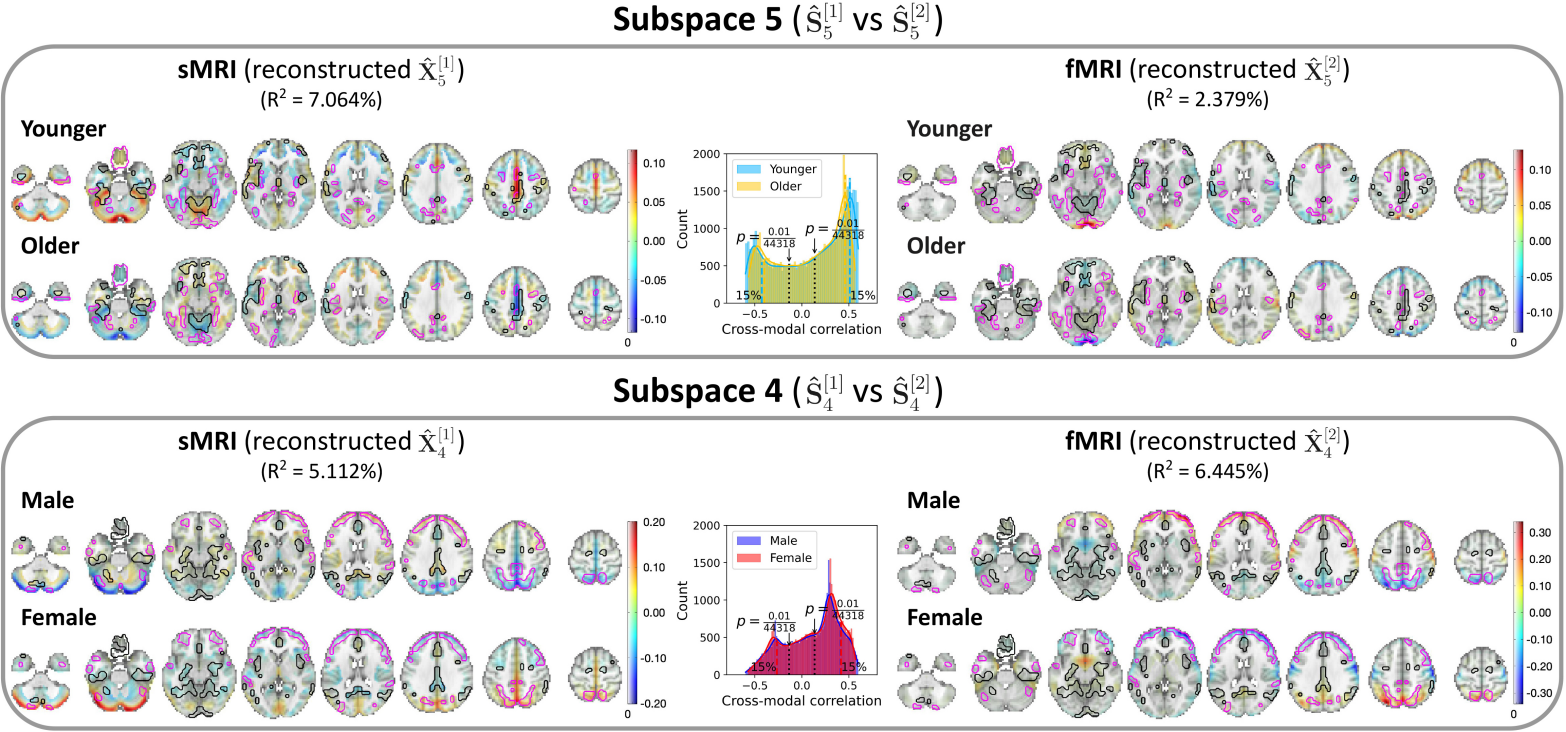
Spatial maps of group-specific reconstructed data from MSIVA $S_{2}$ sources related to age and sex effects (UK Biobank neuroimaging data). Axial slices show the geometric median of the reconstructed data (${\hat{\text{X}}}_{k}^{\left[m\right]}$) for each modality (sMRI, fMRI) and each group (younger: 46−62 years, older: 63−79 years, defined by median split; male vs. female). Voxel intensity is mapped to both color hue and opacity. Contours highlight brain regions with top 15% of voxelwise cross-modal correlations for each group (negative correlations: black; positive correlations: magenta). Histograms show voxelwise cross-modal correlations for each group (colored dashed lines: top 15% of negative and positive correlations per group; black dotted lines: $p=\frac{0.01}{44318}$ , Bonferroni correction for 44318 voxels). The reported $R^{2}$ indicates the proportion of variance captured by the subspace in each modality.

**Fig. 10.**
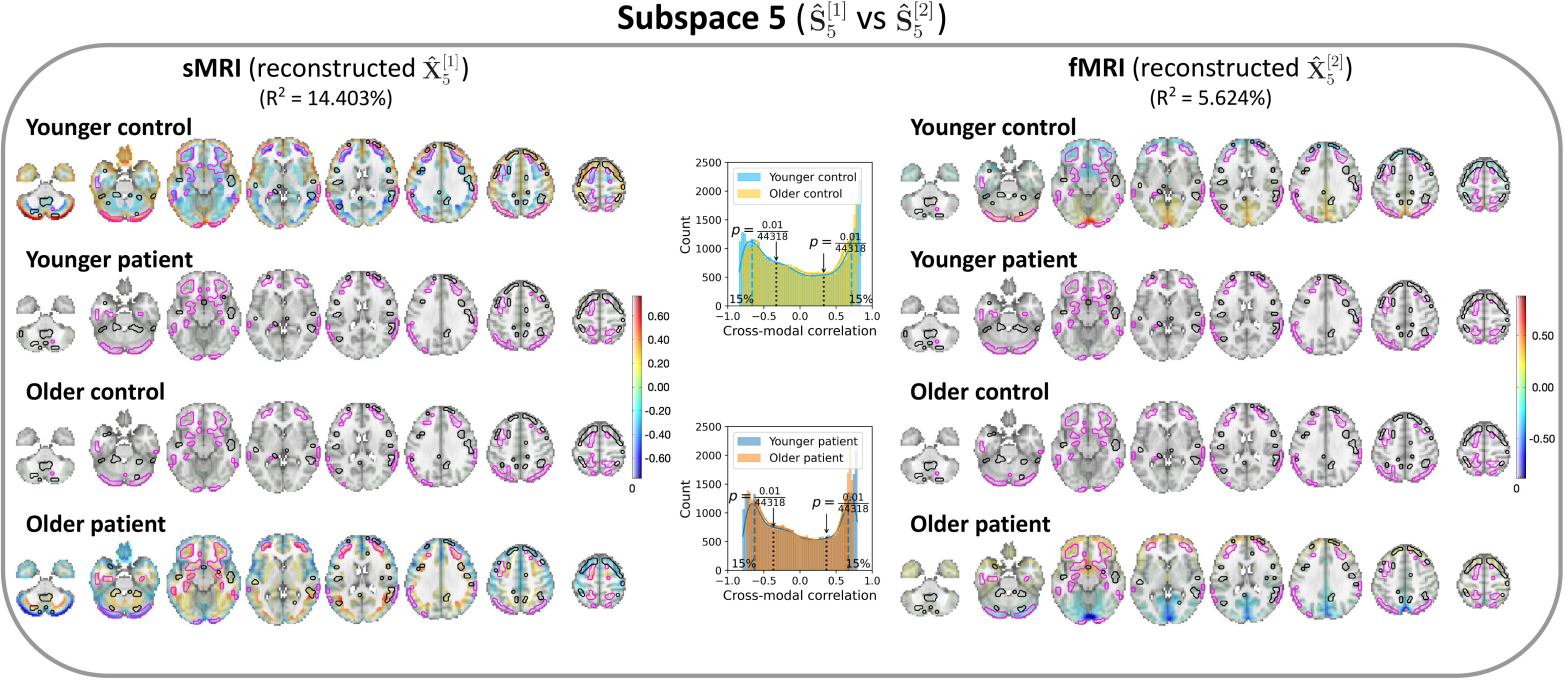
Spatial maps of group-specific reconstructed data from MSIVA $S_{2}$ sources related to age and SZ interaction effects (patient neuroimaging data). Axial slices show the geometric median of the reconstructed data (${\hat{\text{X}}}_{k}^{\left[m\right]}$) for each modality (sMRI, fMRI) and each group (younger: 15−38 years, older: 39−65 years, defined by median split; control vs. patient). Voxel intensity is mapped to both color hue and opacity. Contours highlight brain regions with top 15% of voxelwise cross-modal correlations for each group (negative correlations: black; positive correlations: magenta). Histograms show voxelwise cross-modal correlations for each group (colored dashed lines: top 15% of negative and positive correlations per group; black dotted lines: $p=\frac{0.01}{44318}$ , Bonferroni correction for 44318 voxels). The reported $R^{2}$ indicates the proportion of variance captured by the subspace in each modality.

We used dual-coded visualization (Allen et al., 2012) to display modality- and group-specific geometric median spatial maps of the reconstructed data ${\hat{\text{X}}}_{k}^{\left[m\right]}={\hat{\text{A}}}_{k}^{\left[m\right]}{\hat{\text{S}}}_{k}^{\left[m\right]}$ for each representative subspace k (Figs. 9 and 10), with voxel intensity mapped to both color hue and opacity. Contours highlight brain regions with top 15% of voxelwise cross-modal correlations for each group (negative correlations: black; positive correlations: magenta), with small clusters of voxels removed using morphological dilation and erosion.

In the UKB dataset, source 9 from subspace 5 showed the strongest age effect, while source 7 from subspace 4 showed the strongest sex effect (Fig. 9). *Subspace 5*: Age effects were observed in the cerebellum, precentral gyrus, cingulate gyrus, and paracingulate gyrus in sMRI, and in the occipital pole, lateral occipital cortex, superior frontal gyrus, and precuneus in fMRI. In particular, younger subjects (< median age in the UKB dataset; 46−62 years) showed higher positive voxel intensities in these regions, while older subjects (≥ median age in the UKB dataset; 63−79 years) showed negative intensities in the same regions. *Subspace 4*: Sex effects were observed in the frontal lobe, occipital lobe, and precuneus in both sMRI and fMRI. Female participants exhibited strong positive intensities in the cerebellum (sMRI), lateral occipital cortex (fMRI), subcallosal area (fMRI), and precuneus cortex (sMRI and fMRI), and negative intensities in the frontal pole and postcentral gyrus (fMRI). Male participants showed the opposite patterns. Spatial maps for the remaining MSIVA $S_{2}$ cross-modal subspaces in the UKB dataset are presented in Supplementary Material, Section 9, Figure S8.

In the patient dataset, sources from subspace 5 were significantly associated with age and diagnosis groups (Fig. 10). Younger control participants (< median age in the patient dataset; 15–38 years) showed high positive intensities in the cerebellum, temporal pole, and frontal operculum cortex in sMRI, as well as in the lingual gyrus, occipital pole, and precuneus cortex in fMRI. They also exhibited negative intensities in the middle temporal gyrus, inferior temporal gyrus, and occipital fusiform gyrus in sMRI. Additionally, both strong positive and negative voxel intensities were observed in the frontal lobe in sMRI. Compared to age-matched controls, the older patient group (≥ median age in the patient dataset; 39–65 years) showed decreased sMRI intensities in the cerebellum, paracingulate gyrus, insular cortex, frontal pole, middle frontal gyrus, and inferior frontal gyrus; increased sMRI intensities in the middle temporal gyrus and lateral occipital cortex; and reduced fMRI intensities in the lingual gyrus, precuneus cortex, and occipital pole. Spatial maps for the remaining MSIVA $S_{2}$ linked subspaces in the patient dataset are shown in Supplementary Material, Section 9, Figure S9.

In subspace 5, the number of voxels with significant cross-modal correlations (p<0.01 , Bonferroni correction for 44318 voxels) was 18.6% lower in older SZ patients (25623 voxels) than in age-matched controls (31482 voxels). Consistent reductions were also observed in the other three linked subspaces (Supplementary Material, Section 9, Fig. S10, subspaces 1–3), suggesting decreased coupling between brain structure and function in older patients with SZ.

### 3.4 Brain-age gap is associated with lifestyle factors and cognitive functions

**Fig. 11.**
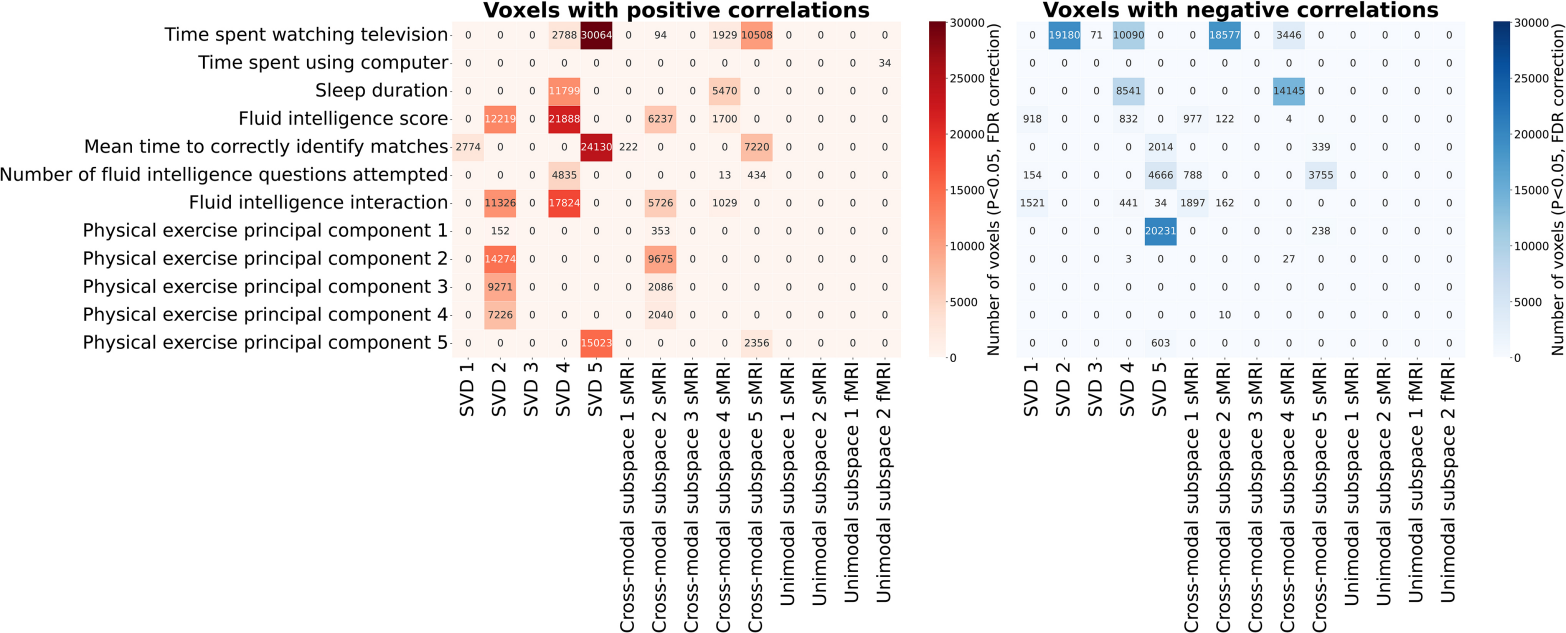
Number of voxels with significant Pearson correlations between the brain-age delta $\delta_{2p}$ and phenotype variables. The brain-age delta showed significant positive associations (left panel) and negative associations (right panel) with phenotype variables including physical exercise, time spent watching TV, sleep duration, and fluid intelligence (p<0.05 , false discovery rate correction across 44318 voxels, 25 phenotype variables, and 14 predictors).

We performed a two-stage voxelwise brain-age delta analysis using the UKB sources estimated by MSIVA with the default initialization and optimal subspace structure $S_{2}$ (Section 2.6; Supplementary Material, Section 2). We investigated how the brain-age gap was associated with other phenotype variables at the voxel level by computing the Pearson correlation between the brain-age delta $\delta_{2p}$ and each phenotype variable for each voxel. To examine effects specific to shared multimodal variability, we applied voxelwise singular value decomposition (SVD) to the combined reconstructed data from both modalities (${\hat{\text{X}}}_{k}^{\left[1\right]}$ and ${\hat{\text{X}}}_{k}^{\left[2\right]}$) for each of the five cross-modal subspaces. The brain-age delta $\delta_{2p}$ corresponding to the top SVD-shared voxel-level features from cross-modal subspaces 2, 4, and 5 showed significant associations with several phenotype variables, including time spent watching TV, sleep duration, fluid intelligence, and physical exercise (Fig. 11). In particular, predictor 5 (the SVD-shared feature from cross-modal subspace 5), which showed the strongest age association (Fig. 7 and Supplementary Material, Section 2, Fig. S1), was positively correlated with time spent watching TV and mean time to correctly identify matches (a measure of cognitive performance) and negatively correlated with the first principal component of physical exercise variables.

**Fig. 12.**
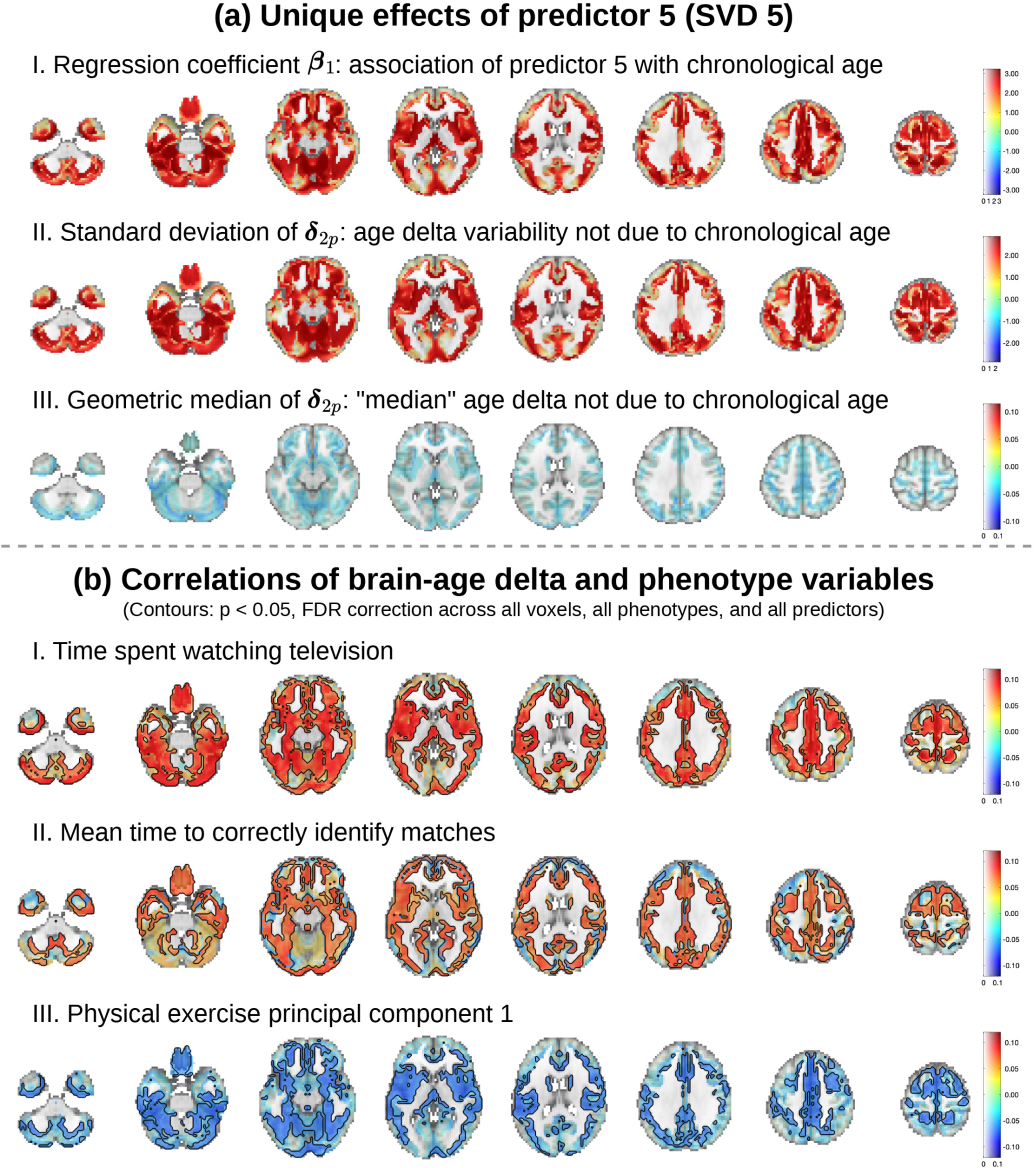
Spatial maps of predictor 5 (SVD 5) from brain-age delta analysis. (a) Spatial maps of the regression coefficient $\beta_{1}$, the standard deviation of the brain-age delta $\delta_{2p}$ , and the geometric median of $\delta_{2p}$ . Voxel values are mapped to both color hue and opacity. (b) Voxelwise correlations between $\delta_{2p}$ and three phenotype variables: time spent watching TV, mean time to correctly identify matches, and the first principal component of physical exercise variables (summarizing multiple exercise measures). Voxelwise correlations are mapped to both color hue and opacity. Contours outline brain regions with significant correlations (p<0.05 , false discovery rate correction across 44318 voxels, 25 phenotype variables, and 14 predictors). 14431 voxels show significant correlations in all three spatial maps simultaneously.

Figure 12 shows the relevant spatial maps for predictor 5 (SVD 5). Given subspace 5’s strong association with chronological age (Fig. 7), large positive values dominated the spatial maps of the regression coefficient $\beta_{1}$ and the standard deviation of $\delta_{2p}$ (Fig. 12a, rows I and II). The geometric median of $\delta_{2p}$ was slightly negative (Fig. 12a, row III), indicating that biological age was slightly lower than chronological age (the brain appeared younger). We also present spatial maps for the three phenotype variables most strongly associated with $\delta_{2p}$ : time spent watching TV, mean time to correctly identify matches, and the first principal component of physical exercise variables (Fig. 12b). Significant effects were observed in the cerebellum, postcentral gyrus, cingulate gyrus, precuneus cortex, occipital lobe, and caudate nucleus for time to watch TV; the frontal pole, precentral gyrus, and insular cortex for time to identify matches; and the cerebellum, occipital fusiform gyrus, and caudate nucleus for the first principal component of physical exercise measures. If the correlation on the spatial map is negative (as for the first principal component of physical exercise), $\delta_{2p}$ decreases as the phenotype score increases, and the brain appears younger. If it is positive (as for time to watch TV or identify matches), $\delta_{2p}$ increases as the phenotype score increases, and the brain appears older. Therefore, more physical exercise corresponded to decreased brain-age delta (younger-appearing brain), whereas more time spent watching TV or identifying correct matches corresponded to increased brain-age delta (older-appearing brain). These findings indicate that increased physical activity and reduced TV time may benefit brain health.

## 4 Discussion

We present Multimodal Subspace Independent Vector Analysis (MSIVA), a novel multivariate method for capturing both cross-modal and unimodal sources. In synthetic data experiments, MSIVA with unimodal and default initialization successfully identified the ground-truth subspace structure when provided as input, as confirmed by MISI and interference matrices. Moreover, the correct subspace structure consistently yielded the lowest loss values across all five test subspace structures. When applied to two large multimodal neuroimaging datasets, MSIVA identified the best-fitting latent subspace structure from five candidates. Among all combinations of initialization workflows and subspace structures, the default initialization method with the subspace structure $S_{2}$ yielded the lowest loss value in both neuroimaging datasets, indicating this combination best captured the underlying relationships. CCA projections within each cross-modal subspace were strongly associated with age, sex and SZ-related effects, as confirmed by phenotype prediction tasks. Moreover, voxelwise brain-age delta analysis on the UKB dataset revealed significant correlations between the voxel-level brain-age gap and non-imaging phenotype variables, including lifestyle factors and cognitive performance measures.

### 4.1 Recommendations for model order and candidate subspace structures

The success of MSIVA depends on properly identifying the model order and selecting appropriate candidate subspace structures. We propose estimating the number of sources using the common information derived from MGPCA, guided by information-theoretic criteria (Y.-O. Li et al., 2007). Based on evidence that functional imaging sources form hierarchical clusters ranging from two to four dimensions (Ke et al., 2019; Ma et al., 2010, 2011), we recommend evaluating subspace sizes within that range, while acknowledging that broader configurations warrant future investigation.

### 4.2 Evaluation of initialization workflows

We evaluated three initialization workflows that captured different levels of cross-modal information. Interestingly, the default and unimodal initialization workflows achieved comparable performance and outperformed the multimodal initialization workflow. One possible explanation is that multimodal initialization may overfit to cross-modal information. Conversely, unimodal initialization does not take advantage of any cross-modal information, risking misalignment that may be unrecoverable during optimization. The default initialization workflow, which captures an intermediate level of cross-modal information, appears to strike the best balance, consistent with the within- and cross-modal correlations of the source estimates produced by each workflow prior to optimization (Supplementary Material, Section 4).

### 4.3 Comparison of MSIVA and MMIVA

MSIVA can be viewed as an extension of MMIVA that differs in two key ways: (1) MSIVA uses a different default initialization method (MSIVA: MGPCA+ICA; MMIVA: MGPCA+GICA), and (2) MSIVA supports flexible subspace structures (MSIVA: $S_{1}-S_{5}$ and beyond; MMIVA: a fixed identity-matrix structure such as $S_{5}$). To further investigate the relationships between the sources estimated by each method, we compared MSIVA (with default initialization and subspace structure $S_{2}$) and MMIVA by using MSIVA $S_{2}$ sources to predict MMIVA sources and vice versa. We found that each pair of MSIVA $S_{2}$ cross-modal sources explained variance in more than two MMIVA sources, while each pair of matched MMIVA sources also explained variance in more than two MSIVA $S_{2}$ sources (Supplementary Material, Section 10, Figs. S11–S14). Therefore, MSIVA and MMIVA distribute variability across their sources in different ways, with no perfect one-to-one mapping between MSIVA $S_{2}$ and MMIVA sources. The mismatch appeared more pronounced in the patient dataset than in the UKB dataset, possibly due to characteristics of the patient data such as greater population heterogeneity and smaller sample sizes. Furthermore, the cross-modal sources from MSIVA $S_{2}$ more accurately predicted age and sex in the UKB dataset and diagnosis labels in the patient dataset, compared to the paired sources from MMIVA, indicating that the two-dimensional subspaces in MSIVA $S_{2}$ better capture phenotype-related variability than the pairs of one-dimensional sources in MMIVA (Supplementary Material, Section 10, Table S2).

### 4.4 Brain regions associated with phenotypes and disorders

MSIVA learns complex statistical relationships across two neuroimaging modalities, gray matter (GM) maps from T1-weighted structural MRI (sMRI) and mean-scaled amplitude of low-frequency fluctuations (mALFF) maps from resting-state functional MRI (fMRI), by identifying cross-modal dependence in high-dimensional subspaces. This approach reveals structurally and functionally relevant brain networks and identifies source groups associated with specific phenotypes and disorders.

In the UKB dataset, four cross-modal sources (1, 5, 8, and 9) were significantly associated with age, with source 9 from subspace 5 showing the strongest association (Fig. 7). Spatial maps reconstructed from subspace 5 revealed that older participants exhibited reduced sMRI intensities in the cerebellum, precentral gyrus, cingulate gyrus, and paracingulate gyrus, as well as reduced fMRI intensities in the occipital pole, precuneus cortex, superior frontal gyrus, and lateral occipital cortex (Fig. 9). Several age-related brain regions identified in this study are consistent with findings reported in the literature. In structural imaging, cerebellar volume loss has been associated with aging (Jernigan et al., 2001; Luft et al., 1999; Romero et al., 2021), and strong and weak age effects have been reported in the precentral and cingulate gyri, respectively (Hogstrom et al., 2013). In functional imaging, reductions in the visual areas of the mALFF maps are consistent with our previous findings using MMIVA (Silva et al., 2024) and prior work linking the occipital lobe to aging in functional networks (Scheinost et al., 2015).

Regarding sex effects, six sources (1, 3, 5, 6, 7, and 8) showed significant associations with sex, with source 7 from subspace 4 showing the strongest effect (Fig. 7). Spatial maps from subspace 4 showed that compared to females, males had lower sMRI intensities in the cerebellum and precuneus, lower fMRI intensities in the lateral occipital cortex and precuneus, and higher fMRI intensities in the frontal pole and postcentral gyrus (Fig. 9). These findings are consistent with prior reports of sex differences in cerebellar and precuneus gray matter volume (Fan et al., 2010; Ruigrok et al., 2014), as well as in frontal and occipital areas using functional measures (Tian et al., 2011). In particular, MMIVA has similarly revealed lower cerebellar sMRI intensities in males than in females (Silva et al., 2024).

In the patient dataset, both sources in subspace 5 showed significant associations with age and SZ (Fig. 8). Using reconstructed data from subspace 5 sources, older SZ patients showed decreased sMRI intensities in the cerebellar, frontal, and insular cortices and increased intensities in the middle temporal gyrus and lateral occipital cortex, as well as reduced fMRI intensities in the precuneus, lingual gyrus, and occipital pole, compared with age-matched controls (Fig. 10). These results are consistent with previously reported SZ-related reductions in cerebellar gray matter volume (Moberget et al., 2018; Picard et al., 2008) and alterations in anatomical networks involving frontal, medial temporal, and insular cortices (Bassett et al., 2008).

Beyond resting-state fMRI, MSIVA can be applied to task-related or naturalistic data to uncover structural–functional relationships under more diverse conditions. It can also be extended to other high-dimensional modalities such as genomics, potentially advancing our understanding of the molecular mechanisms underlying brain development and disorders.

### 4.5 Limitations and future directions

A limitation of MSIVA is its reliance on a predefined set of candidate subspace structures, which may miss other viable structures, though exhaustively evaluating all potential subspace structures is computationally infeasible. Additionally, we make two simplifying assumptions on the subspace structure: cross-modal subspaces share the same dimensionality per modality, and unimodal subspaces are all one-dimensional. These assumptions, while computationally convenient, may not reflect the true latent structure of the data. In future work, we plan to apply data-driven subspace structures such as the NeuroMark template (Du et al., 2020; Fu et al., 2024), or to learn the subspace structure from the data directly in an unsupervised manner. A related limitation is the use of 12 latent sources to approximate each data modality, chosen in part for computational efficiency during combinatorial optimization, which may be insufficient to recover all multimodal links. Addressing these limitations will require further workflow optimization and *joint* model order estimation methods capable of efficiently determining and aligning higher-dimensional subspaces (Song et al., 2016).

We used the loss value to select the optimal subspace structure in the neuroimaging experiments, where ground-truth information was unavailable. However, since loss baselines vary slightly across initialization methods, loss may not always reliably indicate goodness of fit. Loss values are significantly correlated with MISI values only when the same initialization workflow is used, so comparisons should be made within the same initialization workflow. We also recommend evaluating performance using multiple complementary metrics alongside the loss value, such as the cross-modal mean correlation coefficient (CMCC) and the cross-modal minimum distance (CMD), which measure cross-modal subspace alignment without requiring ground-truth information. These metrics should be treated as diagnostics that roughly verify consistency with the evaluated subspace structure, rather than as definitive indicators of correct estimation.

While this study focused on two modalities, MSIVA is designed to handle two or more modalities, and future work could evaluate its performance with additional data modalities. Another promising direction is to explore alternative initialization methods. For instance, the geometric mean or Log-Euclidean framework—commonly regarded as more principled in applications such as diffusion tensor imaging (DTI) (Arsigny et al., 2006) and electroencephalography (EEG) (Tibermacine et al., 2024)—could serve as a valid alternative to adjust covariance matrices before aggregation in MGPCA. In addition, a key limitation of MSIVA is its linear mixing assumption, whereby each data modality is modeled as a linear mixture of latent sources. Since the true mixing process in neuroimaging data may be nonlinear due to physiological effects and nonlinear transformations involved in fMRI modeling and preprocessing, we plan to develop *nonlinear* latent variable models capable of estimating nonlinearly mixed multimodal sources.

## 5 Conclusions

Our proposed method MSIVA effectively captured both unimodal and cross-modal sources, as well as their underlying subspace structure, from multiple synthetic and neuroimaging datasets. Brain-phenotype modeling showed that MSIVA cross-modal sources were strongly associated with phenotype variables including age, sex, and psychosis. Voxelwise brain-age delta analysis further revealed that the brain-age gap was significantly correlated with lifestyle and cognitive function measures. Together, these results demonstrate that MSIVA can uncover linked phenotypic and neuropsychiatric biomarkers of brain structure and function at the voxel level from multimodal neuroimaging data.

## Supplementary Material

Supplementary Material

## Data Availability

The UK Biobank dataset can be accessed at https://www.ukbiobank.ac.uk/. The BSNIP and MPRC datasets are available through the NIMH Data Archive (NDA) https://nda.nih.gov/. The COBRE dataset is available from the Collaborative Informatics and Neuroimaging Suite (COINS) https://coins.trendscenter.org/. The FBIRN phase III dataset cannot be shared directly due to the Institutional Review Board (IRB) restrictions. Individuals interested in requesting access can contact Vince D. Calhoun (vcalhoun@gsu.edu). All code used in this study is publicly available at https://github.com/trendscenter/MSIVA. Code for brain-age delta analysis was adapted from https://www.fmrib.ox.ac.uk/datasets/BrainAgeDelta/. Code for dual-coded images was adapted from https://trendscenter.org/x/datavis/.

## Footnotes

1 A preliminary version of this work was published at the 2023 IEEE 20th International Symposium on Biomedical Imaging (ISBI) (X. Li, Adali, et al., 2023). The present study extends the conference publication and provides more comprehensive evaluation and analysis of the proposed method by (1) including the schizophrenia patient neuroimaging data analysis, (2) incorporating an identity matrix as an additional candidate subspace structure, (3) evaluating a multimodal initialization workflow, (4) conducting the voxelwise brain-age delta analysis, (5) adding extensive visualizations and interpretations of the multimodal patterns and their associations, and (6) performing a quantitative comparison of loss values and other proposed metrics.

2 Stochastic gradient descent is a valid alternative approach (see Silva et al., 2021 for the derivation of the gradients).

3 Semicolons (;) denote that matrices are stacked vertically, and commas (,) denote that matrices are stacked horizontally.

4 The sign of each source is not uniquely identifiable, hence the use of absolute values.

5 While a subspace is uniquely identifiable, the individual sources within it are subject to sign, scale, and permutation indeterminacies.

6 The modality-specific mixing matrix was estimated as the least-squares solution: ${\hat{\text{A}}}^{\left[m\right]}={\text{X}}^{\left[m\right]}{\hat{\text{S}}}^{{\left[m\right]}^{⊤}}{\left({\hat{\text{S}}}^{\left[m\right]}{\hat{\text{S}}}^{{\left[m\right]}^{⊤}}\right)}^{-1}$ .

7 For each voxel v and each cross-modal subspace k, we utilized singular value decomposition (SVD) across all modalities (${\hat{\text{X}}}_{k\left[v,:\right]}^{\left[1\right]},{\hat{\text{X}}}_{k\left[v,:\right]}^{\left[2\right]}$) to capture *shared* reconstructed multimodal information of each cross-modal subspace.
