# Supplementary material for: Multimodal subspace independent vector analysis effectively captures latent relationships between brain structure and function
Source: Imaging Neurosci (Camb). 2026 Jun 18;4:IMAG.a.1266. doi: 10.1162/IMAG.a.1266 (PMC13281777; doi:10.1162/IMAG.a.1266)
Supplement: Supplementary Material [file IMAG.a.1266_supp.pdf]

Supplementary Material

1 Data acquisition and preprocessing

1.1 UK Biobank dataset

1.1.1 Acquisition parameters

T1-weighted structural MRI (sMRI) scans were acquired using a 3D MPRAGE sequence with the following parameters: repetition time (TR) $= 2000$ms, inversion time (TI) $= 880$ms, in-plane acceleration factor $= 2$, voxel size $= 1 \times 1 \times 1\text{mm}^3$, acquisition matrix $= 208 \times 256 \times 256$. Resting-state functional MRI (fMRI) scans were acquired with the following parameters: TR $= 735$ms, echo time (TE) $= 39$ms, multiband factor $= 8$, in-plane acceleration factor $= 1$, flip angle $= 52°$, voxel size $= 2.4 \times 2.4 \times 2.4\text{mm}^3$, acquisition matrix $= 88 \times 88 \times 64$ (Alfaro-Almagro et al., 2018).

1.1.2 Preprocessing steps

sMRI preprocessing. We performed tissue segmentation and normalization to the Montreal Neurological Institute (MNI) template using SPM12 (http://www.fil.ion.ucl.ac.uk/spm/) (Ashburner et al., 2021), yielding gray matter (GM), white matter (WM), and cerebrospinal fluid (CSF) tissue probability maps. The normalized GM tissue probability maps were then spatially smoothed using a 10mm full width at half maximum (FWHM) Gaussian kernel and resampled to $3 \times 3 \times 3\text{mm}^3$ resolution. A group-level GM mask was constructed as follows. The normalized GM tissue probability maps at $1 \times 1 \times 1\text{mm}^3$ resolution were averaged across all subjects to produce a group-average GM map. This map was binarized using a threshold of 0.2 and resampled to $3 \times 3 \times 3\text{mm}^3$ resolution, resulting in a binary mask of 44318 voxels.

fMRI preprocessing. We utilized distortion-corrected, FIX-denoised (Griffanti et al., 2014) and normalized fMRI data from the UK Biobank data resource. For each subject, the resulting voxel-wise time series was bandpass filtered (0.01–0.08Hz) to remove low-frequency drift and physiological noise, then transformed to the frequency domain via fast Fourier transform (FFT). The amplitude of low-frequency fluctuations (ALFF) was computed as the mean square root of the power spectrum across 0.01–0.08Hz (Zang et al., 2007). To improve test-retest reliability (Zhao et al., 2018), a subject-specific mean-scaled ALFF (mALFF) map was obtained by dividing each subject's ALFF map by its global mean ALFF value. The mALFF maps were then smoothed using a 6mm FWHM Gaussian filter and resampled to

$3 \times 3 \times 3\text{mm}^3$ resolution. Finally, the same group-average GM mask was applied to the mALFF maps, resulting in 44318 voxels.

1.2 Patient datasets

1.2.1 Acquisition parameters

BSNIP. We used the BSNIP dataset collected at two sites: (1) Baltimore (3-Tesla Siemens Trio Tim) and (2) Hartford (3-Tesla Siemens Allegra). T1-weighted MPRAGE scans were acquired using the following parameters: TR $= 6.7$ms, TE $= 3.1$ms, flip angle $= 8°$, matrix size $= 256 \times 240$, number of sagittal slices $= 170$, slice thickness $= 1$mm, voxel size $= 1 \times 1 \times 1.2\text{mm}^3$, total scan time $= 10 : 52.6$min (Giakoumatos et al., 2015). Resting-state fMRI scans were acquired with the following parameters: (1) Baltimore, TR $= 2210$ms, TE $= 30$ms, flip angle $= 70°$, number of slices $= 36$, voxel size $= 3.4 \times 3.4 \times 4\text{mm}^3$, and 140 time points; (2) Hartford, TR $= 1500$ms, TE $= 27$ms, flip angle $= 70°$, number of slices $= 29$, voxel size $= 3.4 \times 3.4 \times 5\text{mm}^3$, and 210 time points.

COBRE. The COBRE dataset was collected at a single site using a 3-Tesla Siemens Tim Trio scanner. A high-resolution T1-weighted multi-echo MPRAGE sequence was used with the following parameters: TR $= 2530$ms, TI $= 900$ms, TE $= [1.64, 3.50, 5.36, 7.22, 9.08]$ms, number of echoes $= 5$, flip angle $= 7°$, pixel bandwidth $= 650$Hz, field of view (FOV) $= 256 \times 256\text{mm}^2$, slab thickness $= 176$mm, acquisition matrix $= 256 \times 256 \times 176$, voxel size $= 1 \times 1 \times 1\text{mm}^3$, total scan time $= 6$min. Resting-state fMRI scans were collected with a single-shot full k-space echo-planar imaging (EPI) sequence: TR $= 2000$ms, TE $= 29$ms, flip angle $= 75°$, FOV $= 240 \times 240\text{mm}^2$, matrix size $= 64 \times 64$, number of slices $= 32$, slice gap $= 1.05$mm, voxel size $= 3.75 \times 3.75 \times 4.55\text{mm}^3$, and 149 volumes. Further details are available at https://fcon_1000.projects.nitrc.org/indi/retro/cobre.html.

FBIRN. The FBIRN phase III dataset was collected from seven sites: six using 3-Tesla Siemens Tim Trio scanners and one using a 3-Tesla General Electric (GE) Discovery MR750 scanner. At Siemens sites, a high-resolution MPRAGE sequence was used with the following parameters: TR $= 2300$ms, TE $= 2.94$ms, TI $= 1100$ms, flip angle $= 9°$, acquisition matrix $= 256 \times 256 \times 160$. At the GE site, an IR-SPGR sequence was used with the following parameters: TR $= 5.95$ms, TE $= 1.99$ms, TI $= 45$ms, flip angle $= 12°$, acquisition matrix $= 256 \times 256 \times 166$. All scans were collected in the sagittal plane using the following parameters: FOV $= 220 \times 220\text{mm}^2$, voxel size $= 0.86 \times 0.86 \times 1.2$ mm^3, GRAPPA/ASSET acceleration factor $= 2$, and NEX $= 1$ (Qi et al., 2022). Resting-state fMRI scans were acquired with a gradient EPI sequence using identical parameters across all seven sites: TR $= 2000$ms, TE $= 30$ms, flip angle $= 77°$, FOV $= 220 \times 220\text{mm}^2$, voxel size $= 3.4375 \times 3.4375 \times 4\text{mm}^3$, slice gap $= 1$mm, and 162

volumes (Qi et al., 2022).

MPRC. The MPRC dataset was collected at three sites, each using a different 3-Tesla Siemens scanner. T1-weighted MPRAGE scans were collected in the sagittal plane with voxel size $= 1 \times 1 \times 1$mm^3 using a Siemens Allegra scanner (TR $= 2500$ms, TE $= 4.3$ms, TI $= 1000$ms, flip angle $= 8°$) or a Siemens Trio scanner (TR $= 2300$ms, TE $= 2.9$ms, TI $= 900$ms, flip angle $= 9°$) (Schijven et al., 2023). Resting-state fMRI scans were collected using the following scanners and parameters: Siemens Allegra (TR $= 2000$ms, TE $= 27$ms, FOV $= 220 \times 220$mm^2, voxel size $= 3.44 \times 3.44 \times 4$mm^3, 150 volumes); Siemens Trio (TR $= 2210$ms, TE $= 30$ms, FOV $= 220 \times 220$mm^2, voxel size $= 3.44 \times 3.44 \times 4$mm^3, 140 volumes); and Siemens Tim Trio (TR $= 2000$ms, TE $= 30$ms, FOV $= 220 \times 220$mm^2, voxel size $= 1.72 \times 1.72 \times 4$mm^3, 444 volumes) (Qi et al., 2022).

1.2.2 Preprocessing steps

sMRI preprocessing. All sMRI datasets were preprocessed using SPM12, following the steps described in Qi et al., 2022. Specifically, the data were normalized to the MNI space, resampled to $3 \times 3 \times 3$mm^3, and segmented into GM, WM, and CSF volume maps. These GM volume maps were then smoothed using a 6mm FWHM Gaussian kernel. To identify subjects with poor segmentation quality, outlier detection was performed by computing the spatial Pearson correlation between each subject's GM map and the template.

fMRI preprocessing. All fMRI datasets were preprocessed using SPM12, following Qi et al., 2022. The pipeline consisted of: removal of the first five volumes to eliminate T1 equilibration effects; slice timing correction; realignment; normalization to the EPI template with $3 \times 3 \times 3$mm^3 resolution; spatial smoothing with a 6mm FWHM Gaussian kernel; nuisance regression of six head motion parameters, WM signal, CSF signal, and global signal from each voxelwise time course using a general linear model; and computation of mALFF maps.

2 Voxelwise brain-age delta analysis on UK Biobank data

We performed a voxelwise brain-age delta analysis using the estimated sources $\hat{\mathbf{S}}$ from MSIVA default initialization and subspace structure S_2 in the UK Biobank dataset. We describe the steps to construct imaging-derived predictors as follows.

1. **Reconstruction.** We reconstructed the modality- and subspace-specific imaging feature $\hat{\mathbf{X}}_k^{[m]} = \hat{\mathbf{A}}_k^{[m]}\hat{\mathbf{S}}_k^{[m]} \in \mathbb{R}^{V\times N}$ for each of the five cross-modal subspaces ($\hat{\mathbf{A}}_k^{[m]} \in \mathbb{R}^{V\times 2}$, $\hat{\mathbf{S}}_k^{[m]} \in \mathbb{R}^{2\times N}, k = 1,\ldots,5$) and each of the four unimodal subspaces ($\hat{\mathbf{A}}_k^{[m]} \in \mathbb{R}^{V\times 1}$, $\hat{\mathbf{S}}_k^{[m]} \in \mathbb{R}^{1\times N}, k = 6,7,8,9$), where k is the subspace index. Here, subspaces 6 and 7 contain sMRI sources only and subspaces 8 and 9 contain fMRI sources only.

2. **Singular value decomposition (SVD).** For the five cross-modal subspaces, we applied SVD to the concatenated two-modality feature matrix at each voxel, capturing the dominant shared variation between sMRI and fMRI. Specifically, for voxel v and cross-modal subspace k in the reconstructed imaging data $\hat{\mathbf{X}}_{k[v,:]}^{[m]} \in \mathbb{R}^N$, we concatenated the two modalities $\hat{\mathbf{X}}_{kv} = \left[\hat{\mathbf{X}}_{k[v,:]}^{[1]}, \hat{\mathbf{X}}_{k[v,:]}^{[2]}\right] \in \mathbb{R}^{N\times 2}$. We then normalized $\hat{\mathbf{X}}_{kv}$ across subjects (zero mean, unit variance per column) and performed SVD: $\hat{\mathbf{X}}_{kv} = \mathbf{U}\mathbf{\Sigma}\mathbf{V}^\top$. To obtain a single value per subject that captures the dominant shared variation, we multiplied $\hat{\mathbf{X}}_{kv}$ by the first right singular vector $\mathbf{V}_{[:,1]} \in \mathbb{R}^{2\times 1}$ corresponding to the largest singular value $\sigma_{\max}$: $\hat{\mathbf{X}}_{kv}^{\mathsf{SVD}} = \hat{\mathbf{X}}_{kv}\mathbf{V}_{[:,1]} \in \mathbb{R}^{N\times 1}$. We then normalized $\hat{\mathbf{X}}_{kv}^{\mathsf{SVD}}$ across subjects to obtain the normalized SVD projection $\hat{\mathbf{X}}_{kv}^{\mathsf{SVD}'}$.

3. **Partialling and normalization.** To ensure each predictor captures unique variance, we performed partialling and normalization for each modality separately (Smith et al., 2020). For sMRI, we concatenated the five cross-modal SVD projections $\hat{\mathbf{X}}_{kv}^{\mathsf{SVD}'}$ ($k = 1,\ldots,5$) and sMRI features $\hat{\mathbf{X}}_{1[v,:]}^{[1]},\ldots,\hat{\mathbf{X}}_{7[v,:]}^{[1]}$, then partialled each feature against all others and normalized the residuals across subjects, yielding the partialled sMRI predictors $\hat{\mathbf{X}}_{k[v,:]}^{[1]'}$. For fMRI, we repeated this procedure with the same SVD projections and fMRI features $\hat{\mathbf{X}}_{1[v,:]}^{[2]},\ldots,\hat{\mathbf{X}}_{5[v,:]}^{[2]},\hat{\mathbf{X}}_{8[v,:]}^{[2]},\hat{\mathbf{X}}_{9[v,:]}^{[2]}$, yielding the partialled fMRI predictors $\hat{\mathbf{X}}_{k[v,:]}^{[2]'}$.

4. **Concatenation.** For each voxel v, we assembled the final predictor matrix by concatenating: (1) the five cross-modal SVD projections from step 2 (without partialling or extra normalization) $\hat{\mathbf{X}}_{kv}^{\mathsf{SVD}'}$ ($k = 1,\ldots,5$); (2) the partialled and normalized sMRI features $\hat{\mathbf{X}}_{k[v,:]}^{[1]'}$ from the five cross-modal subspaces ($k = 1,\ldots,5$) and two sMRI-specific unimodal subspaces ($k = 6,7$), retaining the sMRI-specific residual variance not captured by the SVD projections; and (3) the partialled and normalized fMRI features $\hat{\mathbf{X}}_{k[v,:]}^{[2]'}$ from the two fMRI-specific unimodal subspaces ($k = 8,9$) only, as

the cross-modal fMRI features are already represented by the SVD projections. The final predictor matrix includes the following 14 predictors in total:

$$\hat{\mathbf{X}}_v = \left[\hat{\mathbf{X}}_{1v}^{\mathsf{SVD}'}, \ldots, \hat{\mathbf{X}}_{5v}^{\mathsf{SVD}'}, \hat{\mathbf{X}}_{1[v,:]}^{[1]'}, \ldots, \hat{\mathbf{X}}_{7[v,:]}^{[1]'}, \hat{\mathbf{X}}_{8[v,:]}^{[2]'}, \hat{\mathbf{X}}_{9[v,:]}^{[2]'}\right], \quad \hat{\mathbf{X}}_v \in \mathbb{R}^{N\times 14}. \tag{S1}$$

For each voxel v, we performed a two-stage brain-age delta analysis following Smith et al., 2019, 2020. In the first stage, we estimated the initial brain-age delta:

$$\boldsymbol{\delta}_1 = \hat{\mathbf{X}}_v \boldsymbol{\beta}_1 - \mathbf{y}, \tag{S2}$$

where $\mathbf{y} \in \mathbb{R}^N$ is demeaned chronological age, and the regression coefficient is estimated via ordinary least squares: $\boldsymbol{\beta}_1 = (\hat{\mathbf{X}}_v^\top \hat{\mathbf{X}}_v)^{-1} \hat{\mathbf{X}}_v^\top \mathbf{y} \in \mathbb{R}^{14}$.

In the second stage, we decomposed $\boldsymbol{\delta}_1$ into predictor-specific contributions while removing age dependence and other confounding factors. For each predictor i, we computed:

$$\boldsymbol{\delta}_{2i} = \hat{\mathbf{X}}_{v[:,i]} \beta_{1i} - \mathbf{Y} \boldsymbol{\beta}_{2i}, \tag{S3}$$

where $\hat{\mathbf{X}}_{v[:,i]} \beta_{1i}$ is the contribution of predictor i to $\boldsymbol{\delta}_1$, $\mathbf{Y} \in \mathbb{R}^{N\times 10}$ is a matrix of confounding variables, and $\boldsymbol{\beta}_{2i} = (\mathbf{Y}^\top \mathbf{Y})^{-1} \mathbf{Y}^\top \hat{\mathbf{X}}_{v[:,i]} \beta_{1i} \in \mathbb{R}^{10}$. The ten confounding variables are: the demeaned linear age term, the demeaned quadratic age term after regressing out the linear age effects and normalizing to match the standard deviation of the linear age term, the demeaned cubic age term after regressing out the linear and quadratic age effects and normalizing to match the standard deviation of the linear age term; sex; the interaction between sex and each of the three age terms; framewise displacement; two spatial normalization variables (one from sMRI and one from fMRI).

This process yields 14 predictor-specific brain-age delta vectors $\boldsymbol{\delta}_{2i} \in \mathbb{R}^N, i = 1, \ldots, 14$. We partialled each $\boldsymbol{\delta}_{2i}$ against all other $\boldsymbol{\delta}_{2j}$ $(j \neq i)$, yielding the partialled brain-age delta vectors $\boldsymbol{\delta}_{2pi} \in \mathbb{R}^N, i = 1, \ldots, 14$. Finally, we concatenated all partialled brain-age delta vectors into the partialled brain-age delta matrix $\boldsymbol{\delta}_{2p} = [\boldsymbol{\delta}_{2p1}, \ldots, \boldsymbol{\delta}_{2p14}] \in \mathbb{R}^{N\times 14}$.

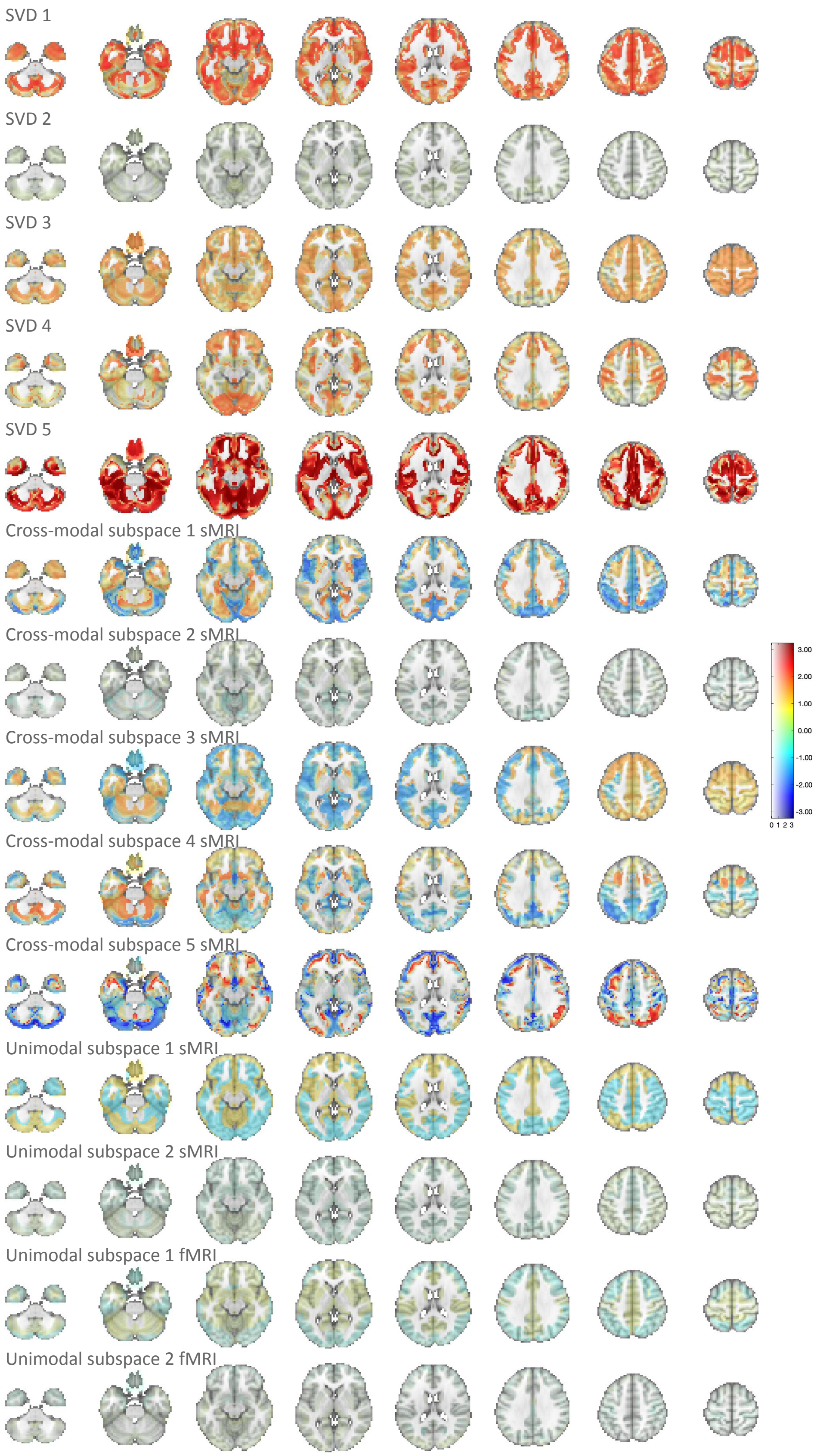

Figure S1: **Spatial maps of β_1 in voxelwise brain-age delta analysis.** Voxel intensity is mapped to both color hue and opacity. SVD 5 shows the strongest association with age among all predictors.

3 UK Biobank phenotype variables

We used 25 phenotype variables, including lifestyle measures and cognitive test scores, to investigate their associations with the brain-age delta. We describe the process of selecting the phenotype variables as follows.

We first excluded variables with extreme values from the original 64 non-imaging variables using a two-step approach:

1. For each variable ϕ, we computed the squared deviation from the median for each subject n as $d_{\phi_n} = (\phi_n - \mathrm{median}(\boldsymbol{\phi}))^2$, where $\boldsymbol{\phi} = [\phi_1, \ldots, \phi_N]$ and N is the number of subjects, yielding the vector of deviations $\boldsymbol{d}_\phi = [d_{\phi_1}, \ldots, d_{\phi_N}]$.
2. We excluded any variable ϕ for which $\max(\boldsymbol{d}_\phi) > 100 \times \mathrm{mean}(\boldsymbol{d}_\phi)$, as such variables contain extreme outliers that could skew subsequent analyses.

This initial screening retained 54 variables, including age, sex, fluid intelligence, physical activity measures, alcohol intake frequency, cognitive test scores, time spent watching TV, and sleep duration. We then applied the following steps to arrive at the final variable set:

1. We applied PCA to decompose 28 physical exercise variables into 8 principal components.
2. We removed five age-related variables highly correlated with other age variables: "age when attended assessment center", "age when first sexual intercourse", "age started wearing glasses", "years since first sexual intercourse", and "years since started wearing glasses".
3. We excluded two variables from a cognitive test ("time to answer" and "log time to answer") because their distributions were inconsistent across two different cognitive test versions used during data collection.
4. We removed the sex variable and a log-transformed score variable ("log pm score").

This selection process ultimately yielded 25 variables for subsequent analyses (Table S1).

Table S1: **54 UK Biobank phenotype variables.** The 28 physical exercise variables in purple were reduced to 8 principal components by PCA. Variables in red were excluded from brain-age delta analysis. Variables without IDs were derived by R.F.S. based on the original variables and are not part of the official UK Biobank dataset.

ID	Name
f399 2 2	number of incorrect matches in round
f400 2 2	time to complete round
f699 2 0	length of time at current address
f864 2 0	number of daysweek walked 10 minutes
f874 2 0	duration of walks
f884 2 0	number of daysweek of moderate physical activity 10 minutes
f894 2 0	duration of moderate activity
f904 2 0	number of daysweek of vigorous physical activity 10 minutes
f914 2 0	duration of vigorous activity
f943 2 0	frequency of stair climbing in last 4 weeks
f971 2 0	frequency of walking for pleasure in last 4 weeks
f981 2 0	duration walking for pleasure
f991 2 0	frequency of strenuous sports in last 4 weeks
f1001 2 0	duration of strenuous sports
f1011 2 0	frequency of light diy in last 4 weeks
f1021 2 0	duration of light diy
f1050 2 0	time spend outdoors in summer
f1060 2 0	time spent outdoors in winter
f1070 2 0	time spent watching television tv
f1080 2 0	time spent using computer
f1160 2 0	sleep duration
f1438 2 0	bread intake
f1488 2 0	tea intake
f1498 2 0	coffee intake
f1558 2 0	alcohol intake frequency
f2139 2 0	age first had sexual intercourse
f2217 2 0	age started wearing glasses or contact lenses
f2624 2 0	frequency of heavy diy in last 4 weeks
f2634 2 0	duration of heavy diy
f3637 2 0	frequency of other exercises in last 4 weeks
f3647 2 0	duration of other exercises
f4288 2 0	time to answer
f4609 2 0	longest period of depression
f20016 2 0	fluid intelligence score
f20023 2 0	mean time to correctly identify matches
f20128 2 0	number of fluid intelligence questions attempted within time limit
f21003 2 0	age when attended assessment centre
f31 0 0	sex
	total hours walked 10 minutes
	total hours moderate physical activity 10 minutes
	total hours vigorous physical activity 10 minutes
	total hours of walking for pleasure in last 4 weeks
	total hours of strenuous sports in last 4 weeks
	total hours of other exercises in last 4 weeks
	total hours of light diy in last 4 weeks
	total hours of heavy diy in last 4 weeks
	number of physical activities wrt walking for pleasure
	years since first sexual intercourse
	years since started wearing glasses
	log time to answer
	inverse log duration screen displayed
	inverse log number of attempts
	log pm score
	fluid intelligence interaction

4 Comparison of initialization workflows

The choice of weight matrix initialization can affect whether the model converges to the optimal solution. To assess the impact of different initialization workflows, we evaluated the initial source estimates produced by each initialization workflow prior to optimization. Figure S2 shows the within-modal source correlations (diagonal blocks) and cross-modal source correlations (off-diagonal blocks) for each initialization approach.

Unimodal initialization applies PCA and ICA to each data modality separately. Because each modality was processed independently without access to information from other modalities, this approach successfully separated sources within each modality, but failed to align sources across modalities or group sources into subspaces (Figure S2, row I).

In contrast, MSIVA default initialization applies MGPCA to both modalities simultaneously, followed by ICA on each dimensionality-reduced dataset separately. MGPCA identifies orthogonal components using a weighted average of the modality-specific covariance matrices, which preserves cross-modal information during dimensionality reduction. ICA then separates these MGPCA-derived components into independent sources. This hybrid approach effectively separated unimodal sources and identified substantial cross-modal linkage for subspace structures S_1, S_2, and S_5, as evidenced by the diagonal structure in the cross-modal correlations (Figure S2, row II). However, it failed to capture the cross-modal linkage for more complex subspace structures such as S_3 and S_4. Overall, the default initialization workflow achieved the lowest MISI values in four of the five evaluated subspace structures (S_1, S_2, S_3, and S_5).

Lastly, multimodal initialization sequentially applies MGPCA and GICA to both datasets. The approach performed adequately for S_5, which contains one-dimensional sources, but failed for other subspace structures containing higher-dimensional subspaces (Figure S2, row III). Specifically, it neither properly separated sources within each modality (high off-diagonal values in within-modal correlations) nor aligned sources across modalities (absence of diagonal structures in cross-modal correlations).

While perfect separation and alignment are not expected immediately after initialization—since subsequent combinatorial and numerical optimization steps will further refine these estimates—a good balance between unimodal separation and cross-modal alignment during initialization generally increases the likelihood of convergence to the optimal solution.

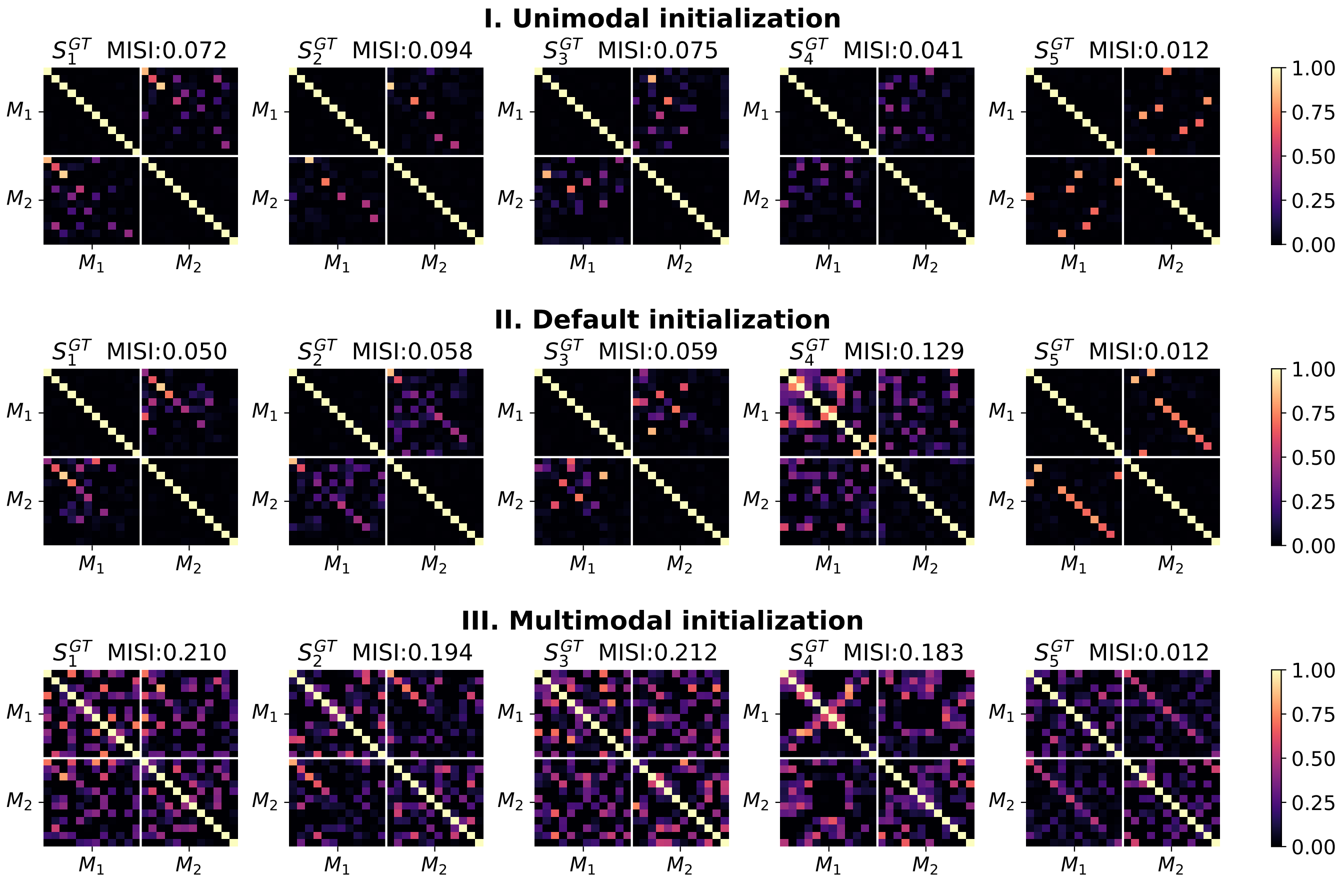

Figure S2: **Within-modal and cross-modal source correlations across initialization workflows.** Each row shows source correlations obtained with a different initialization workflow (top to bottom: unimodal, default, and multimodal). Diagonal blocks represent within-modal correlations (sources from the same modality); off-diagonal blocks represent cross-modal correlations (sources from different modalities). The default initialization workflow achieved the lowest MISI values in four of the five evaluated subspace structures (S_1, S_2, S_3, and S_5).

5 Loss curves during numerical optimization

We conducted 75 synthetic data experiments spanning 25 condition combinations (5 ground-truth subspace structures × 5 test subspace structures), each run under 3 initialization workflows. In each experiment, combinatorial optimization and numerical optimization were alternated for 10 cycles, with each numerical optimization run capped at 150 iterations. As shown in Figure S3, the normalized loss curves indicate that most numerical optimization runs converged within 150 iterations (i.e., the loss plateaued), except those using multimodal initialization, likely due to suboptimal initialization (Figure S2).

All experiments were run on a high-performance computing (HPC) node with two CPUs and 100 GB of memory. Runtime varied substantially across test subspace structures: S_5^{Test} was the fastest (under 1 minute), followed by S_1^{Test} (6-22 minutes), S_3^{Test} (16-37 minutes), S_2^{Test} (68-143 minutes), and S_4^{Test} (254–489 minutes), the latter driven by a particularly time-consuming combinatorial optimization phase.

Figure S4 shows the relationships between MISI and other metrics across five subspace structures when the ground-truth and test subspace structures are correctly matched. Normalized loss was strongly correlated with MISI under unimodal and default initialization workflows (unimodal: $R^2 \geq 0.95, p < 0.0001$; default: $R^2 \geq 0.58$, $p < 0.05$), suggesting that loss serves as a valid proxy for MISI under both workflows.

The multimodal mean correlation coefficient (MMCC), which requires ground-truth information, was strongly correlated with MISI under both unimodal and default initialization workflows (unimodal: $R^2 \geq 0.82$, $p < 0.001$; default: $R^2 \geq 0.79$, $p < 0.001$), as was the multimodal minimum distance (MMD) (unimodal: $R^2 \geq 0.69$, $p < 0.01$; default: $R^2 \geq 0.77$, $p < 0.001$). Notably, the minimum MMD value was closer to zero than the minimum $1-$MMCC value, suggesting that MMD may be a more reliable metric than MMCC.

The cross-modal metrics—the cross-modal mean correlation coefficient (CMCC) and the cross-modal minimum distance (CMD)—do not use ground-truth information and were not particularly reliable, as their correlations with MISI were inconsistent across the five subspace structures. Significant correlations were observed only for S_2 and S_4 under unimodal initialization, and for S_2 under default initialization. Among metrics that do not require ground-truth information, only loss was meaningfully associated with MISI.

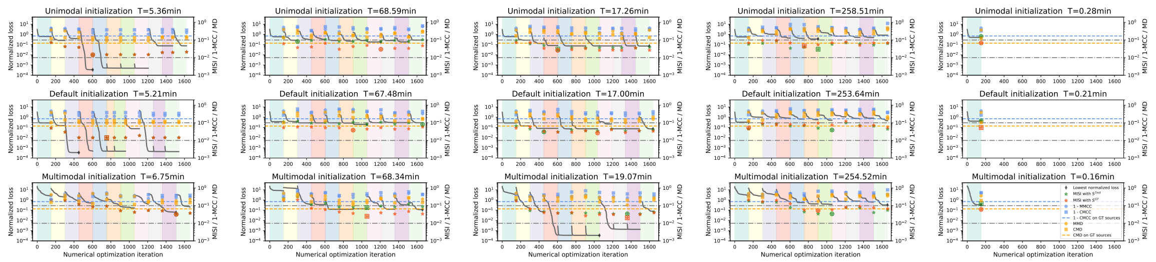

(a) S_1^{GT}, S_1^{Test}. (b) S_1^{GT}, S_2^{Test}. (c) S_1^{GT}, S_3^{Test}. (d) S_1^{GT}, S_4^{Test}. (e) S_1^{GT}, S_5^{Test}.

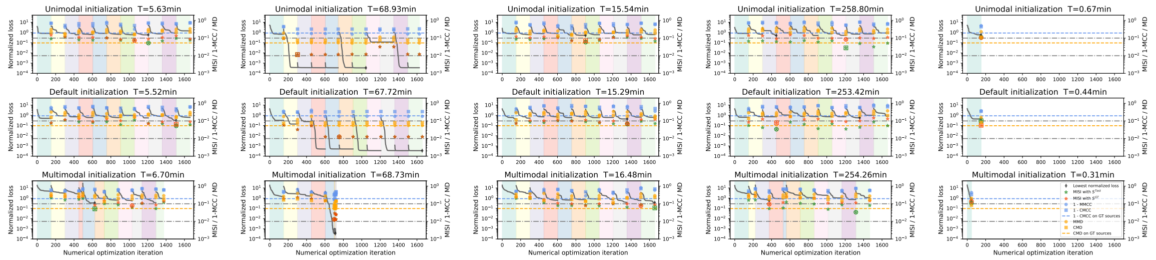

(f) S_2^{GT}, S_1^{Test}. (g) S_2^{GT}, S_2^{Test}. (h) S_2^{GT}, S_3^{Test}. (i) S_2^{GT}, S_4^{Test}. (j) S_2^{GT}, S_5^{Test}.

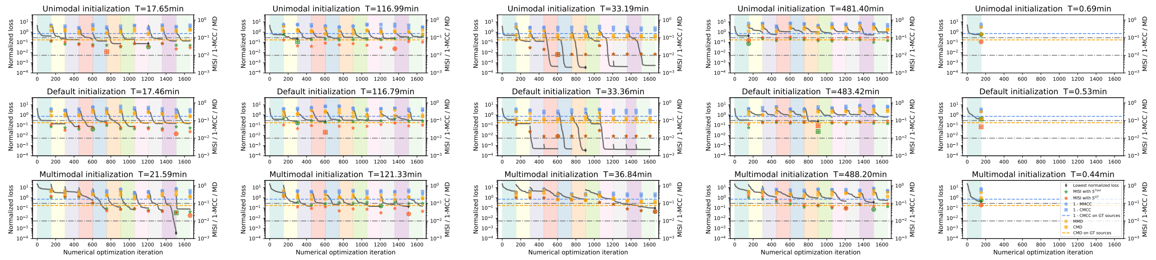

(k) S_3^{GT}, S_1^{Test}. (l) S_3^{GT}, S_2^{Test}. (m) S_3^{GT}, S_3^{Test}. (n) S_3^{GT}, S_4^{Test}. (o) S_3^{GT}, S_5^{Test}.

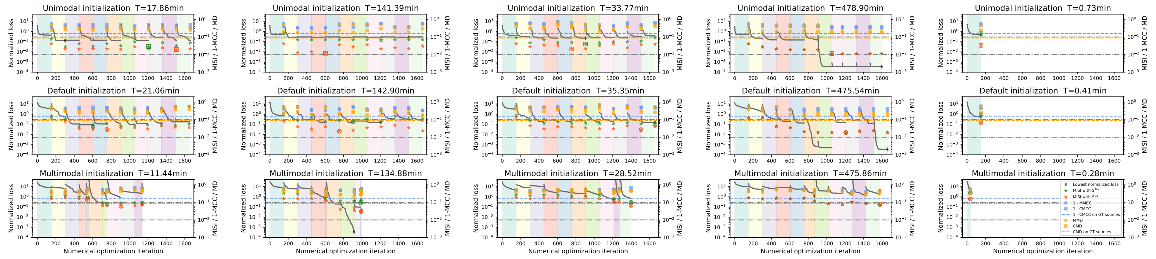

(p) S_4^{GT}, S_1^{Test}. (q) S_4^{GT}, S_2^{Test}. (r) S_4^{GT}, S_3^{Test}. (s) S_4^{GT}, S_4^{Test}. (t) S_4^{GT}, S_5^{Test}.

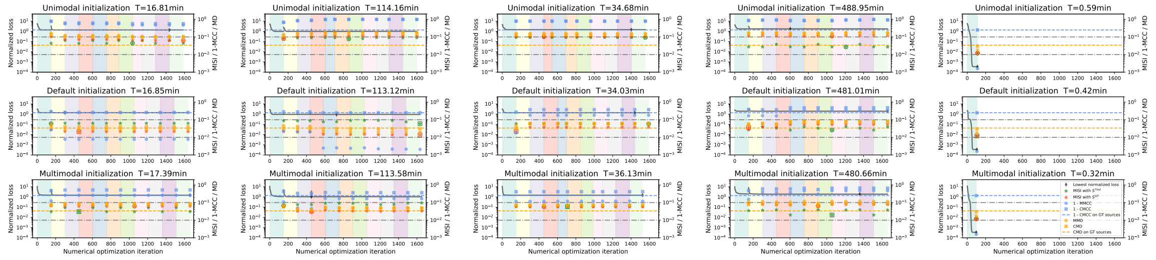

(u) S_5^{GT}, S_1^{Test}. (v) S_5^{GT}, S_2^{Test}. (w) S_5^{GT}, S_3^{Test}. (x) S_5^{GT}, S_4^{Test}. (y) S_5^{GT}, S_5^{Test}.

Figure S3: **Loss curves during numerical optimization.** Panels a, g, m, s, and y ($S^{\mathrm{GT}} = S^{\mathrm{Test}}$) confirm that only the unimodal and default initialization workflows consistently converge to the lowest loss values and detect the correct subspace structures. Vertical stripes mark the start and end of each numerical optimization run (≤ 150 iterations per run; each shown in a distinct color), with combinatorial optimization performed between successive numerical runs. The *left* y-axis shows normalized loss, computed by subtracting the minimum loss across the five test subspace structures *within the same initialization workflow*. The *right* y-axis shows additional performance metrics evaluated at the end of each numerical optimization run: MISI, $1-$MMCC, $1-$CMCC, MMD, and CMD. The lowest (best) MISI within a workflow is marked with a circle ($\bigcirc$), and the lowest MISI across all three workflows is marked with a square ($\square$).

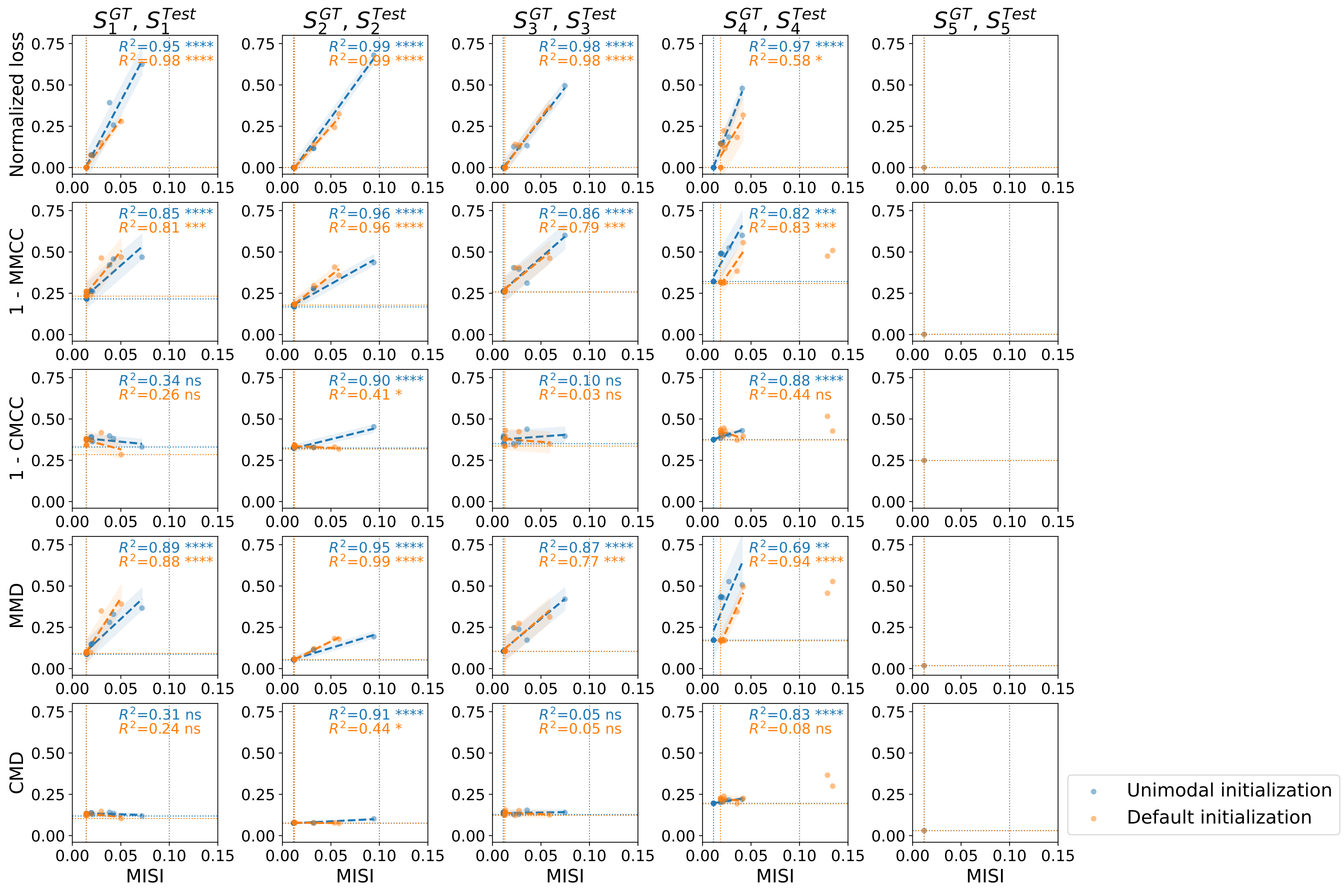

Figure S4: **Scatter plots of MISI versus other metrics.** Each row presents scatter plots of MISI versus another metric (normalized loss, $1-$MMCC, $1-$CMCC, MMD, or CMD) across the five evaluated subspace structures, for matched cases only ($S^{\text{GT}} = S^{\text{Test}}$). Each point corresponds to the result from the final iteration of a numerical optimization run. In each plot, the blue and orange vertical dotted lines indicate the minimum MISI for the unimodal and default initialization workflows, respectively; the correspondingly colored horizontal dotted lines indicate the minimum value of the comparison metric for each workflow. The gray vertical dotted line marks a MISI threshold of 0.1, commonly used as a heuristic for good source separation. A linear regression line was fitted to points with MISI ≤ 0.1, with R^2 and p-values reported. Asterisks denote significance levels (*$p < 0.05$, **$p < 0.01$, ***$p < 0.001$, ****$p < 0.0001$) and "ns" indicates a non-significant result. Normalized loss was computed by subtracting the minimum loss across the five test subspace structures within the same initialization workflow. Note that multimodal metrics ($1-$MMCC and MMD) leverage ground-truth information, whereas cross-modal metrics ($1-$CMCC and CMD) do not. Normalized loss and the multimodal metrics were all significantly correlated with MISI, whereas the cross-modal metrics were generally not, making normalized loss the only ground-truth-free measure significantly associated with MISI.

6 Order selection

To select the model order (the number of sources), we evaluated three information-theoretic criteria (Li et al., 2007) based on the singular values of the MGPCA covariance matrix derived from each multimodal neuroimaging dataset: the Akaike Information Criterion (AIC) (Akaike, 1998), the Kullback-Leibler Information Criterion (KIC) (Cavanaugh, 1999), and the Minimum Description Length (MDL) (Rissanen, 1978).

AIC, KIC, and MDL selected orders of 25, 18, and 10 for the UKB dataset, and 12, 8, and 4 for the patient dataset (Figure S5). For the patient dataset, the maximum selected order was 12. For the UKB dataset, although AIC and KIC suggested higher orders, the curves flattened at 12 or more sources, indicating that 12 sources already captured a large amount of the information. Balancing information capture against computational cost, we chose 12 sources for both datasets.

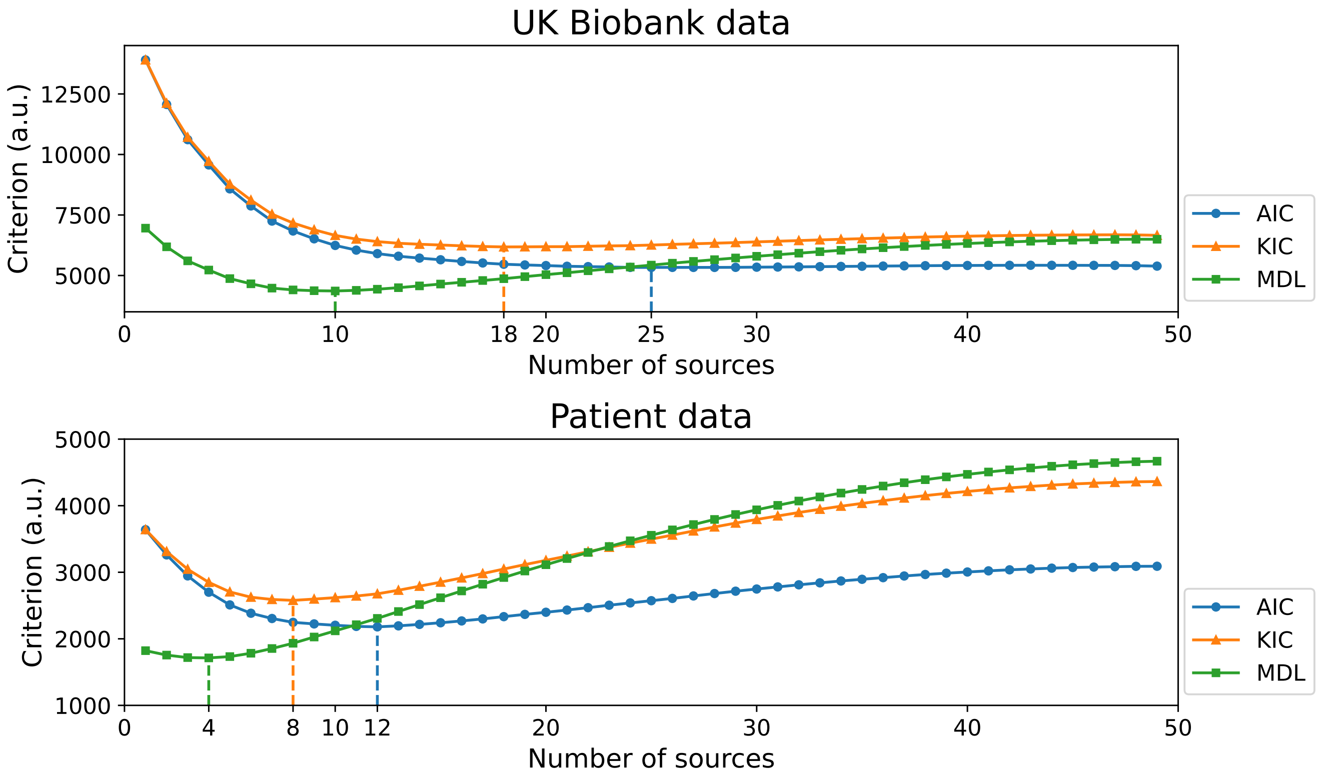

Figure S5: **Order selection for multimodal neuroimaging data.** AIC, KIC, and MDL selected orders of 25, 18, and 10 for the UKB dataset, and 12, 8, and 4 for the patient dataset. Twelve sources captured the majority of the information in the patient dataset and a substantial amount in the UKB dataset. Therefore, twelve sources were used for both datasets.

7 Nonlinear source dependence in neuroimaging data

Beyond Pearson correlation, we computed randomized dependence coefficients (RDCs) (Lopez-Paz et al., 2013) to measure nonlinear dependence between neuroimaging sources via random nonlinear copula projections. The RDC results (Figures S6a, S6b) were largely consistent with the Pearson correlation results (Figures 5a, 5b). Low off-block-diagonal RDC values indicate negligible residual dependence between subspaces, suggesting that different subspaces are effectively independent. For both neuroimaging datasets and all predefined subspace structures, the default and multimodal initialization workflows (rows VI and IX) produced stronger cross-modal correlations (higher CMCCs and lower CMDs) compared to the unimodal initialization workflow (row III).

In the UKB dataset, the multimodal initialization workflow produced notably high within-modal RDC values for sMRI within certain subspaces (e.g., the 2×2 block in S_1 and the third 2×2 block in S_2), suggesting that the corresponding source pairs are nearly identical and the underlying subspace covariance is likely ill-conditioned. Consequently, the solution from the multimodal initialization workflow is likely numerically unstable and, therefore, unreliable.

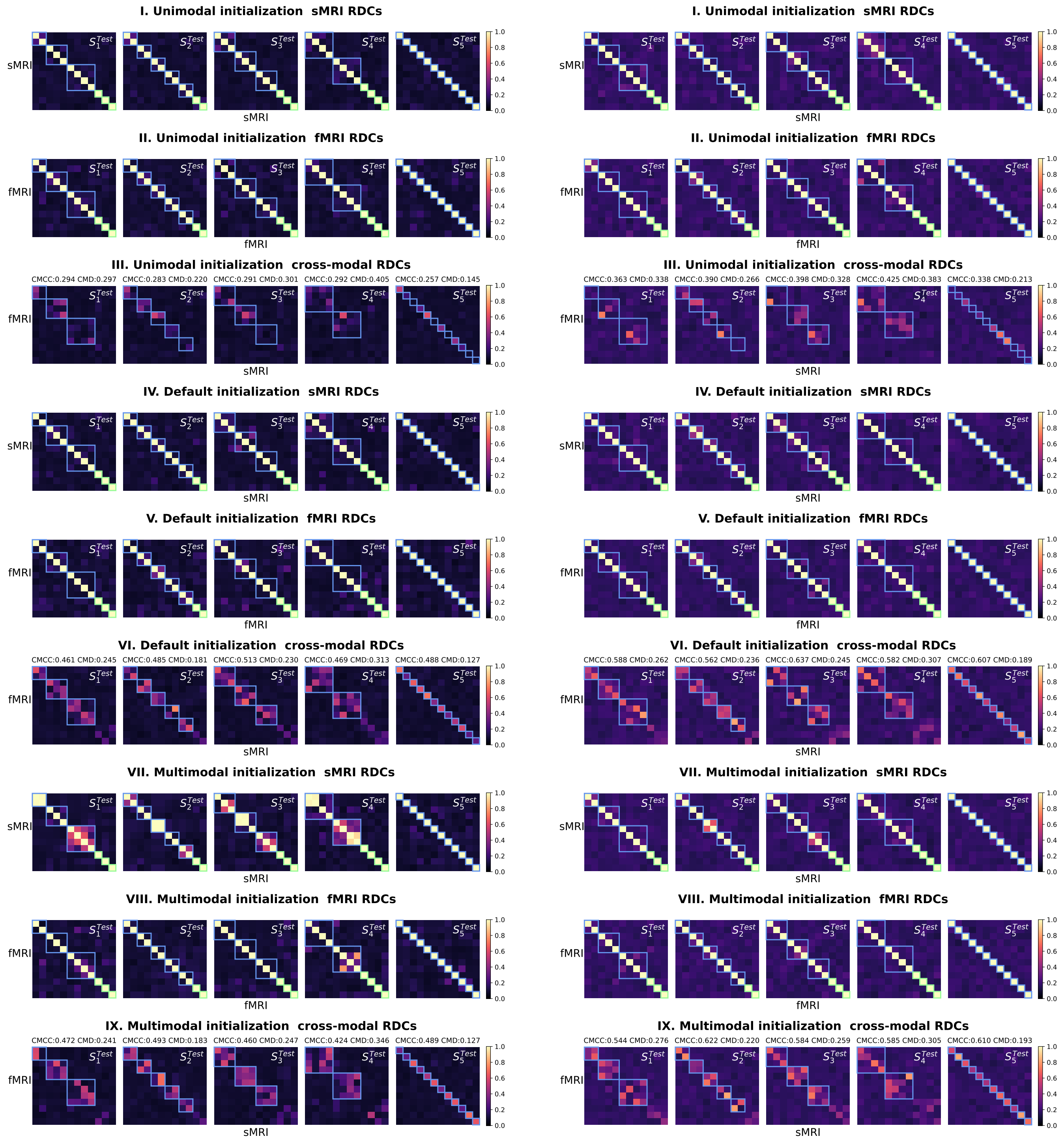

(a) UK Biobank data.

(b) Patient data.

Figure S6: **Within-modal and cross-modal randomized dependence coefficients (RDCs) of the recovered neuroimaging sources before applying post-hoc CCA.** (a) UK Biobank data. (b) Patient data. Cross-modal subspaces are highlighted in blue and modality-specific subspaces in green. Each workflow occupies three rows: within-modal correlations for sMRI (rows I, IV, VII), within-modal correlations for fMRI (rows II, V, VIII), and cross-modal correlations (rows III, VI, IX), corresponding to the unimodal, default, and multimodal initialization workflows, respectively. Within-modal correlation patterns indicated weak residual dependence between subspaces (rows I, II, IV, V, VII, and VIII). The default and multimodal initialization workflows (rows VI and IX) showed stronger cross-modal correlations (higher CMCCs and lower CMDs) than the unimodal initialization workflow (row III).

8 Sex effects in patient neuroimaging data

For the patient dataset, we assessed sex effects in the post-CCA sources from the MSIVA S_2 cross-modal subspaces (Figure S7). Sex prediction balanced accuracy was close to chance (50%) for all subspaces. Consistently, no significant sex effects were detected for any source $(p^{[1]} > 0.05, p^{[2]} > 0.05$ for all sources; two-sample t-test with Bonferroni correction for 20 comparisons).

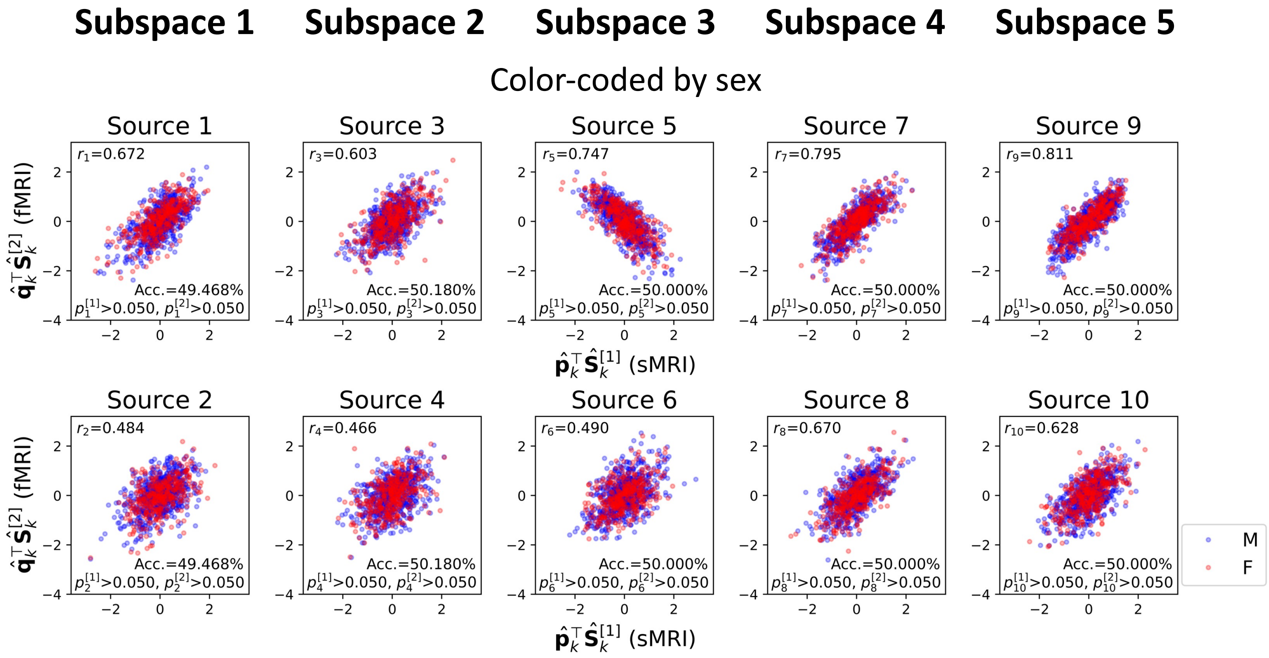

Figure S7: **Post-CCA sources from MSIVA S_2 cross-modal subspaces, color-coded by sex (patient neuroimaging data).** The Pearson correlation coefficient (r) shows the cross-modal correlation of post-CCA sources. The p-value indicates group differences between males and females for sMRI and fMRI separately ($p^{[1]}$: sMRI; $p^{[2]}$: fMRI; two-sample t-test; all reported p-values were Bonferroni-corrected for 20 comparisons). Sex effects were not significant in the patient dataset.

9 MSIVA S_2 reconstructed neuroimaging data

Figures S8 and S9 show spatial maps of group-specific reconstructed data from each of the five MSIVA S_2 cross-modal subspaces: age and sex effects for the UKB dataset (Figure S8) and age and SZ interaction effects for the patient dataset (Figure S9). In each panel, axial slices display the geometric median of the reconstructed data ($\hat{\mathbf{X}}_k^{[m]}$) for each modality and each group, with voxel intensity mapped to both color hue and opacity. Contours highlight brain regions with top 15% of voxelwise cross-modal correlations for each group (negative correlations: black; positive correlations: magenta). Histograms show voxelwise cross-modal correlations for each group (colored dashed lines: top 15% of negative and positive correlations per group; black dotted lines: $p = \frac{0.01}{44318}$, Bonferroni correction for 44318 voxels). The reported R^2 indicates the proportion of variance captured by the subspace in each modality.

Figure S10 illustrates the number of voxels with significant cross-modal correlations for age and sex groups in the UKB dataset (rows I and II) and for age and diagnosis groups in the patient dataset (rows III and IV). Notably, older patients diagnosed with SZ consistently showed fewer such voxels than age-matched control subjects in four of the five subspaces, suggesting reduced brain structure-function coupling in the older patient group.

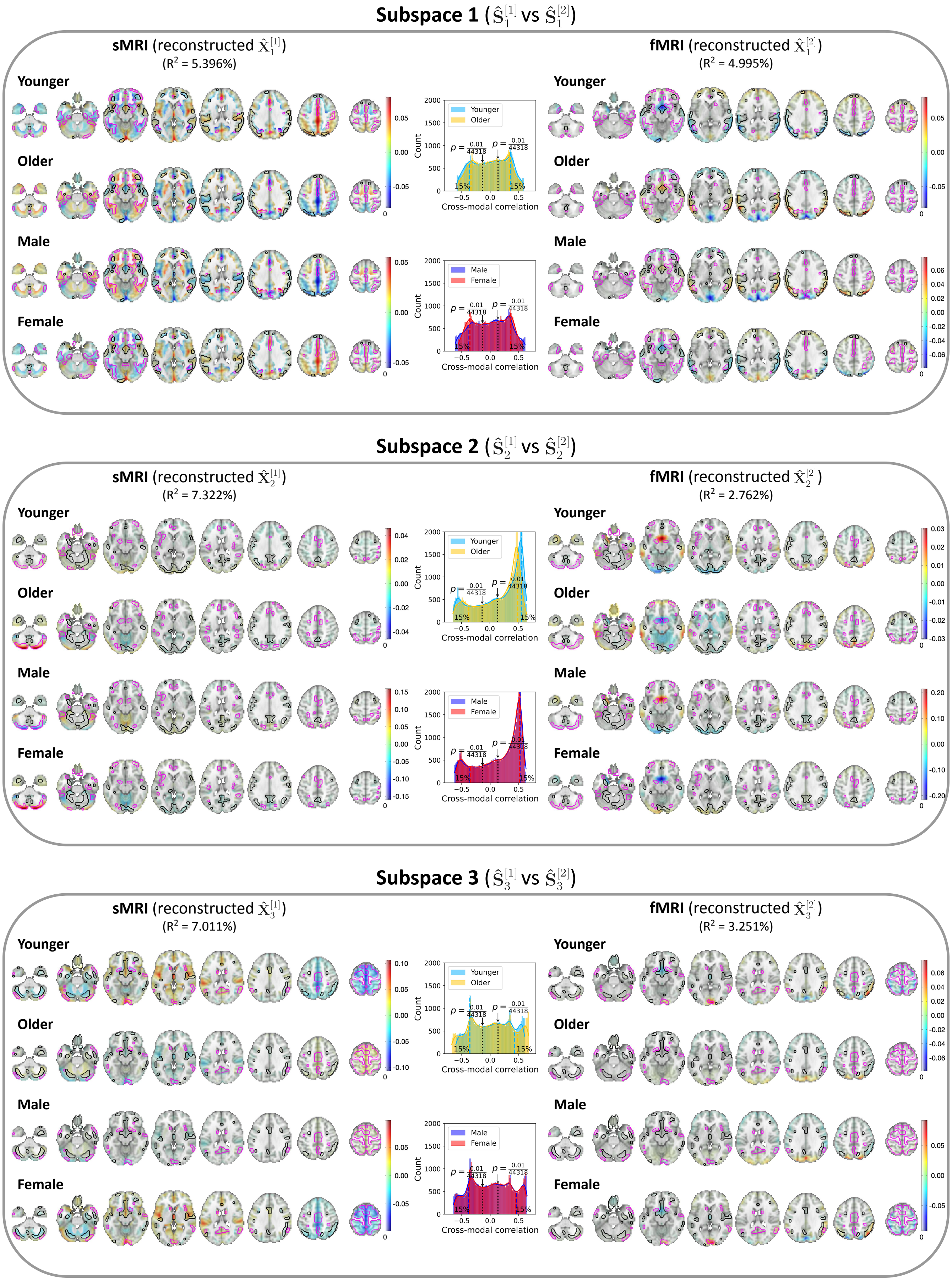

(a) Subspaces 1-3.

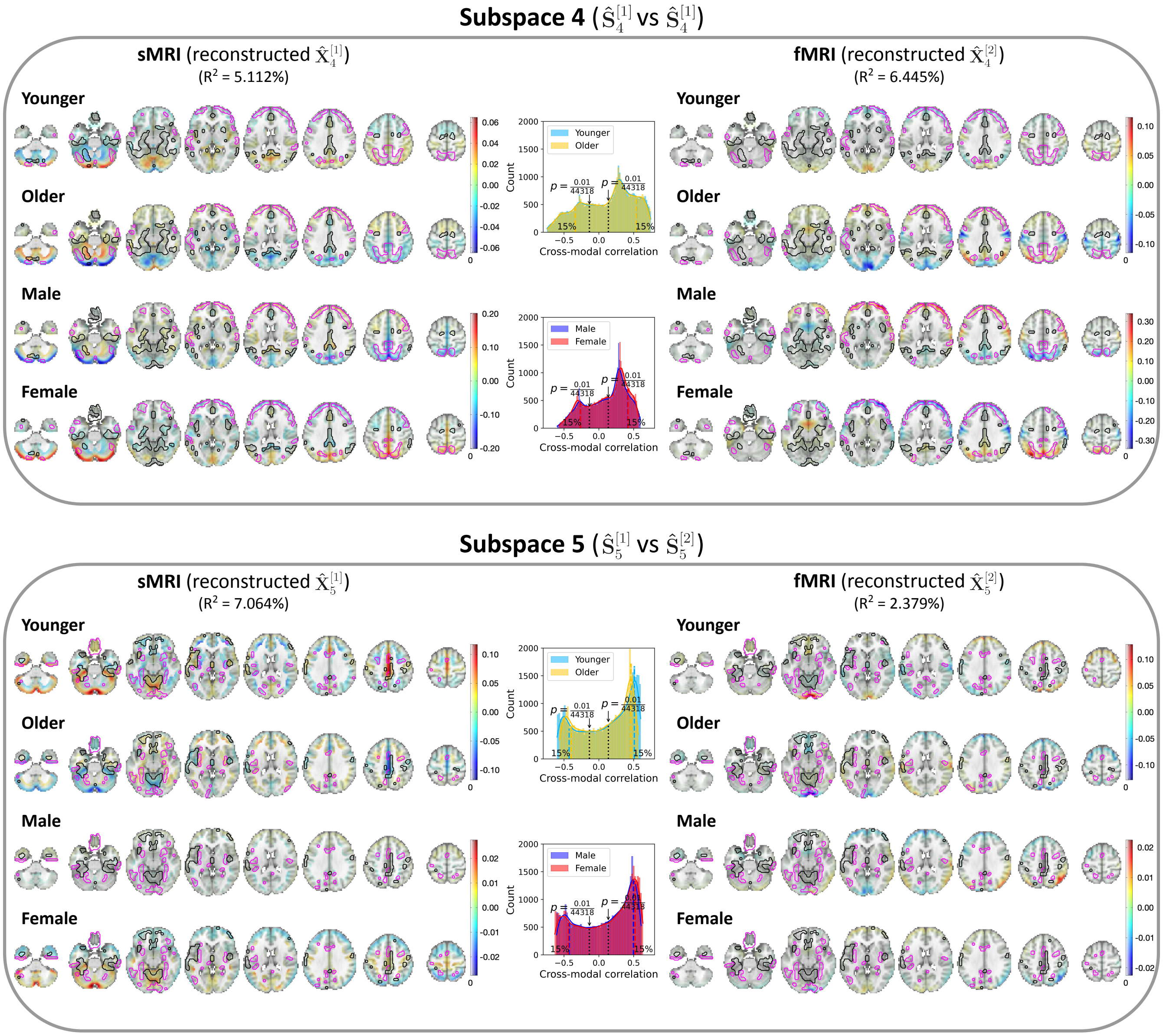

(b) Subspaces 4-5.

Figure S8: **Spatial maps of group-specific reconstructed data from MSIVA S_2 sources related to age and sex effects (UK Biobank neuroimaging data).** Axial slices show the geometric median of the reconstructed data ($\hat{\mathbf{X}}_k^{[m]}$) for each modality (sMRI, fMRI) and each group (younger: $46-62$ years, older: $63-79$ years, defined by median split; male vs. female). Voxel intensity is mapped to both color hue and opacity. Contours highlight brain regions with top 15% of voxelwise cross-modal correlations for each group (negative correlations: black; positive correlations: magenta). Histograms show voxelwise cross-modal correlations for each group (colored dashed lines: top 15% of negative and positive correlations per group; black dotted lines: $p=\frac{0.01}{44318}$, Bonferroni correction for 44318 voxels). The reported R^2 indicates the proportion of variance captured by the subspace in each modality.

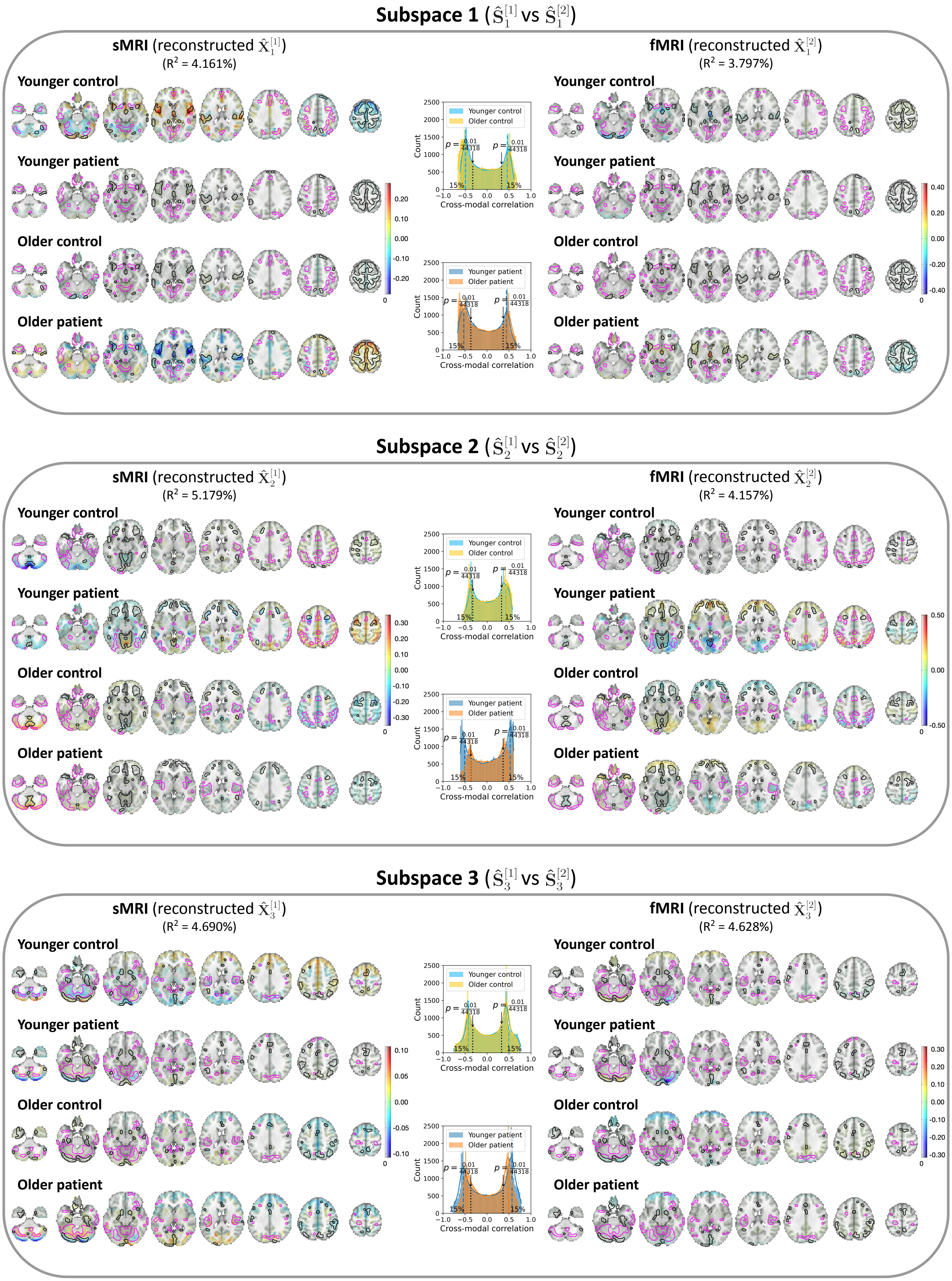

(a) Subspaces 1-3.

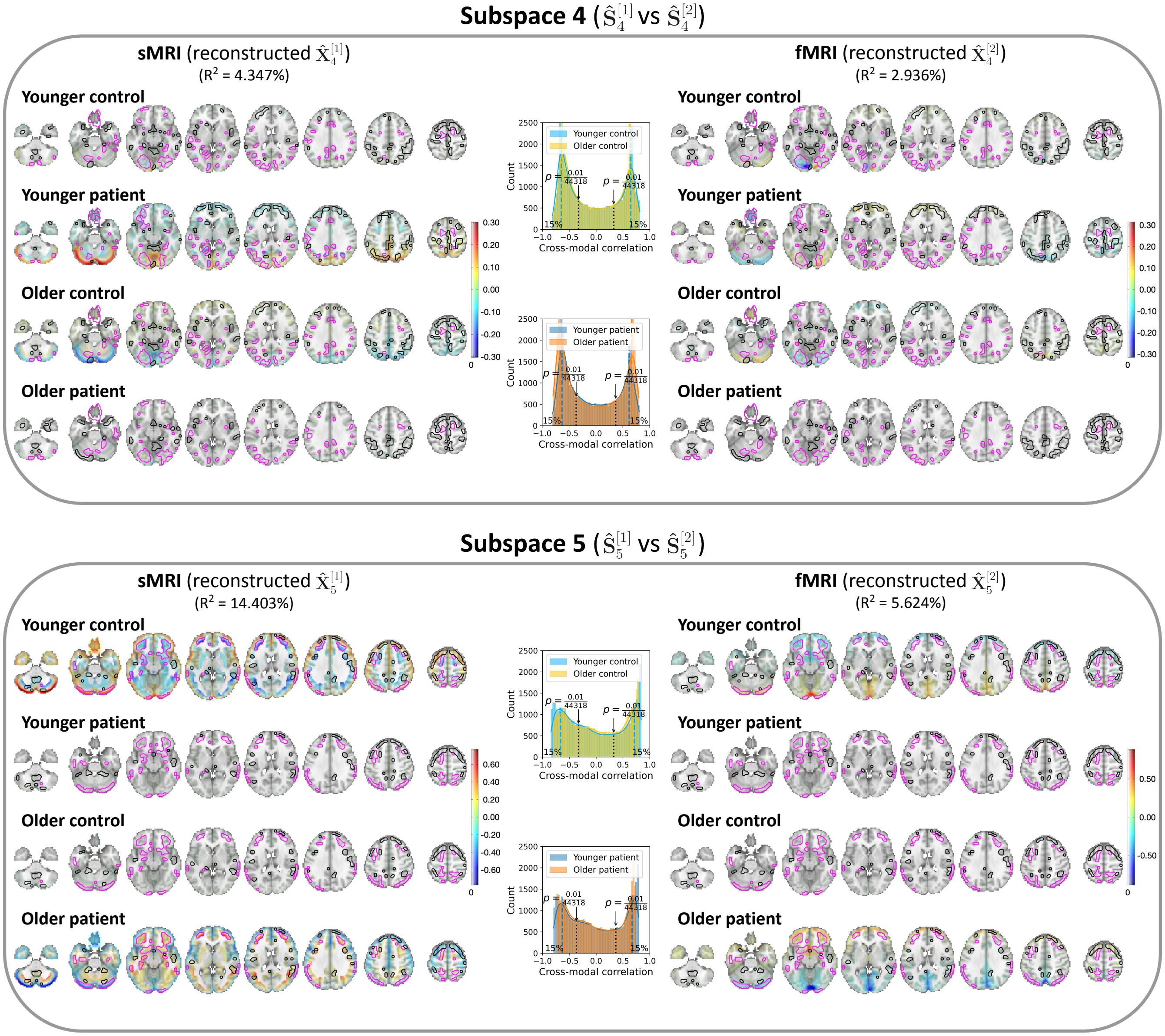

(b) Subspaces 4-5.

Figure S9: **Spatial maps of group-specific reconstructed data from MSIVA S_2 sources related to age and SZ interaction effects (patient neuroimaging data).** Axial slices show the geometric median of the reconstructed data ($\hat{\mathbf{X}}_k^{[m]}$) for each modality (sMRI, fMRI) and each group (younger: $15-38$ years, older: $39-65$ years, defined by median split; control vs. patient). Voxel intensity is mapped to both color hue and opacity. Contours highlight brain regions with top 15% of voxelwise cross-modal correlations for each group (negative correlations: black; positive correlations: magenta). Histograms show voxelwise cross-modal correlations for each group (colored dashed lines: top 15% of negative and positive correlations per group; black dotted lines: $p = \frac{0.01}{44318}$, Bonferroni correction for 44318 voxels). The reported R^2 indicates the proportion of variance captured by the subspace in each modality.

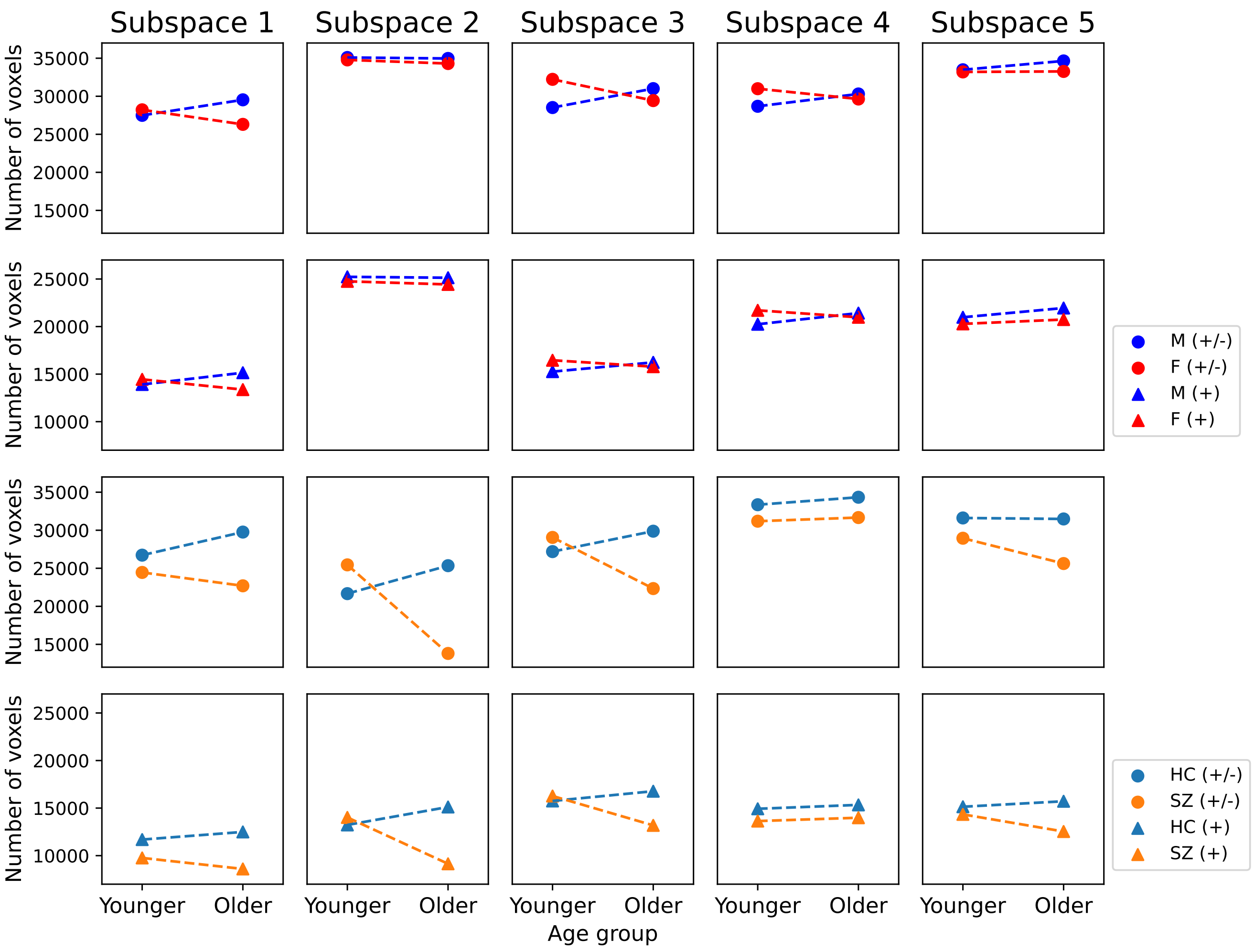

Figure S10: **Number of voxels showing significant cross-modal correlations for age and sex groups in the UKB dataset (rows I and II) and for age and diagnosis groups in the patient dataset (rows III and IV).** Rows I and III display the number of voxels with significant positive and negative correlations (+/−), while rows II and IV display the number of voxels with positive correlations only (+). The number of significant voxels for older patients diagnosed with SZ is consistently lower than that for age-matched controls in four of the five subspaces, suggesting reduced brain structure-function coupling in older SZ patients.

10 Comparison of MSIVA S_2 and MMIVA sources

10.1 Experiments and evaluation metrics

MSIVA can be viewed as an extension of MMIVA with two main differences. First, MSIVA uses a flexible *block-diagonal* subspace structure, while MMIVA uses a rigid identity-matrix subspace structure. Second, MSIVA uses MGPCA and separate ICA initialization by default, while MMIVA uses MGPCA and group ICA initialization[1] (multimodal initialization). To investigate similarities and differences between recovered sources from MSIVA and MMIVA, we compared MSIVA with the subspace structure S_2 and MMIVA with the subspace structure S_5 through the following experiments:

1. For each modality, we performed multiple linear regression (MLR) to predict each MMIVA source $\mathbf{y}_j^{[m]}$ using MSIVA S_2 post-CCA sources from each cross-modal subspace $\mathbf{X}_i^{[m]}$:

$$\mathbf{y}_j^{[m]} = \mathbf{X}_i^{[m]} \boldsymbol{\beta}, \tag{S4}$$

   where $i \in \{1, \ldots, 5\}$ is the cross-modal subspace index in MSIVA S_2, and $j \in \{1, \ldots, 12\}$ is the subspace (source) index in MMIVA.

2. For each modality, we performed multivariate analysis of variance (MANOVA) to predict MSIVA S_2 post-CCA sources from each cross-modal subspace $\mathbf{X}_i^{[m]}$ using the pair of MMIVA sources $\left[\mathbf{y}_j^{[m]}, \mathbf{y}_k^{[m]}\right]$ most predictive of $\mathbf{X}_i^{[m]}$, as identified in step 1:

$$\mathbf{X}_i^{[m]} = \left[\mathbf{y}_j^{[m]}, \mathbf{y}_k^{[m]}\right] \boldsymbol{\beta}. \tag{S5}$$

   Here, (j, k) are the corresponding subspace indices in MMIVA, and i is the cross-modal subspace index in MSIVA S_2.

3. We performed brain-phenotype prediction using two sets of sources for comparison: MSIVA S_2 post-CCA sources from each cross-modal subspace, and each pair of matched MMIVA sources identified in step 1. As described in Section 2.5, we performed age prediction and sex classification for the UKB dataset, as well as age prediction and binary diagnosis classification (controls vs. patients with SZ) for the patient dataset. Model performance was evaluated using mean absolute error (MAE) for age regression and balanced accuracy for binary classification.

[1] The initialization in Silva et al. (2024) was described as MGPCA and ICA but employed group ICA in practice.

We measured the adjusted R^2 (R^2_{adj}) from MLR:

$$R^2 = 1 - \frac{\sum_{i=1}^{N}(y_i - \hat{y}_i)^2}{\sum_{i=1}^{N}(y_i - \bar{y})^2}, \quad \bar{y} = \frac{1}{N}\sum_{i=1}^{N} y_i, \tag{S6}$$

$$R^2_{\text{adj}} = 1 - (1 - R^2)\frac{N-1}{N - N_P - 1}, \tag{S7}$$

where N is the number of samples (i.e., subjects) and N_P is the number of predictors.

10.2 Results

Figures S11 and S13 show the adjusted R^2 when using the MSIVA S_2 sources from each of the five cross-modal subspaces to predict each of the twelve MMIVA sources for the UKB and patient datasets, respectively. We reordered MMIVA sources to identify the most likely correspondence between MSIVA S_2 sources and MMIVA sources and observed partial alignment between the two. For example, for UKB sMRI data, MSIVA S_2 subspace 3 sources matched MMIVA source 3 ($R^2_{\text{adj}} = 0.96$), MSIVA S_2 subspace 5 sources matched MMIVA source 5 ($R^2_{\text{adj}} = 0.87$), and MSIVA S_2 subspace 4 sources matched MMIVA source 2 ($R^2_{\text{adj}} = 0.73$). For each row (MSIVA), more than two columns (MMIVA) showed non-negligible R^2_{adj} (> 0.2), indicating that each pair of MSIVA S_2 sources explained variance in more than two MMIVA sources. The prediction results for fMRI were consistent with those for sMRI.

Next, we performed MANOVA using each pair of matched MMIVA sources to predict the MSIVA S_2 sources from each cross-modal subspace. Figures S12 and S14 illustrate the Pillai's trace divided by the number of modalities ($M = 2$), normalizing the values to $[0, 1]$ per modality. Non-negligible off-diagonal values per column (MMIVA) implied that each pair of matched MMIVA sources explained variance in more than two MSIVA S_2 sources. The prediction results for fMRI were consistent with those for sMRI.

Finally, we performed phenotype prediction using MSIVA S_2 post-CCA sources from each cross-modal subspace, as well as each pair of matched MMIVA sources. Table S2 lists the detailed prediction performance. For the UKB dataset, MSIVA sources from subspaces 5 and 4 achieved the best age regression MAE and sex classification balanced accuracy, respectively. For the patient dataset, matched MMIVA sources from subspace 2 yielded the best age regression performance, while MSIVA sources from subspace 5 showed the best diagnosis classification performance. Overall, MSIVA S_2 sources demonstrate better predictive performance for age, sex, and SZ diagnosis than the paired MMIVA sources.

Therefore, we conclude that MSIVA and MMIVA distribute variability across their sources in different ways, with no perfect one-to-one mapping between MSIVA S_2 and MMIVA sources. The mismatch is

more pronounced in the patient dataset than in the UKB dataset, which may be related to characteristics of the patient data such as higher population heterogeneity and smaller sample sizes. MSIVA cross-modal sources more accurately predicted phenotype measures (age and sex in the UKB dataset and diagnosis labels in the patient dataset) than paired MMIVA sources, indicating that the two-dimensional subspaces in MSIVA S_2 better capture phenotype-related variability than the pairs of one-dimensional sources in MMIVA.

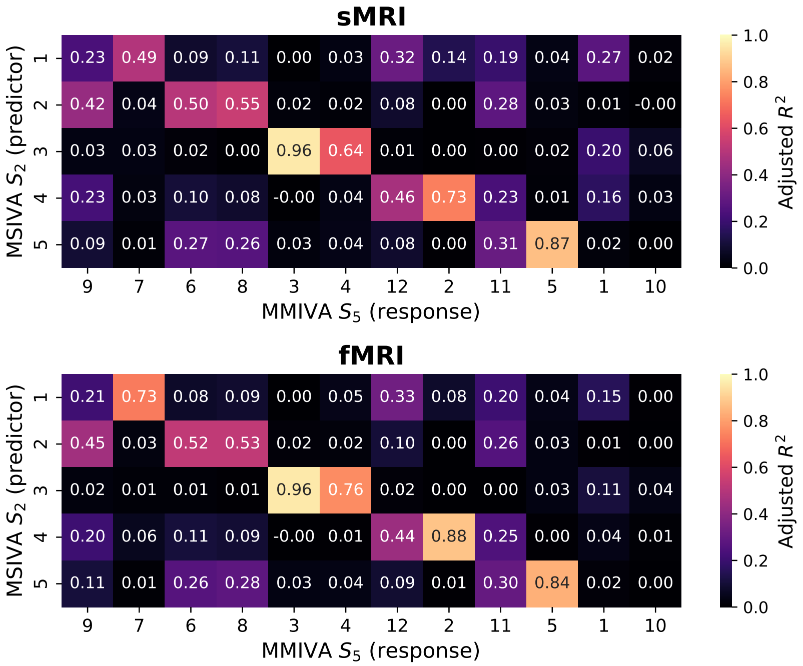

Figure S11: **Adjusted R^2 using MSIVA sources to predict MMIVA sources (UK Biobank neuroimaging data).**

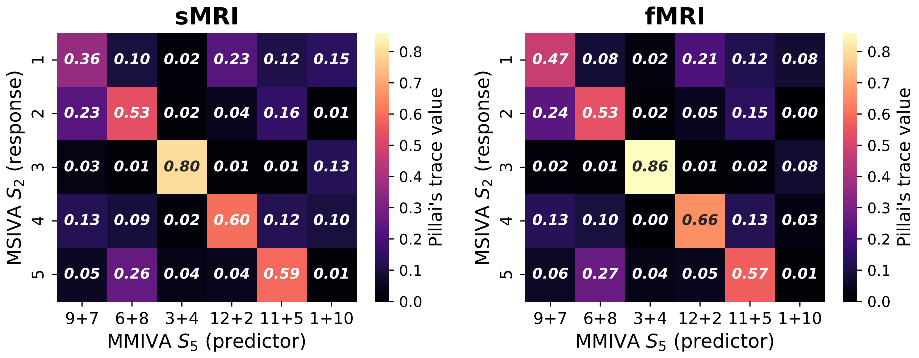

Figure S12: **Normalized Pillai's trace value using matched MMIVA sources to predict MSIVA sources (UK Biobank neuroimaging data).**

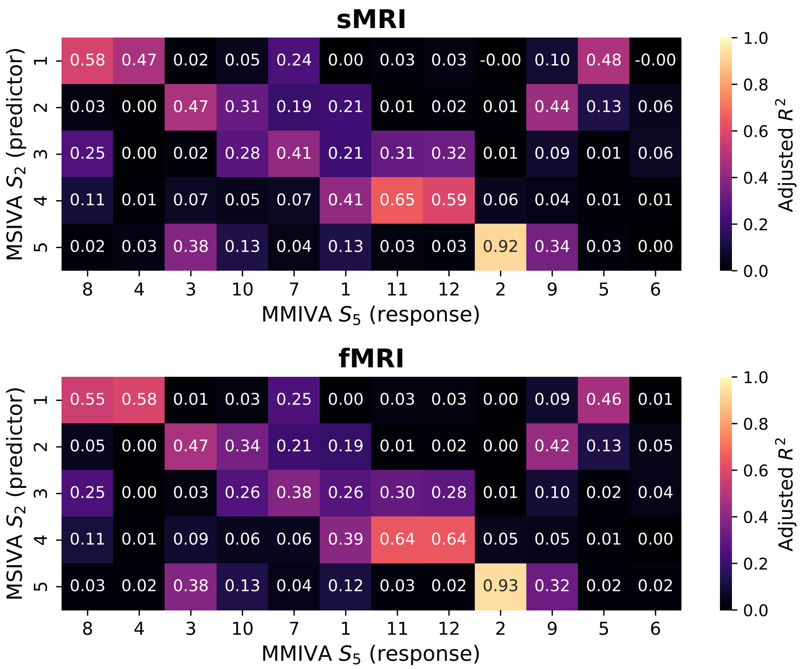

Figure S13: **Adjusted R^2 using MSIVA sources to predict MMIVA sources (patient neuroimaging data).**

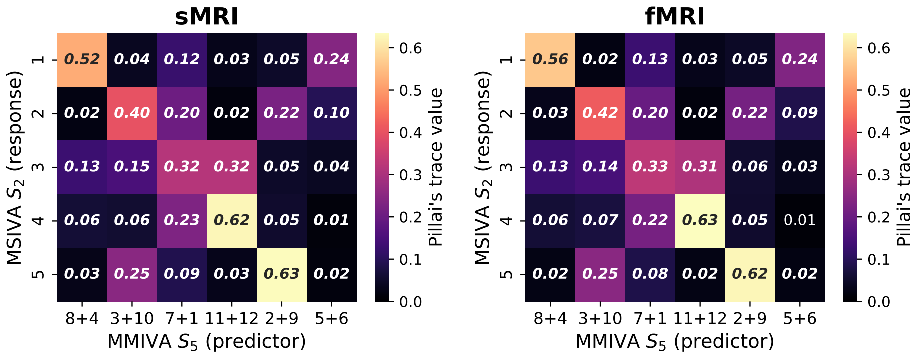

Figure S14: **Normalized Pillai's trace value using matched MMIVA sources to predict MSIVA sources (patient neuroimaging data).**

Table S2: **Phenotype prediction performance using post-CCA sources from MSIVA subspace structure S_2 and matched sources from MMIVA.** For the UKB dataset, MSIVA sources from subspaces 5 and 4 achieved the best age regression and sex classification performance, respectively. For the patient dataset, MMIVA matched sources from subspace 2 yielded the best age regression performance, while MSIVA sources from subspace 5 showed the best diagnosis classification performance. Overall, MSIVA S_2 linked sources showed stronger associations with age, sex, and SZ-related effects than the paired independent sources from MMIVA. Note that sources were estimated separately for the UKB and patient datasets, and therefore, subspaces across the two datasets do not correspond to one another.

Subspace	1	2	3	4	5
UK Biobank data					
MSIVA age mean absolute error (years)	5.674	6.163	5.892	5.847	**5.378**
MMIVA age mean absolute error (years)	6.020	5.541	6.100	6.054	5.470
MSIVA sex balanced accuracy (%)	59.542	64.496	59.206	**79.933**	52.699
MMIVA sex balanced accuracy (%)	58.874	63.729	66.483	60.085	75.415
Patient data					
MSIVA age mean absolute error (years)	10.720	10.470	11.226	11.445	10.307
MMIVA age mean absolute error (years)	10.959	**9.822**	10.903	11.679	10.835
MSIVA diagnosis balanced accuracy (%)	50.565	57.624	50.000	49.691	**61.404**
MMIVA diagnosis balanced accuracy (%)	50.752	57.747	59.919	50.000	57.695

References

Akaike, H. (1998). Information theory and an extension of the maximum likelihood principle. In E. Parzen, K. Tanabe, & G. Kitagawa (Eds.), *Selected papers of hirotugu akaike* (pp. 199–213). Springer New York. https://doi.org/10.1007/978-1-4612-1694-0_15

Alfaro-Almagro, F., Jenkinson, M., Bangerter, N. K., Andersson, J. L., Griffanti, L., Douaud, G., Sotiropoulos, S. N., Jbabdi, S., Hernandez-Fernandez, M., Vallee, E., Vidaurre, D., Webster, M., McCarthy, P., Rorden, C., Daducci, A., Alexander, D. C., Zhang, H., Dragonu, I., Matthews, P. M., ... Smith, S. M. (2018). Image processing and quality control for the first 10,000 brain imaging datasets from uk biobank. *NeuroImage*, *166*, 400–424. https://doi.org/10.1016/j.neuroimage.2017.10.034

Ashburner, J., Barnes, G., Chen, C.-C., Daunizeau, J., Flandin, G., Friston, K., Gitelman, D., Glauche, V., Henson, R., Hutton, C., Jafarian, A., Kiebel, S., Kilner, J., Litvak, V., Mattout, J., Moran, R., Penny, W., Phillips, C., Razi, A., ... Zeidman, P. (2021, October). *SPM12 manual*. The FIL Methods Group, Functional Imaging Laboratory, Wellcome Centre for Human Neuroimaging, UCL Queen Square Institute of Neurology. London, UK. https://www.fil.ion.ucl.ac.uk/spm/doc/manual.pdf

Cavanaugh, J. E. (1999). A large-sample model selection criterion based on kullback's symmetric divergence. *Statistics & Probability Letters*, *42*(4), 333–343. https://doi.org/10.1016/S0167-7152(98)00200-4

Giakoumatos, C., Nanda, P., Mathew, I., Tandon, N., Shah, J., Bishop, J., Clementz, B., Pearlson, G., Sweeney, J., Tamminga, C., & Keshavan, M. (2015). Effects of lithium on cortical thickness and hippocampal subfield volumes in psychotic bipolar disorder. *Journal of Psychiatric Research*, *61*, 180–187. https://doi.org/10.1016/j.jpsychires.2014.12.008

Griffanti, L., Salimi-Khorshidi, G., Beckmann, C. F., Auerbach, E. J., Douaud, G., Sexton, C. E., Zsoldos, E., Ebmeier, K. P., Filippini, N., Mackay, C. E., Moeller, S., Xu, J., Yacoub, E., Baselli, G., Ugurbil, K., Miller, K. L., & Smith, S. M. (2014). Ica-based artefact removal and accelerated fmri acquisition for improved resting state network imaging. *NeuroImage*, *95*, 232–247. https://doi.org/10.1016/j.neuroimage.2014.03.034

Li, Y.-O., Adalı, T., & Calhoun, V. D. (2007). Estimating the number of independent components for functional magnetic resonance imaging data. *Human Brain Mapping*, *28*(11), 1251–1266. https://doi.org/10.1002/hbm.20359

Lopez-Paz, D., Hennig, P., & Schölkopf, B. (2013). The randomized dependence coefficient. In C. Burges, L. Bottou, M. Welling, Z. Ghahramani, & K. Weinberger (Eds.), *Advances in neural information processing systems* (Vol. 26). Curran Associates, Inc. https://proceedings.neurips.cc/paper_files/paper/2013/file/aab3238922bcc25a6f606eb525ffdc56-Paper.pdf

Qi, S., Sui, J., Pearlson, G., Bustillo, J., Perrone-Bizzozero, N. I., Kochunov, P., Turner, J. A., Fu, Z., Shao, W., Jiang, R., Yang, X., Liu, J., Du, Y., Chen, J., Zhang, D., & Calhoun, V. D. (2022). Derivation and utility of schizophrenia polygenic risk associated multimodal MRI frontotemporal network. *Nature Communications*, *13*(1), 4929. https://doi.org/10.1038/s41467-022-32513-8

Rissanen, J. (1978). Modeling by shortest data description. *Automatica*, *14*(5), 465–471. https://doi.org/10.1016/0005-1098(78)90005-5

Schijven, D., Postema, M. C., Fukunaga, M., Matsumoto, J., Miura, K., de Zwarte, S. M. C., van Haren, N. E. M., Cahn, W., Pol, H. E. H., Kahn, R. S., Ayesa-Arriola, R., de la Foz, V. O.-G., Tordesillas-Gutierrez, D., Vázquez-Bourgon, J., Crespo-Facorro, B., Alnæs, D., Dahl, A., Westlye, L. T., Agartz, I., ... Francks, C. (2023). Large-scale analysis of structural brain asymmetries in schizophrenia via the enigma consortium. *Proceedings of the National Academy of Sciences*, *120*(14), e2213880120. https://doi.org/10.1073/pnas.2213880120

Silva, R. F., Damaraju, E., Li, X., Kochunov, P., Ford, J. M., Mathalon, D. H., Turner, J. A., van Erp, T. G. M., Adali, T., & Calhoun, V. D. (2024). A method for multimodal iva fusion within a misa unified model reveals markers of age, sex, cognition, and schizophrenia in large neuroimaging studies. *Human Brain Mapping*, *45*(17), e70037. https://doi.org/10.1002/hbm.70037

Smith, S. M., Elliott, L. T., Alfaro-Almagro, F., McCarthy, P., Nichols, T. E., Douaud, G., & Miller, K. L. (2020). Brain aging comprises many modes of structural and functional change with distinct genetic and biophysical associations (J. E. Peelle, F. P. de Lange, C. Madan, & L. Nyberg, Eds.). *eLife*, *9*, e52677. https://doi.org/10.7554/eLife.52677

Smith, S. M., Vidaurre, D., Alfaro-Almagro, F., Nichols, T. E., & Miller, K. L. (2019). Estimation of brain age delta from brain imaging. *NeuroImage*, *200*, 528–539. https://doi.org/10.1016/j.neuroimage.2019.06.017

Zang, Y.-F., He, Y., Zhu, C.-Z., Cao, Q.-J., Sui, M.-Q., Liang, M., Tian, L.-X., Jiang, T.-Z., & Wang, Y.-F. (2007). Altered baseline brain activity in children with adhd revealed by resting-state functional mri. *Brain and Development*, *29*(2), 83–91. https://doi.org/10.1016/j.braindev.2006.07.002

Zhao, N., Yuan, L.-X., Jia, X.-Z., Zhou, X.-F., Deng, X.-P., He, H.-J., Zhong, J., Wang, J., & Zang, Y.-F. (2018). Intra- and inter-scanner reliability of voxel-wise whole-brain analytic metrics for resting state fmri. *Frontiers in Neuroinformatics*, *Volume 12 - 2018*. https://doi.org/10.3389/fninf.2018.00054
